# A meta-narrative review of research traditions on hidden workers in aging population for transdisciplinary implementation research

**DOI:** 10.3389/fpubh.2024.1415770

**Published:** 2024-06-26

**Authors:** Sora Lee, Woojin Kang

**Affiliations:** ^1^Department of Public Health, La Trobe University, Melbourne, VIC, Australia; ^2^Department of Economics, Hanbat National University, Daejeon, Republic of Korea

**Keywords:** hidden workers, aging population, older workers, older jobseekers, older unemployed, bridge workers, older underemployed, discouraged workers

## Abstract

Hidden workers are defined as the three vulnerable subgroups of workers: the underemployed, the unemployed, and the discouraged workers. Hidden workers indeed the group with multiple identities; a transitioning retiree, jobseeker, caring for some, who may also have long term health conditions and ethnic minority all at the same time. Designing an intervention for this group necessitates the transdisciplinary knowledge. Transdisciplinary knowledge is crucial because it can inform how the intersectoral challenges might be addressed in interventions, and how the intersectoral implementation design and evaluation on hidden workers might be designed. This paper maps the intellectual landscape of the hidden workers in aging population literature to identify key disciplinary research clusters; and to find out how those research clusters are investigating hidden workers. With the meta-narrative review methodology on studies retrieved from the Web of Science Core Collection, five research clusters were identified: (1) public health approaches to hidden workers, (2) welfare state and aging workforce, (3) older jobseekers, (4) life course perspective, (5) retirement transitions. Each research cluster focuses on different aspects of hidden workers, with varying research questions and rationales. These include conceptualising the determinants of the hidden workers in aging populations and the complex interrelation with public health. Furthermore, we suggest an analytical framework to allow for better understanding between the research traditions based on (1) the chosen socioecological level of analysis, (2) whether the research question is on the determinant for hidden workers or on the outcome of being hidden and (3) the chronosystem (early/middle/later life) timeframe of research question that is addressed. Through this study, we can identify the main issues faced by hidden workers among the older adults and the measures to address these issues as well as opening up a possibility for cross-sectoral policy responses.

## 1 Introduction

The global population is ageing as the life expectancy increases. In developed nations like Australia, people can expect to live up to 83 years (1). Work and work-related activities are impacting everyday lives of older populations though various factors, including access to services, housing, income, transportation, distance, and isolation, emphasising the role of work in ensuring healthy and positive aging among older individuals. In the United States, there has been a concerning rise in the number of long-term discouraged workers among individuals aged 55 and older over the past 13 years (2). This reminds us that it is important to draw attention to a group that often goes unnoticed: the “hidden workers.” These hidden workers consist of three vulnerable subgroups within the aging workforce: the underemployed, the unemployed, and the discouraged workers (3). In many countries those without work but not actively looking for work (and/or qualified for social security benefits) are not counted as unemployed. Those who have given up looking for work. This includes those on government funded employment programmes (4, 5). Australian government is aiming for inclusive employment for all ages, that is to broaden opportunities, eliminate discriminatory practices, and address structural underutilization. This entails not only reducing barriers to work but also ensuring that individuals have equal access to employment opportunities. By doing so, we can create a more inclusive and thriving workforce (6).

While interventions targeted for unemployment are numerous, there exist a persistent distance to the labour market among the older population which calls for tailored interventions to address multifaced challenges faced by hidden workers (3). The spectrum of challenges exists because hidden workers are heterogeneous group and they have multitudes of identities. Hidden workers are indeed the group with multiple identities; a transitioning retiree, jobseeker, caring for some, who may also have long term health conditions and ethnic minority all at the same time. Designing an intervention for this group necessitates the transdisciplinary knowledge, because necessary policy actions frequently extend beyond scope of departmental portfolios and necessiate coordinated efforts through whole of the society approach. This can result in conflicting interests and divergent worldviews, which may lead to a lack of formal ownership in policies and interventions (7).

According to the National Institute on Aging, “*knowledge alone is insufficient to improve health and well-being outcomes since making clinicians, patients, and stakeholders to adapt to new standards, or intervention is complex, slow and fragmented*” [(8), p 39]. Transdisciplinary understanding for hidden workers is critical in implementation studies because it informs how the intersectoral challenges can be addressed, and how the intersectoral intervention can be better designed and evaluated. To understand better how the various elements of their lives are interrelated to their health and wellbeing, we need to first understand meta-narratives from different disciplines. Transdisciplinary research (TDR), enables the integration of knowledge from different research disciplines as well as practical knowledge from non-academic stakeholder communities (9), is pivotal in addressing the complex societal challenges such as hidden workers in aging societies. However, we still have significant barriers to carry out rigorous TDR due to distinct disciplinary systems, structures and processes (10).

Therefore, this review attempts to go beyond the understandings of hidden workers in aging population from the public health realm and venture into other disciplines addressing the population group. The objectives of this review are to find: (a) which research clusters address hidden workers in aging population; (b) how are those different clusters similar and differ in terms of research questions and the approaches; and (c) offer a transdisciplinary framework on hidden workers that can serve to facilitate intersectoral policy dialogue, and contribute to tailored intervention design and evaluation.

## 2 Methods

This research is a metanarrative review of the literature on hidden workers in the aging population, using bibliometric analysis to identify key research clusters and select publications for inclusion. Developed by Greenhalgh et al. (11), the meta-narrative review method is a type of systematic literature review approach that examines how various disciplines study a shared field of study. It has been applied to multidisciplinary topics, mostly in public health, as exemplified by studies from Kim et al. (12), Chughtai and Blanchet (13), MacLure et al. (14), and Masuda et al. (15) but first to be applied to hidden workers.

In order to provide a comprehensive review of hidden workers research, we initially employed a bibliometric analysis. The bibliometric analysis was a powerful tool to visualise research clusters. Among the bibliometric tools, co-citation analysis was performed. The co-citation analysis involves identifying co-citation relationships between papers. To be specific, co-citation relationships emerge when two papers are cited in the same publication. Also, all papers cited in a single publication have co-citation relationships with all other citations (12). By analysing the frequency with which co-cited pairs being cited together in other publications, we can infer the strength of their relationship and determine which papers belong to the same group. This concept was first introduced by Small (16), then Boyack and Klavans (17) whom provided further insights into the nature of research traditions. Through a careful analysis of co-citation network patterns, we were able to identify clusters of papers that share a common research tradition, as well as to retrieve those papers that are widely acknowledged as significant contributions within those clusters. But as we progressed, we acknowledged the constraints that came with this approach.

- Relative few numbers of highly co-cited papers (more than 20 times) indicates that there has not been a high degree of transdisciplinary collaboration established.
- The highly co-cited papers in some disciplines may be underrepresented than co-cited papers in other disciplines due to disciplinary difference in publication.
- Recently published documents are less likely to receive citations in source documents and, as a result, are less likely to be co-cited.

In order to address the first and second limitations, we have added another group of literature, the highly cited source documents (cited more than 20 times). The rationale for including the source documents with high citation is that some disciplines may be less visible in other clusters to be cited across the research clusters, but could be influential publications on hidden workers that are being recognised in their own field. While the cited references may not produce high volume of co-citation networks, the source documents and their citation network could identify another layer of disciplinary citation networks that may be received well within their disciplines, although not among the cited references. The inclusion of highly cited source documents may complement the previous processes for searching, mapping and selecting publications among the cited references. We expected the overlap between the two groups as the two groups are not inherently different groups, and we did see 20 duplicates. By carefully examining the extensively referenced source documents, one can gain a deeper understanding of the concepts, discoveries, or experiments being conveyed, as well as the significance assigned to the co-cited documents (10). We were able to identify that co-citation clusters of cited references correspond closely to aggregate word profiles of citing source documents. Therefore, this study would benefit from analysis of both cited and citing works to identify the narratives of the different research traditions.

This research is driven by a protocol that establishes the foundation for a thorough meta-narrative review. In this review, the protocol follows the well-established 6-phase sequence (planning-searching-mapping-appraisal-synthesis-recommendation) introduced by Greenhalgh et al. (18). To ensure transparency and accountability, protocol is registered in the Open Science Framework (OSF) at: osf.io/2adqk.

### 2.1 Guiding principles of meta-narrative review

This review holds the six principles of meta-narrative review. Below is the compliance with the six principles highlighting the key features:

The six principles:

- Pragmatism: Every decision regarding the search, mapping, and analysis was meticulously made with the primary objective of this review is to provide implementation researchers with a comprehensive overview of the potential framework for exploring the multifaceted approaches to studying hidden workers in an aging population. This purpose has served as our guiding light throughout every stage of this review process.
- Pluralism: In order to capture a wider range of publications that explore different perspectives on the concept of “older hidden workers,” we devised and utilised search terms that encompass various viewpoints on this population group. These terms include “aging workforce,” “old jobseekers,” “retirees,” “discouraged workers,” “bridge workers,” “underemployed individuals,” and “disadvantaged workers”—all of whom have experienced years of accumulated inequalities.
- Historicity: Through the utilisation of the document co-citation analysis method, we have successfully delved into the intellectual landscape of referenced materials and source documents, shedding light on the citing patterns of researchers from 1950 to 2023.
- Contestation: Our primary objective in this analysis was to explore how various research traditions perceive and portray hidden workers, while also recognising the distinct disciplinary assumptions they make regarding the concepts and methodologies employed to study hidden workers in aging population.
- Reflexivity: The authors acknowledge that their own perspectives can influence their decision-making during the review process and the interpretation of the findings. Furthermore, as the authors have backgrounds in public health and economics, they recognise that their disciplinary biases may impact the interpretation of the results. To mitigate this bias, they have taken steps to counterbalance the bias. These steps include cross-checking with each other while referring to the source material during data extraction and analysis.
- Peer review: Through the journal review process, findings will be presented and shared with peers.

### 2.2 Searching the literature

The search was conducted in a single multi-disciplinary database, Web of Science Core Collection (WoSCC). The maximum utility of the software of our choice, VOSviewer, is achieved when the records are collected from a single database, and in particular, the WoSCC (19). The search was conducted in the Web of Science Core Collection (WoSCC), a comprehensive multi-disciplinary database. Limiting the search to WoSCC will not significantly affect the research findings. A single database search is sufficient for bibliometric analysis. For systematic literature reviews, a precise search strategy is recommended. This involves limiting search parameters, applying exclusions, and using Boolean operators. Keyword co-occurrence analyses helped refine the search terms. Additional relevant terms were identified and added to the search strategy. Irrelevant publications (such as youth interventions) were excluded using the NOT operator. The final search term set is included in Supplementary material 1.

### 2.3 Document flow chart

**Figure 1:**
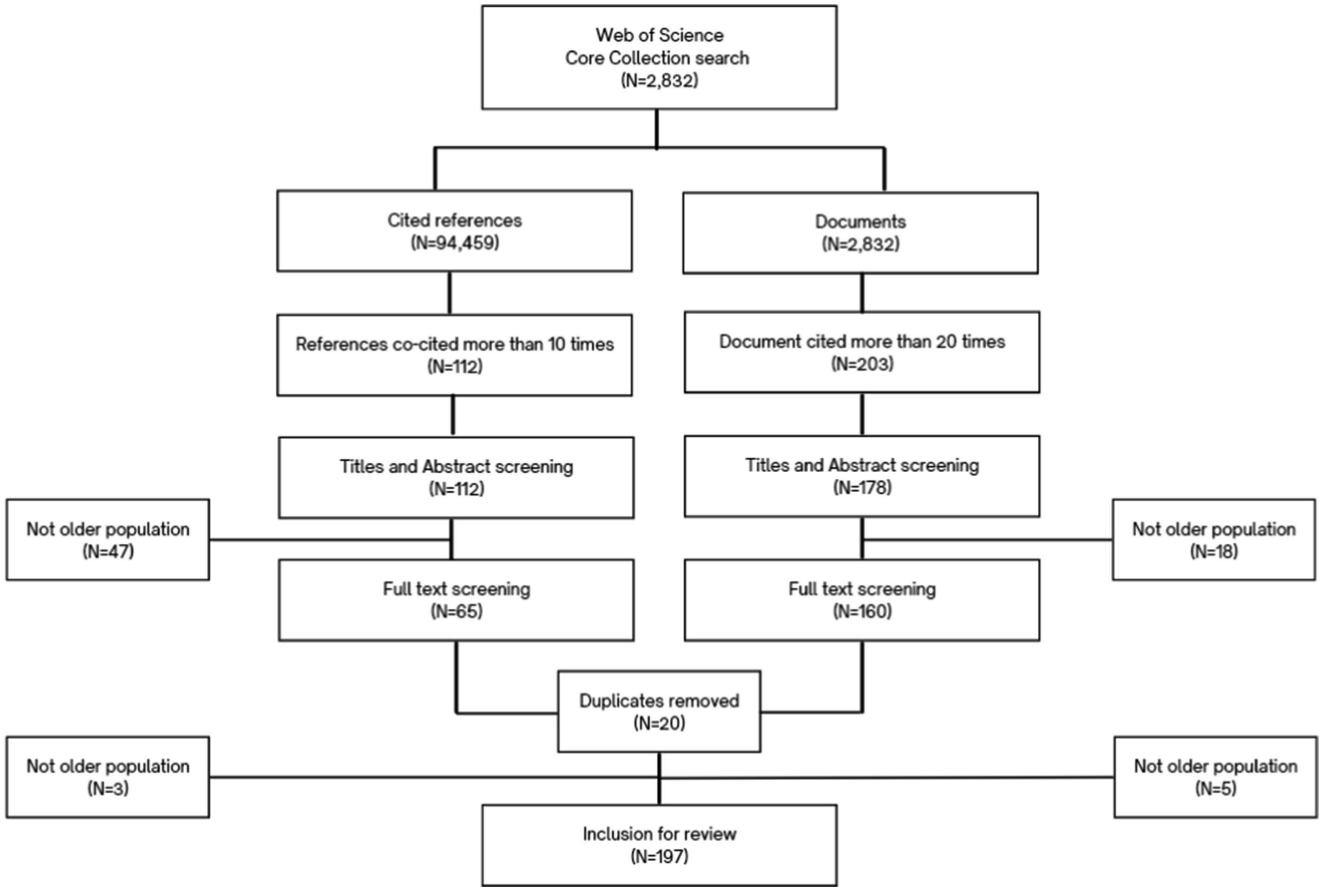
Flow chart diagram.

The process of document selection for the meta-narrative review is illustrated in Figure 1. The initial search for publications on hidden workers in aging populations in the Web of Science yielded 2,832 publications that cited a total of 94,459 references. We imported the metadata and list of references from the retrieved publications into VOSviewer software. The source data consisted of 2,832 documents, which were subjected to a document co-citation analysis. Among these cited references, we selected those publications that were co-cited by at least 10 publications (*N* = 112) for document co-citation analysis. By setting the cut-off point at 10 co-cited references, we were able to generate the same five clusters as the higher threshold, while still ensuring a manageable and adequate number of publications for inclusion in the review.

Then in parallel, we selected the source documents cited at least 20 times (*N* = 178). By setting a higher threshold, we ensured that only the most influential and impactful publications were included in our analysis. We then removed the duplicates (*n* = 20) and unrelated publications (*n* = 73). This resulted in total of 197 publications to be included in the final review. Because the scope and definition of the three concepts in the cited references were varied, an open approach to data extraction and analysis was required to capture the varying definitions and dimensions on hidden workers. Supplementary material 2 contains a table with the list of the total 197 publications included in the review and a summary of key information such as the source, field of study, type of study and the main topic of the paper.

### 2.4 Data analysis

Our aim was to delve into each cluster’s unique perspective on hidden workers and analyse their conceptualizations. It is worth noting that document co-citation analysis is based on all cited references, which means that certain highly cited publications may include papers that are not directly related to hidden workers or older hidden workers. To ensure precision and coherence in our analysis, we made the decision to exclude these publications by applying the same selection criteria that guided our literature search. In order to maintain a consistent approach to data extraction and analysis.

The publications we selected encompassed a diverse array of methodologies, populations, phenomena, scale, and analytical ambition. This extensive spectrum presented a significant challenge when it came to creating a standardised framework for data extraction and analysis. To tackle this, we meticulously reviewed each full text, extracting information on the study’s objectives, the sub-topics related to hidden workers that were addressed, the interpretation of crucial concepts, the primary findings, and the author’s key arguments. As we progressed, we iteratively conducted the data extraction process, uncovering new patterns and themes from the emerging data.

The data extraction, analysis, and synthesis were conducted iteratively. We examined the extracted data to compare between clusters and identify cross-cutting patterns and themes. During the review of the initial data, we discovered new emerging patterns and themes. To delve deeper into these themes and confirm their validity, we revisited the publications and added new items to the data extraction table. Throughout the review process, our preliminary findings were continuously validated by both the review team. This validation included joint presentations and discussions at international conferences.

## 3 Results

### 3.1 Five research traditions on hidden workers in aging population

**Figure 2:**
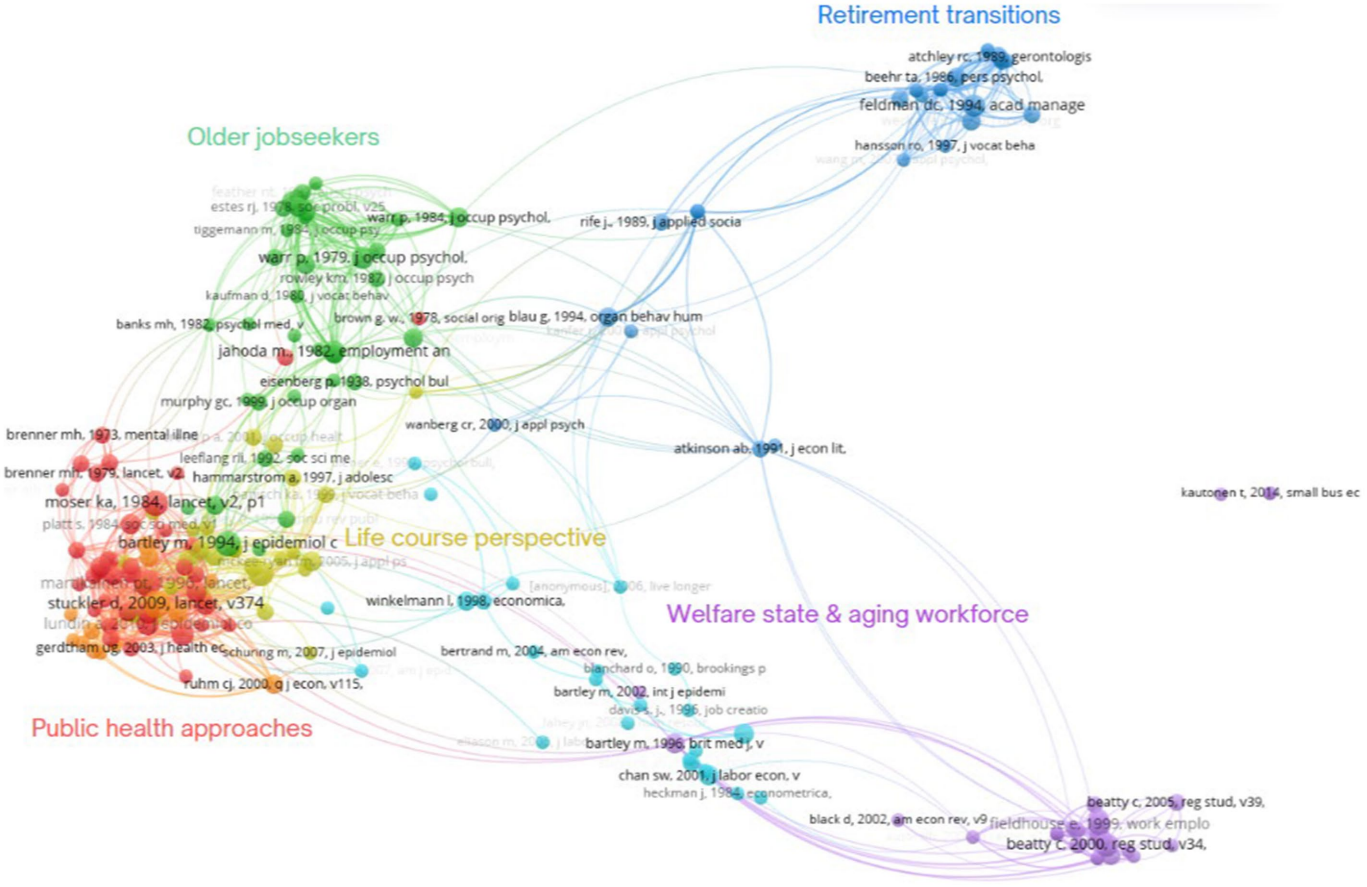
Document co-citation network analysis showing five research clusters.

The analysis of the document co-citation network resulted in the identification of five distinct research clusters, each representing a unique research tradition. Figure 2 presents the document co-citation pattern based on the cited references. The size of circle characterises number of citations, and the lines show the strength of co-citation relationship. The colour shows how different research clusters are closely related with each other.

After close examination of the 197 publications that directly addressed the topic of hidden workers in aging populations, we have named the five clusters as shown below: (1) public health approaches to hidden workers, (2) welfare state and aging workforce, (3) older jobseekers, (4) life course perspective, (5) retirement transitions. The terminology was derived by extracting the keywords used by the clusters that symbolically capture the essential conceptualisation of hidden workers for each group. These names not only signify the research tradition but also differentiate the clusters from each other. However, it is important to acknowledge the potential risk of oversimplification associated with these designated names.

Each cluster offers a unique perspective on how research studies conceptualise hidden workers and associated health and wellbeing implications. The network visualisation reveals different network densities within the clusters, as well as varying strengths of connections between different groups. In the upcoming sections, we will provide a comprehensive overview based on their conceptual understanding, research questions, health and wellbeing implications, layer of analysis and implementation perspectives. Table 1 presents a concise summary of the aforementioned dimensions. Subsequently, we will delve into each cluster’s approach to addressing hidden workers in more detail.

**Table 1:** Summary of the five research clusters on interventions for hidden workers in aging population.

	Public health approach	Welfare state and aging workforce	Older jobseekers	Life course perspective	Retirement transitions
**Number of papers included in the analysis**	63	58	36	27	13
**Geographical region (First author affiliation)**	USA, Spain, Australia, Canada, France, Germany, Netherlands, England, Sweden, Norway, Poland, Denmark, Finland, New Zealand, Scotland	USA, Netherland, Germany England, Denmark, France, Canada, Sweden, Scotland, Finland, Belgium, Switzerland, Wales	Italy, Denmark, USA, Switzerland, Australia, Scotland, Israel, Canada, England	USA, Germany, China, Sweden, Greece, Scotland, Spain, Brail, Taiwan, Finland	USA, Ireland, Chile, Australia, Canada, South Korea
**Type of study**	Empirical (cross sectional/ longitudinal/ cohort study) Review	Empirical (cross sectional/ longitudinal/ cohort study/mixed methods) Theoretical Review Policy evaluation	Conceptual Empirical (cross sectional/ longitudinal/ qualitative) Review Policy evaluation	Empirical (Cohort, cross-sectional/ longitudinal/ ecological) Review	Empirical (cross-sectional /longitudinal/ qualitative) Conceptual Policy evaluation
**Field**	Psychology, Public health, occupational health, Business & Economics, Social Science, Geriatrics	Business & Economics, Public Administration, Social Work, Occupational health, Biomedical Social Science, Geriatrics, Demography, Sociology	Psychology, Occupational health, Business & Economics, Medical science, Geriatrics	Geriatrics, Social science, Psychology, Occupational Science, Social Work, Health Services	Business & Economics, Psychology
**Key concept for hidden workers in aging population**	Older individuals with specific health traits or health behaviours that may limit one’s opportunities in labour market	Older individuals who want to be economically active but facing structural problems	Older individuals facing discriminations in job search, recruitment process, or have difficulty keep working	Older individuals with accumulated experiences of inequalities due to earlier life factors	Older individuals who are choosing their later working life patterns as they see fit and have available
**Research questions**	What is the public health impact of unemployment?	What is the optimal retirement age and how can policies and intervention induce the change?	What are the difficulties faced by older jobseekers during the job search/job loss and what influences their decision to re-enter the labour force?	What are the earlier life determinants for hidden workers and what are the lingering impact?	What are the various retirement pathways among older population? How can the individual decide on the working pattern on one’s later life for one’s best interest?
**View on the relationship between health and unemployment**	The unemployment poses great burden on public health and healthcare system.	Public policies are important elements to influence the decisions of labour market which in turn contributes to more structural opportunities for healthier aging population.	There can be barriers in job search for older jobseekers.	Earlier life health conditions can be determining factors in future job status.	One retirement pathways are becoming diverse and dynamic.
**Level of analysis**	Population sub-group/ region / country	Country/international	Individual, firm-level	Cohorts	Individual/country
**Suggested interventions for hidden workers**	Patient/treatment -specific intervention programmes	Public pension, social security policies	Firm’s hiring policies, non-ageist climate for older jobseekers	Earlier interventions to reduce accumulated inequalities	Policies/ interventions to expand one’s options to retirement

Table 1 Summary of the five research clusters on interventions for hidden workers in aging population.

## 4 Conceptual framework for transdisciplinary research

### 4.1 Developing a framework of TDR for older hidden workers

The significance of this meta-narrative review lies in its potential to develop a framework that can identify the fundamental differences among different research traditions. This framework acts as a catalyst for fostering transdisciplinary understanding between these traditions, by precisely identifying the specific areas where their perspectives on hidden workers in the aging population diverge. As mentioned earlier, transdisciplinary understanding starts with cultivating shared interests in the knowledge that exist within each research tradition.

Across the five research traditions, there were variations in how hidden workers were approached in terms of concepts, theories, methods, and instruments. However, we did identify some common themes that cut across these approaches. They include the phenomenon of unemployment and the widely associated factors that contribute to individuals becoming hidden workers within the broader socioecological sphere. Additionally, there is a focus on understanding whether change is driven by structural factors or individual agency, as well as the specific timeframe in which these research questions arise (whether it is earlier, middle, or later in life). These three dimensions also present opportunities for transdisciplinary collaborations.

#### 4.1.1 Identification of determinants of becoming hidden workers in their later life

**Figure 3:**
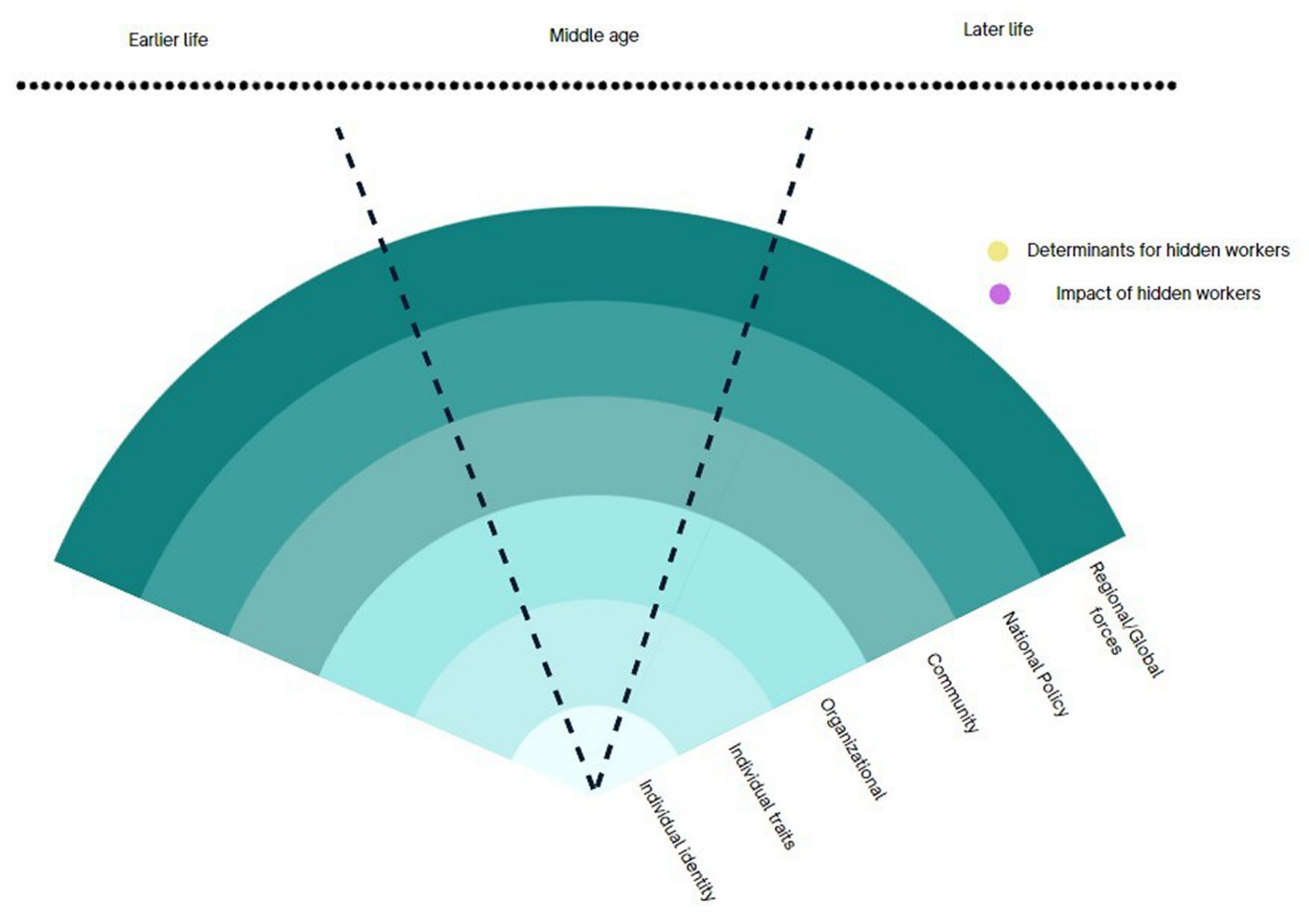
Conceptual framework for TDR on hidden workers.

The potential for transdisciplinary collaboration lies in exploring each discipline’s unique perspective on the factors that contribute to hidden workers or the consequences of being a hidden worker (Figure 3). Across the five clusters, it is widely accepted that the pathways of becoming hidden workers are complex with multi-directional interactions between various determinants from different socioecological levels. The scope of research on older hidden workers encompasses all levels, ranging from individual (identity/traits) through organisational, community, policy, all the way to the global forces. Most research traditions recognise the complex interplay of forces at different levels, but they tend to prioritise specific levels within the socioecological framework.

#### 4.1.2 Distinction between determinants of outcome

A second dimension is the difference in the research question. Across the research traditions on older hidden workers, we observed shifting of the focus: on one hand, there are research questions aims to understand what the determinants of hidden workers and on another hand, the emphasis is on its impact on individuals and society. The former ranges from identifying the features of the structures that contribute most to becoming hidden workers to individual traits that influence their employment status. For structural features: (a) the greater social structures (policies, aging rate, economic growth) or exogenous shock or trend (COVID-19, automation) influence older individual’s pathway into hidden workers. (b) the social structures and exogenous shock or trend and agents within the system influence decisions that shape the patterns of their later working lives. For individual features, they include (a) individual identity, (b) individual physical mental health status, (c) individual characteristics (d) individual behaviours and actions. The structural perspective focuses its attention towards policies and regulations as the central point for instigating change. On the other hand, the agency-focused perspective highlights the crucial role of actors, firms, and the labour market in shaping and revolutionising their choices and behaviours.

#### 4.1.3 The chronosystem: timeframe (earlier, middle, later life) of the research questions

The third area brings the chronosystem for hidden workers. What is evident in the five research traditions is that, we observe two fundamentally different temporal aspects to understanding the hidden workers—one that views becoming hidden workers in their later life as a consequence of public policies that directly influences them at their later life, and another that takes a life course perspective. The former view, usually in the welfare state and aging workforce research tradition, identifies the components of regulatory means and labour market incentives in order to evaluate the policy impacts. For example, the later life intervention perspective might involve the evaluation of age pension or retirement age delay on the aging population’s decision to participate in the labour market.

In contrast, the life course perspective encompasses the early traits of disadvantage, accumulated inequalities. The lived experience of individuals and their impact on later working lives. This is most common among the public health sub-discipline of life course epidemiology where longitudinal cohort studies with population with a specific disadvantageous trait, such as disability or health condition, are undertaken to evaluate their later life opportunities or consequences. While the two opposite research traditions provide the two extremes on the chronosystem, we observed contrasting assumptions in each research traditions in their timeframes of research questions. This will be further explored in the following section.

## 5 Mapping the three dimensions to the five research clusters

With these three dimensions in mind, we have crafted a comprehensive framework that not only enhances our understanding of the five research traditions but also aids in identifying promising avenues for transdisciplinary knowledge exchange. All the empirical, non-empirical research publications in each category were mapped except for the reviews. For studies that deals with multiple levels, the most significant finding was mapped.

### 5.1 Public health approach to hidden workers

**Figure 4:**
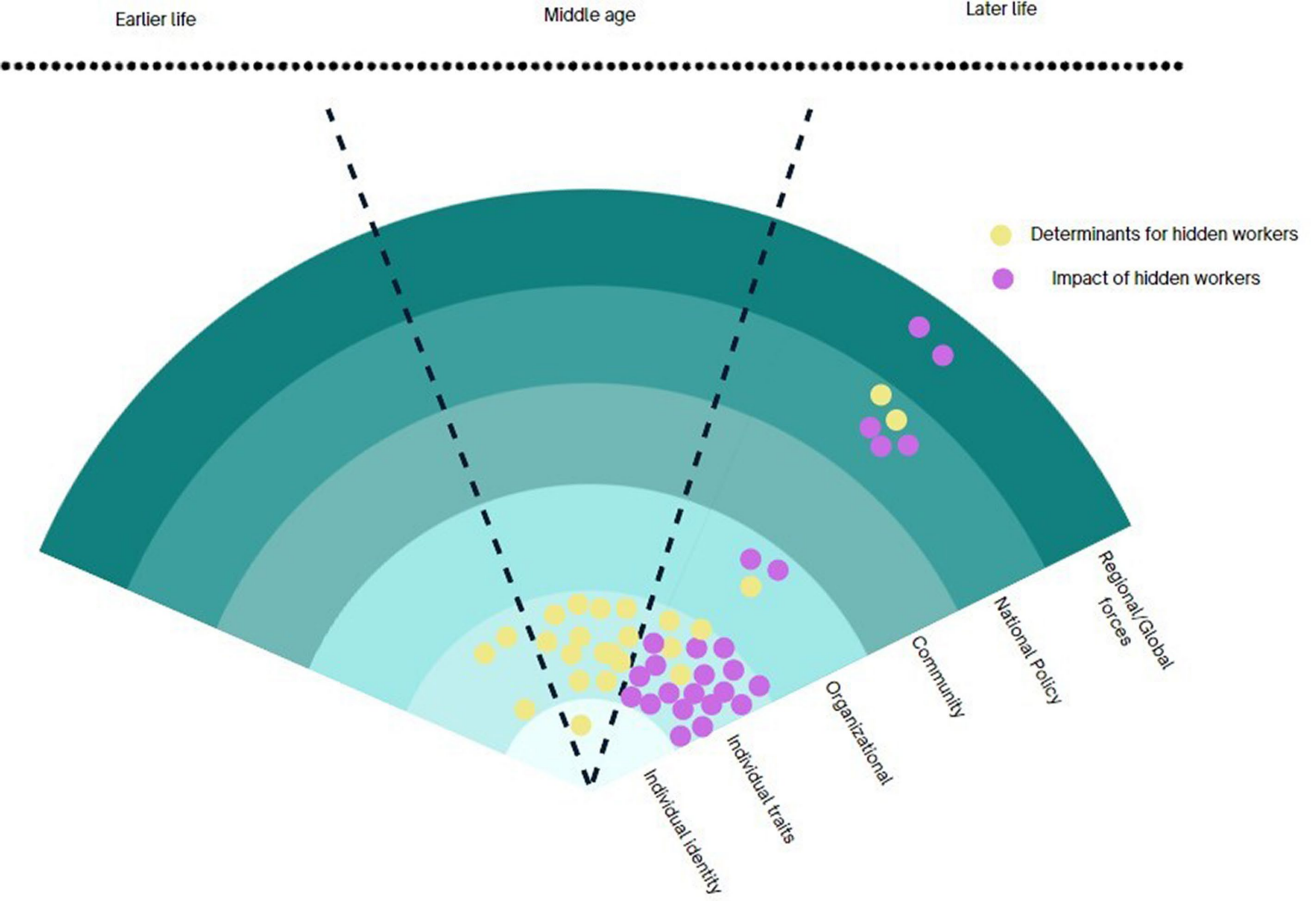
Public health research cluster.

The first cluster of hidden workers discusses public health research cluster on older hidden workers is shown in Figure 4, where health impacts of unemployment are dominant forms of research questions. The health conditions, behaviours (alcohol or substance use), patient/treatment groups were often examined to identify their extent of limitation in employment opportunities, as well as investigating health impact of unemployment or exclusion from the labour market. This research tradition is prominent in public health disciplines particularly in mental health, depression and in suicide research. There are some overlaps with the first cluster as some policies, such as disability benefit and sickness allowances are of key interest in this group.

This cluster is predominantly focused on whether this association is causal from unemployment to health (20). For instance, there are suicide and mortality studies usually study the impact of unemployment at the regional, country-level or international comparison using longitudinal study design (21–24). Additionally, these studies highlight the importance of social support in aiding individuals in effectively dealing with the challenges of unemployment. Global forces considered in this cluster is COVID-19 and were examined in the context of employment and depression (25, 26).

Research in this cluster is also predominantly concerned with health selection on unemployment, which is a concept that health can also result in increased likelihood of job loss and a decreased likelihood of finding employment once unemployed (27–29) This is highly contested topic and the evidence is mixed. Some researchers argue that social position is influenced by health selection, while others emphasise the role of social causation and downplay the impact of health selection. In the field of economics, poor health is often cited as the primary reason for retirement (30–32). Occupational epidemiology supports the theory of the “healthy worker effect,” which refers to the process of selecting employees based on their health status (33–36). Many studies in this research cluster identify the employment status of the subpopulation group and study their impacts on physical and mental health. For example, Kordovski et al. (37) report on the adverse employment outcomes of older HIV positive adults and Castro-Marrero et al. (38) showed the increased risk of work disability among the individuals with chronic fatigue syndrome.

### 5.2 Welfare and aging workforce

**Figure 5:**
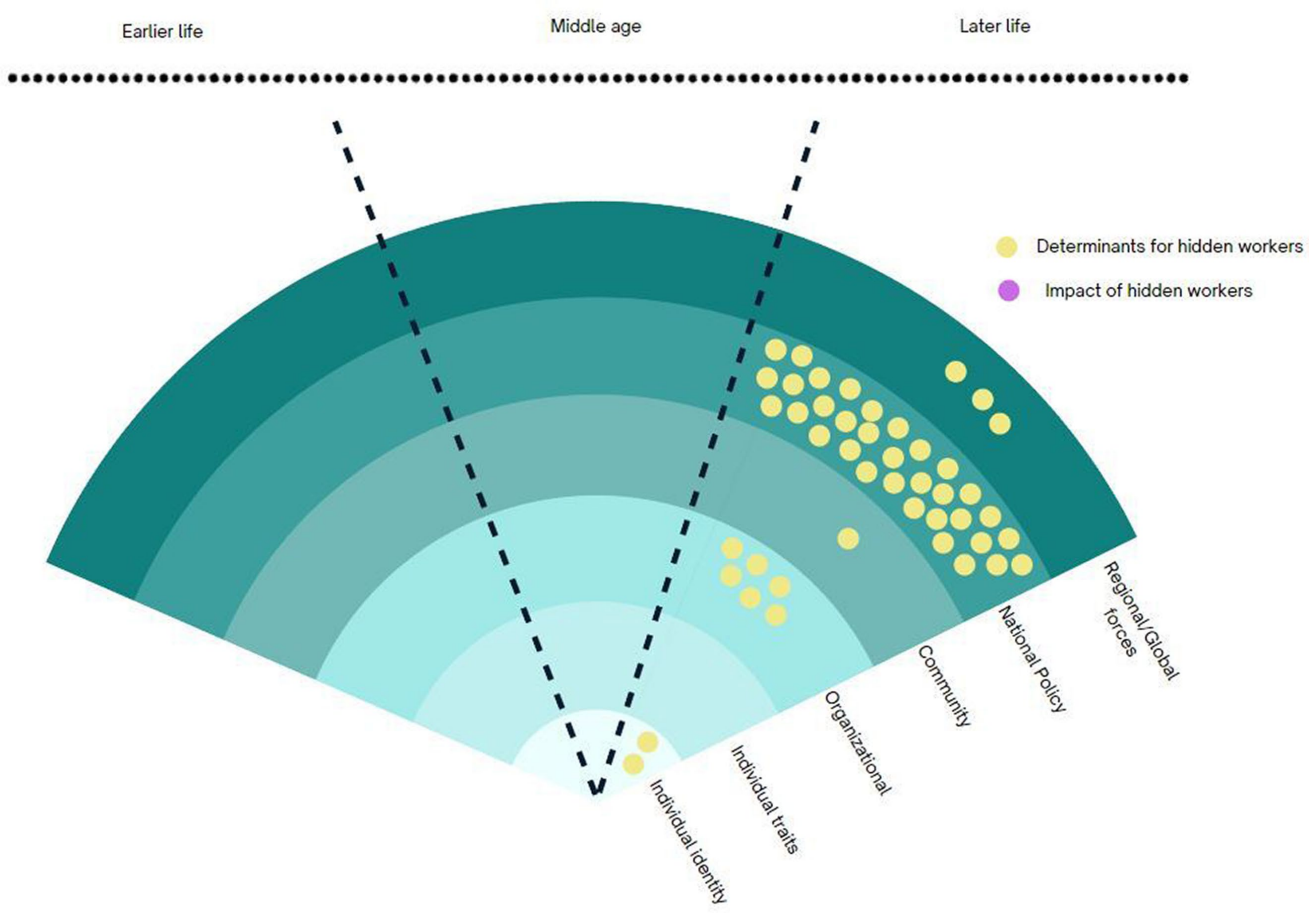
Welfare and aging workforce research cluster.

This research cluster of hidden workers in aging population, shown in Figure 5, is based on the concept that emphasises the rapidly aging societies and the regulatory means to prepare and anticipate the labour market consequences or welfare cost. Esping-Anderson’s groundbreaking publication, “The Three Worlds of Welfare Capitalism” (1990), deserves special attention in this research tradition. His argument that current economic processes, including the labour market responses on the aging population, are shaped by the states using various regulatory means, including social security benefits and public pension schemes. Therefore, studies in this research cluster generally regard ‘state’ as a political and administrative unit of analysis for various policies. This group mainly consists of studies from economic and regulatory disciplines and concerned with the policy impact on labour market outcomes.

Public policies play a crucial role in achieving the objective of empowering older workers to remain engaged with the workforce for longer. Public policies on unemployment and the subsequent health impacts are also widely studied. Key policies studied are unemployment insurance, age pension, retirement age, sickness allowance, and the deletion of mutual benefit obligations (39–44). Policies have been evaluated in terms of its impact on individual decision on retirement age, benefit recipient status. Policies can also impact firms’ decision making: pension reform can create incentives for firms to avoid encouraging individuals’ early retirement benefits (45). Aging workforce is global phenomenon and finding out the optimal retirement age is a task for every country (46).

While unemployment and early retirement are often raised issues in this cluster, it is worth noting that ‘hidden employment’ as a terminology appeared in the work of Fieldhouse and Hollywood (47) where they used the term ‘hidden’ to uncover ex-miners who were unemployed but removed from the official unemployment register as retirees or being classed permanently sick. Beatty and Fothergill (48) picked up on the terminology when they discovered socially determined of disability benefit recipients that overlapped spatial patterns of closed mines. The people in areas of low employment opportunities in 1970s chose to receive disability benefit instead of unemployment insurance. The authors argue that as a result of the UK’s fiscal austerity measures and welfare reform, a significant number of individuals with poor health or disabilities have been pushed towards unemployment benefits rather than disability benefits. In many cases, these individuals have been completely excluded from the benefits system (48).

### 5.3 Older jobseekers

**Figure 6:**
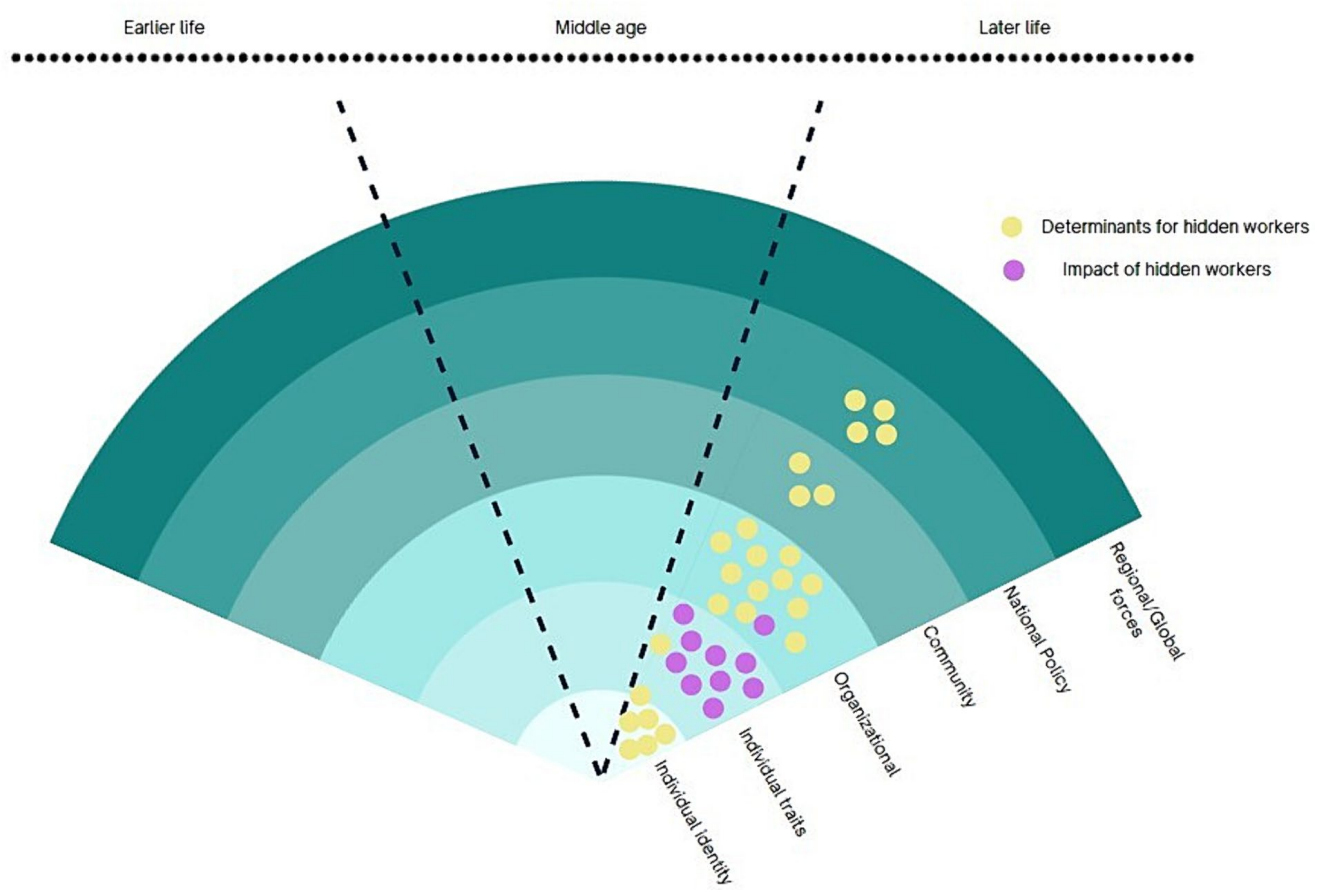
Older jobseekers research cluster.

This research tradition, shown in Figure 6, has its disciplinary ground on career science and occupational psychiatry. The job loss is not studied in isolation but within a process which begins with job search, recruitment process, job prospectus and interacts with recruitment agency attitudes, firm’s hiring policy (49). One of the tradition’s most prominent work is Jahoda’s book (1982), *Employment and Unemployment Social-Psychological Analysis*. The work demonstrates the potential of this emerging field of social-psychology and its application on unemployment to better understand individual’s experience with work. The scholars in this tradition are interested in the factors that influence older jobseekers’ job search attitudes and behaviour as well as firms’ intention to recruit and keep the older workers. There is a disciplinary overlap with welfare state cluster in terms of identifying key policies that impacts older jobseekers, such hiring policies, or employment support schemes.

Individual decision making or experience of unemployment are pronounced in this research tradition as well as highlighting sub-group differences in their experience taking into account various identities and health conditions (50). They are also interested in identifying how different groups of individuals make their retirement pathways (51). There have been number of policy evaluations in terms of shaping individual decisions.

This cluster has actively delved into understanding how the old age predict the job search behaviour of older individuals (52, 53) as well as divers support or interventions (54, 55). Age discrimination is another important topic in this cluster. Farber and colleagues (56) examines the role of age in the employer’s decision to hire applicants. In Abrams et al. (57) study, they highlight a prevailing stereotype that deems older individuals as hireable only if they are explicitly cast in a subordinate role to candidates with a younger age profile. In their study, Brough et al. (58) examine the social perceptions surrounding the cognitive performance and job attitudes of older workers in comparison to their younger counterparts.

### 5.4 Life course perspective

**Figure 7:**
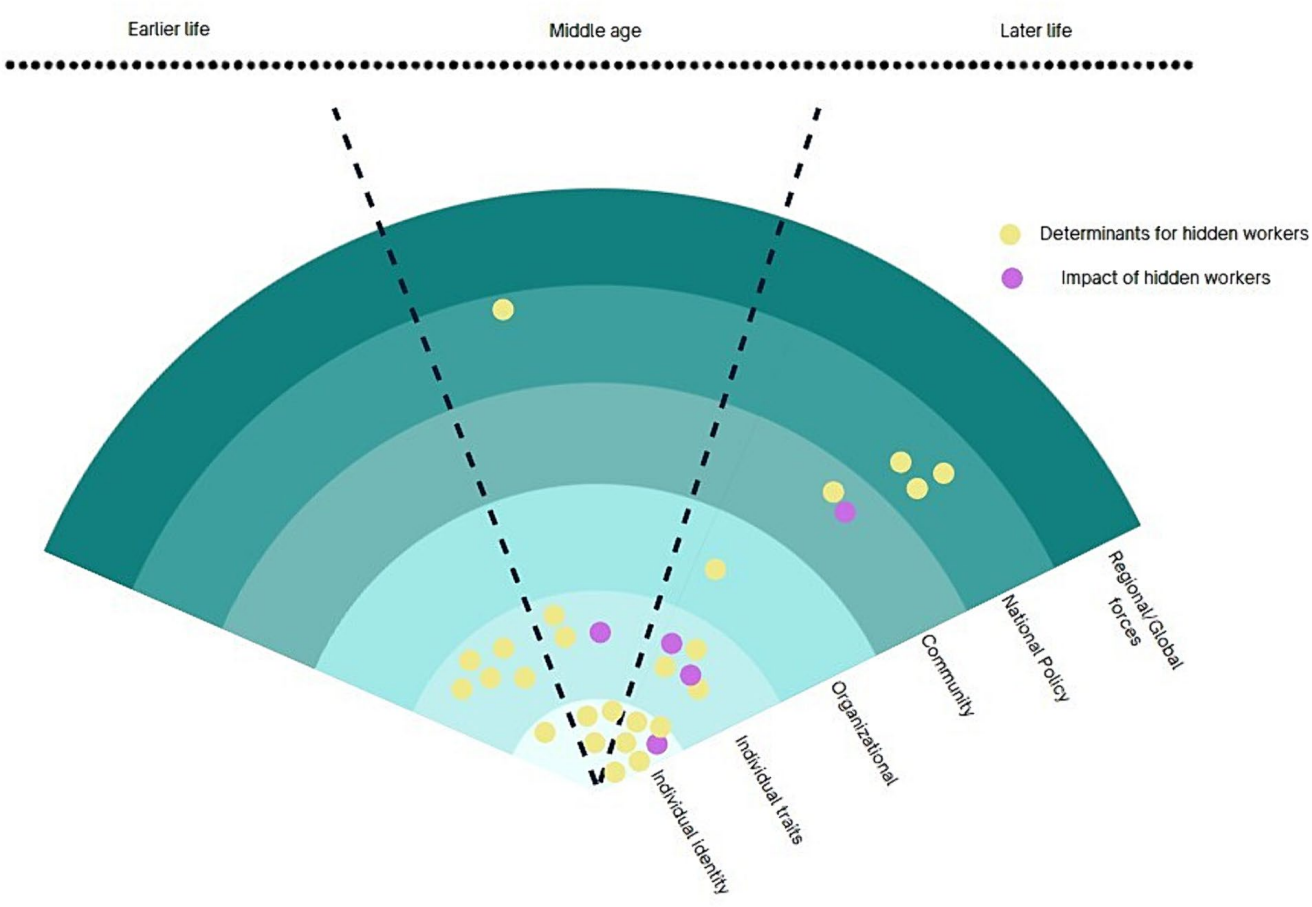
Life course research cluster.

Life course perspective studies multilevel, multidimensional, linked, and unfolding effect of earlier life traits and characteristics on later life trajectories (59). As the life course perspective identifies broad life determinants at all stages, this cluster report on broad variables such as gender, race, health conditions as well as individual traits including upbringing condition, substance use, parenthood experience in relation to their links to later labour market participation, as shown in Figure 7.

Parenthood and childbearing responsibilities have been found to be associated with lower workforce participation among working age women (60, 61). In Australia, a study suggests that women who have caring responsibilities during adulthood face greater challenges in establishing a consistent career compared to men, potentially due to cultural expectations and prevailing gender roles (62). The patterns of reproduction of inequalities were studied manifested at multiple levels as barriers to employment for marginalised older individuals in forms of ageism at workplace, community, and lack of choices in later work life (63–65). Brand (66) work investigates widespread spillover effect of unemployment. The ‘scarring effect’ of unemployment was one of the prominent topic in this tradition to understand the lingering impact of later-life trajectories (67, 68).

### 5.5 Retirement transitions

**Figure 8:**
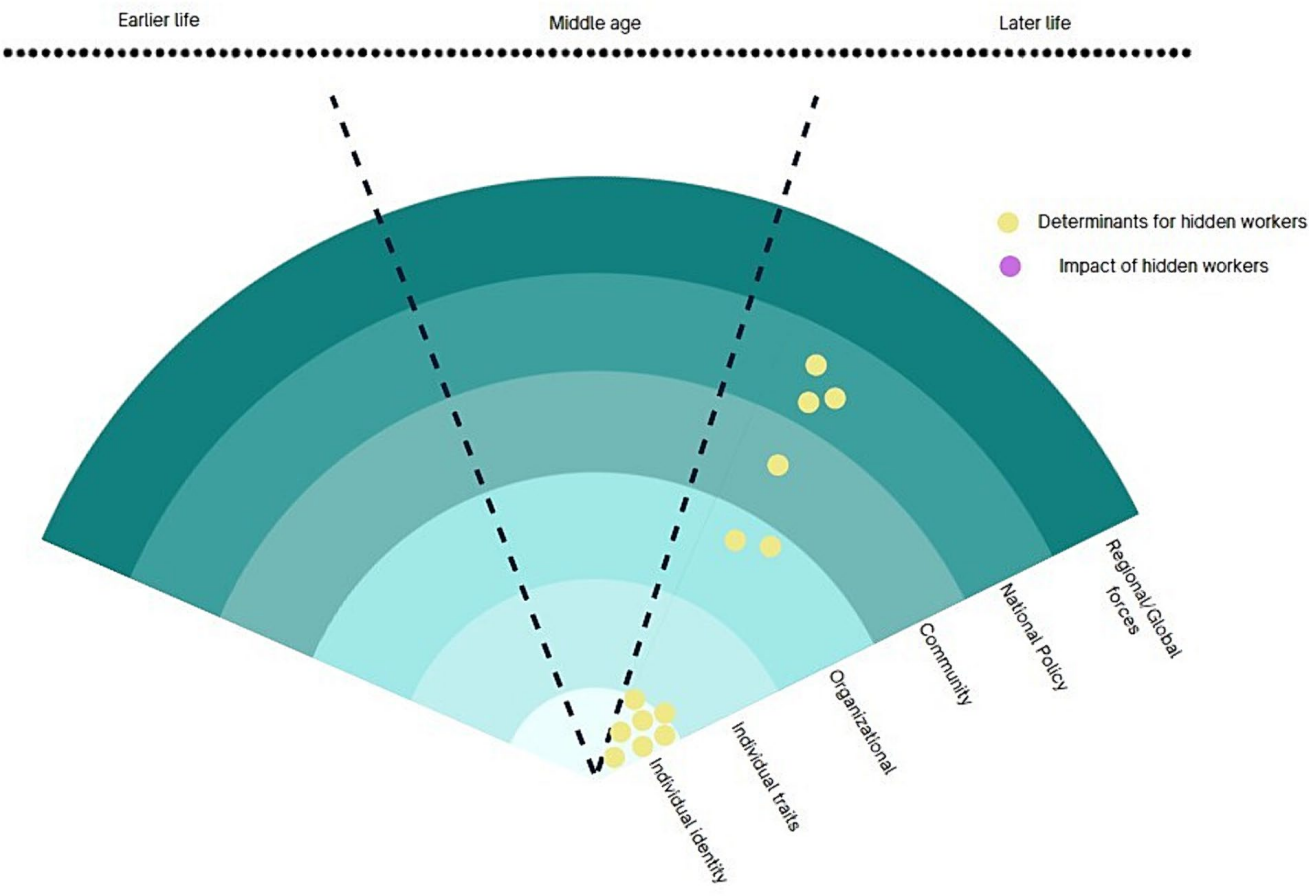
Retirement research cluster.

This research cluster is multidisciplinary with a mix of psychology, gerontology and business & economics. This research tradition, shown in Figure 8, moves away from the simple definition of retirement as ‘an exit from full-time work into full-time leisure’ (69) and accepts the dynamic patterns of retirement transitions (including entrepreneurship, bridge employment, phased retirement, early retirement, partial retirement, postretirement employment and job loss). Employability, intention to work, expectations, attitudes to diverse retirement decisions were prevalent topics in this tradition. The cluster includes studies that are concerned with business and management decision making on recruitment and retention of older workers which has an overlap with the older jobseekers tradition.

The level of analysis is mostly on individual retirement decisions, while some are on organisational commitment or new firm-level alternative options to early retirement (70, 71). While the level of analysis is at individual, the nature of research questions is more granular: what contributes to the retirement decision or the uptake of bridge employment (72, 73). The loss of a job is not merely viewed as a momentary occurrence, but rather as a life-long stratification process (73–75). The studies in this group introduces new subgroups of later working lives, including senior entrepreneurs, discouraged older workers and people who experienced involuntary job loss which are less studied groups within the research clusters of hidden workers (76, 77).

## 6 Discussion

The impact of being ‘hidden workers’ ranges from individual to socioeconomic/public health burden across the research traditions. Health indicators are most common variables to identify the impact of being hidden workers, usually framed as the cost of being unemployed. While mental/physical health are frequently employed variables for individual health impact, life course perspective goes further, suggesting intergenerational impact. Mental health, mortality, suicide were common indicators for public health burden. Welfare expenditure or social security benefit were also widely employed socioeconomic burden of unemployment. Across the five research clusters, elements of both constructs can be found, although some traditions lean more towards one construct than the other.

This review aims to draw attention to the challenges faced by researchers focusing on aging and offer a framework for overcoming these obstacles. Additionally, the framework used here can be employed to identify ways to design and evaluate pathways in specific intervention setting or specific target population, reducing the transdisciplinary gap between published research and practice. For instance, it may help conceptualise inner and outer settings variables and impact timeframes that may affect community-based intervention for hidden workers with specific illness or injury. Limitation in contemplating these components may impact intervention for their population and implementation strategy and resources.

The life course cluster is particularly helpful in this respect. Socioeconomic constraints often limit workforce participation and social determinants of health or life course perspective is increasingly becoming important to implement equity-focused intervention. Yet, there have not been comprehensive framework to identify challenges at a broader socioecological and chronosystemic manner. By taking account hidden workers’ connections to various levels and vulnerabilities stemming from various sources can be an important contextual information for implementation science.

Hidden workers indeed the group with multiple identities; a transitioning retiree, still looking for bridge work, caring for some, who may also need to be cared and ethnic minority all at the same time, which necessitates the transdisciplinary knowledge formation for effective interventions. This study informs us that while diverse clusters have approached the topic with their disciplinary approach, the central themes of hidden workers have been unemployment, social exclusion and vulnerability. Future transdisciplinary collaborative research can be based on these overarching topics to promote health and wellbeing of hidden workers.

Current research landscape of hidden workers in aging populations shows majority of works being done in public, health and labour economics disciplines, forming two main research clusters, namely public health and welfare state research traditions. The older jobseekers and retirement transition traditions are emerging clusters with more interdisciplinary grounds to respond to the wicked problem of hidden workers. As a first TDR informed research using meta-narrative analysis with a clear intention to comprehend not only different research paradigms but policy responses from diverse research clusters, this work can potentially be used for transdisciplinary policy agenda-setting for hidden workers by facilitating multi-actor dialogue.

Cross-sectoral approaches are widely recognised as more effective in identifying and addressing complex problems, such as hidden workers. By fostering collaboration across sectors, these approaches yield better actions and outcomes. The transdisciplinary connections of discourses from various research traditions identified here are important resources for government agencies to approach the issue as one holistic problem. This study provides valuable insights and potentially inspire other cross-sectoral approaches.

## 7 Limitations

The limitations of this review stem from the methodological characteristics of meta-narrative reviews. While the decisions were carefully made to achieve transdisciplinary understanding of hidden workers for implementation researchers, there are number of important issues that could have improved the study’s rigour. This review focuses on the main research clusters in hidden workers in aging population, potentially missing sub-streams within the identified clusters.

Additionally, as identified previously in the methodology section, the use of a document co-citation network inherently limits the inclusion of more recent studies that may not yet been cited widely. It is worth noting that recent work in the post-pandemic world is asking why the hidden workers persist amidst the increasing job opportunities and points to systemic discrimination in hiring process coupled with the technological divide (3). There have also been an ongoing research in organisational practices and interventions that support successful aging at work For instance, job carving and crafting has emerged in organisational psychology literature as a means to increase inclusivity and creativity for various population subgroup (78–80). By increasing autonomy and by removing language or requirements that may exclude or discriminate older population or other subgroups and attract hidden talents (81).

The study also lacks a broad geographic representation, with most publications coming from North America, Europe, and Australia. Despite the attempt to overcome this with allowing the works with citations from other communities to count, the limitation still persists. Authors have carried out more detailed scientometric study on hidden workers submitted elsewhere which reports that the periodic concentration of the research clusters by countries. While the publications from United States includes studies from one to three decades ago, countries in South America and Africa shows more recent publications. In short, the network analysis reveals that African nations are emerging research networks in this research topic. However, they were not included in this meta narrative analysis due to relatively low co-citation strength of papers. These limitations should be considered when interpreting the findings.

This paper has not delved deeply into the spectrum of issues related to hidden workers among older population. The challenges of older job seekers and those in transient retirement group have not been sufficiently covered in this study. This limitation has fuelled the researchers to embark on a 2-year mixed method study of *Hidden Workers in Aging Australia* to uncover the diverse population group and their challenges through the intersectionality lens.

Furthermore, age and ageing were mostly described as a limiting aspect or as a barrier in the older population’s pathway to labour market. However, not all the research clusters have identified age as a barrier. Older jobseekers cluster and the retirement pathway cluster have also focused on the benefits of the mature age workers. The publication of ageism has been a dominant theme whereas the positive aspects of aging consisted only small portion of the literature. While it is imperative to remove the barrier for the hidden workers, it is just as much important to accumulate the knowledge on positive aspect of getting older.

It is important to note that the five clusters we have identified in this review are not the only research clusters on this topic, nor do they encompass diverse stereotypes associated with hidden workers in the aging population. Instead, we present them as an initial framework to facilitate a better comprehension of the transdisciplinary conceptual approaches to hidden workers.

## 8 Conclusion

This paper offers an exploration of key different research traditions of hidden workers in aging population through a meta-narrative review. Our findings reveal a wide range of topics, scales, and approaches to studying hidden workers in the aging population. Through this review, we shed light on how researchers often overlook the underlying assumptions of their own research paradigm, which can limit the scope and impact of their work. It is crucial for researchers to recognise and embrace diverse worldviews in order to shape effective cross-sectoral interventions for transformative change. This study offers a promising avenue for emerging topic of TDR informed implementation science.

## Author contributions

SL: Conceptualization, Formal analysis, Funding acquisition, Investigation, Writing – original draft, Writing – review & editing. WK: Conceptualization, Data curation, Formal analysis, Investigation, Visualization, Writing – original draft, Writing – review & editing.

## References

[1] Roser M Ortiz-Ospina E Ritchie H. Life expectancy. Oxford, UK: Oxford Martin School (2013).

[2] Phillips (2023). Unemployment is coming down but not as much for older-workers. Available at: https://www.centernyc.org/urban-matters-2/unemployment-is-coming-down-but-not-as-much-for-older-workers (Accessed 23 December, 2023).

[3] Fuller JB Raman M Sage-Gavin E Hines K. Hidden workers: Untapped talent. Harvard: Harvard Business School Project on Managing the Future of Work and Accenture (2021).

[4] Baum S Bill A Mitchell WF. (2009). RETRACTED ARTICLE: Employability and Labour Under-utilization in Non-Metropolitan Labour Markets. Regional Studies, 43, 1091–1103.

[5] Prieto J Sehnbruch K Vidal D. (2022). A dynamic counting approach to measure multidimensional deprivations in jobs. Appl. Econ. Lett., 1–6.

[6] Australian Council of Social Service (ACOSS). (2022). Restoring full employment: policies for the Jobs and Skills Summit.

[7] Baker P Friel S Kay A Baum F Strazdins L Mackean T. What enables and constrains the inclusion of the social determinants of health inequities in government policy agendas? A narrative review. Int J Health Policy Manag. (2018) 7:101–11. doi: 10.15171/ijhpm.2017.130, PMID: 29524934 PMC5819370

[8] Onken L. Implementation science at the National Institute on Aging: the principles of it. Public Policy Aging Rep. (2022) 32:39–41. doi: 10.1093/ppar/prab034

[9] Organisation for Economic Co-operation and Development. Addressing societal challenges using transdisciplinary research OECD Publishing (2020).

[10] Trujillo CM Long TM. Document co-citation analysis to enhance transdisciplinary research. Sci Adv. (2018) 4:e1701130. doi: 10.1126/sciadv.1701130, PMID: 29308433 PMC5752411

[11] Greenhalgh T Robert G Macfarlane F Bate P Kyriakidou O Peacock R. Storylines of research in diffusion of innovation: a meta-narrative approach to systematic review. Soc Sci Med. (2005) 61:417–30. doi: 10.1016/j.socscimed.2004.12.001, PMID: 15893056

[12] Kim J de Leeuw E Harris-Roxas B Sainsbury P. Five urban health research traditions: a meta-narrative review. Soc Sci Med. (2023) 336:116265. doi: 10.1016/j.socscimed.2023.116265 37820495

[13] Chughtai S Blanchet K. Systems thinking in public health: a bibliographic contribution to a meta-narrative review. Health Policy Plan. (2017) 32:585–94. doi: 10.1093/heapol/czw159, PMID: 28062516

[14] MacLure K Stewart D Strath A. A systematic review of medical and nonmedical practitioners’ views of the impact of ehealth on shared care. Eur J Hosp Pharm. (2014) 21:54–62. doi: 10.1136/ejhpharm-2013-000337

[15] Masuda J Zupancic T Poland B Cole D. Environmental health and vulnerable populations in Canada: mapping an integrated equity-focused research agenda. Canadian Geographer-Geographe Canadien. (2008) 52:427–50. doi: 10.1111/j.1541-0064.2008.00223.x

[16] Small H. Paradigms, citations, and maps of science: a personal history. J Am Soc Inf Sci Technol. (2003) 54:394–9. doi: 10.1002/asi.10225

[17] Boyack KW Klavans R. Co-citation analysis, bibliographic coupling, and direct citation: which citation approach represents the research front most accurately? J Am Soc Inf Sci Technol. (2010) 61:2389–404. doi: 10.1002/asi.21419

[18] Greenhalgh T Wong G Westhorp G Pawson R. Protocol - realist and MetaNarrative evidence synthesis: evolving standards (RAMESES). BMC Med Res Methodol. (2011) 11:115. doi: 10.1186/1471-2288-11-115, PMID: 21843376 PMC3173389

[19] Eck N Waltman L. (2017). Citation-based clustering of publications using CitNetExplorer and VOSviewer. Scientometrics, 111. 10.1007/s11192-017-2300-7 PMC5400793 28490825

[20] Jin RL Shah CP Svoboda TJ. The impact of unemployment on health: a review of the evidence. CMAJ: Can Med Assoc J. (1995) 153:529–40. 7641151 PMC1487417

[21] Breuer C. Unemployment and suicide mortality: evidence from regional panel data in Europe. Health Econ. (2015) 24:936–50. doi: 10.1002/hec.3073, PMID: 24934277

[22] Córdoba-Doña JA San Sebastián M Escolar-Pujolar A Martínez-Faure JE Gustafsson PE. Economic crisis and suicidal behaviour: the role of unemployment, sex and age in Andalusia, southern Spain. Int J Equity Health. (2014) 13:1–10. doi: 10.1186/1475-9276-13-55 25062772 PMC4119181

[23] Kroll LE Lampert T. Changing health inequalities in Germany from 1994 to 2008 between employed and unemployed adults. Int J Public Health. (2011) 56:329–39. doi: 10.1007/s00038-011-0233-0, PMID: 21302130

[24] Mustard CA Bielecky A Etches J Wilkins R Tjepkema M Amick BC. Mortality following unemployment in Canada, 1991–2001. BMC Public Health. (2013) 13:1–10. doi: 10.1186/1471-2458-13-441 23642156 PMC3665659

[25] Levy I Cohen-Louck K. Predicting individual function during COVID-19 lockdown: depression, fear of COVID-19, age, and employment. Front Psychol. (2021) 12:682122. doi: 10.3389/fpsyg.2021.682122, PMID: 34276504 PMC8280345

[26] Matthay EC Duchowny KA Riley AR Galea S. Projected all-cause deaths attributable to COVID-19–related unemployment in the United States. Am J Public Health. (2021) 111:696–9. doi: 10.2105/AJPH.2020.306095, PMID: 33600244 PMC7958047

[27] Böckerman P Ilmakunnas P. Unemployment and self-assessed health: evidence from panel data. Health Econ. (2009) 18:161–79. doi: 10.1002/hec.1361, PMID: 18536002

[28] Claussen B Bjørndal A Hjort PF. Health and re-employment in a two year follow up of long term unemployed. J Epidemiol Community Health. (1993) 47:14–8. doi: 10.1136/jech.47.1.14, PMID: 8436885 PMC1059702

[29] Virtanen P Janlert U Hammarström A. Health status and health behaviour as predictors of the occurrence of unemployment and prolonged unemployment. Public Health. (2013) 127:46–52. doi: 10.1016/j.puhe.2012.10.016, PMID: 23158056

[30] Disney R Emmerson C Wakefield M. Ill health and retirement in Britain: a panel data-based analysis. J Health Econ. (2006) 25:621–49. doi: 10.1016/j.jhealeco.2005.05.004, PMID: 16678924

[31] Haardt D. Transitions out of and Back to employment among older men and women in the UK. UK: ISER (2006).

[32] Little A. Inactivity and labour market attachment in Britain. Scottish Journal of Political Economy. (2007) 54:19–54. doi: 10.1111/j.1467-9485.2007.00403.x

[33] Arrighi HM Hertz-Picciotto I. The evolving concept of the healthy worker survivor effect. Epidemiology. (1994) 5:189–96. doi: 10.1097/00001648-199403000-00009, PMID: 8172994

[34] Arrighi HM Hertz-Picciotto I. Definitions, sources, magnitude, effect modifiers, and strategies of reduction of the healthy worker effect. J Occup Med. (1993) 35:890–1. doi: 10.1097/00043764-199309000-00009, PMID: 8229339

[35] Östlin P. The 'health-related selection effect' on occupational morbidity rates. Scand J Soc Med. (1989) 17:265–70. doi: 10.1177/140349488901700402, PMID: 2602917

[36] Siebert U Rothenbacher D Daniel U Brenner H. Demonstration of the healthy worker survivor effect in a cohort of workers in the construction industry. Occup Environ Med. (2001) 58:774–9. doi: 10.1136/oem.58.12.774, PMID: 11706143 PMC1740083

[37] Kordovski VM Woods SP Verduzco M Beltran J. The effects of aging and HIV disease on employment status and functioning. Rehabil Psychol. (2017) 62:591–9. doi: 10.1037/rep0000175, PMID: 29265874 PMC5744895

[38] Castro-Marrero J Faro M Zaragozá MC Aliste L De Sevilla TF Alegre J. Unemployment and work disability in individuals with chronic fatigue syndrome/myalgic encephalomyelitis: a community-based cross-sectional study from Spain. BMC Public Health. (2019) 19:1–13. doi: 10.1186/s12889-019-7225-z 31253111 PMC6599355

[39] Baumberg B. Fit-for-work–or work fit for disabled people? 1 the role of changing job demands and control in incapacity claims. J Soc Policy. (2014) 43:289–310. doi: 10.1017/S0047279413000810

[40] Béland D Myles J. Varieties of federalism, institutional legacies, and social policy: comparing old-age and unemployment insurance reform in Canada. Int J Soc Welf. (2012) 21:S75–87. doi: 10.1111/j.1468-2397.2011.00838.x

[41] Engels B Geyer J Haan P. Pension incentives and early retirement. Labour Econ. (2017) 47:216–31. doi: 10.1016/j.labeco.2017.05.006

[42] Lammers M Bloemen H Hochguertel S. Job search requirements for older unemployed: transitions to employment, early retirement and disability benefits. Eur Econ Rev. (2013) 58:31–57. doi: 10.1016/j.euroecorev.2012.11.003

[43] Reichlin P. Economic stagnation and recession: the difficult Italian transition to the monetary union. J Mod Ital Stud. (2019) 24:402–14. doi: 10.1080/1354571X.2019.1605717

[44] Støver M Pape K Johnsen R Fleten N Sund ER Claussen B. Unemployment and disability pension-an 18-year follow-up study of a 40-year-old population in a Norwegian county. BMC Public Health. (2012) 12:1–8. doi: 10.1186/1471-2458-12-148 22369630 PMC3305666

[45] Hakola T Uusitalo R. Not so voluntary retirement decisions? Evidence from a pension reform. J Public Econ. (2005) 89:2121–36. doi: 10.1016/j.jpubeco.2004.12.001

[46] Anxo D Ericson T Jolivet A. Working longer in European countries: underestimated and unexpected effects. Int J Manpow. (2012) 33:612–28. doi: 10.1108/01437721211261787

[47] Fieldhouse E Hollywood E. Life after mining: hidden unemployment and changing patterns of economic activity amongst miners in England and Wales, 1981–1991. Work Employ Soc. (1999) 13:483–502.

[48] Beatty C Fothergill S. The diversion from ‘unemployment’to ‘sickness’ across British regions and districts. Reg Stud. (2005) 39:837–54. doi: 10.1080/00343400500289804

[49] Bonoli G Hinrichs K. Statistical discrimination and employers' recruitment: practices for low-skilled workers. Eur Soc. (2012) 14:338–61. doi: 10.1080/14616696.2012.677050

[50] Wanberg CR. The individual experience of unemployment. Annu Rev Psychol. (2012) 63:369–96. doi: 10.1146/annurev-psych-120710-100500 21721936

[51] Wang M Zhan Y Liu S Shultz KS. Antecedents of bridge employment: a longitudinal investigation. J Appl Psychol. (2008) 93:818–30. doi: 10.1037/0021-9010.93.4.818, PMID: 18642986

[52] Kanfer R Wanberg CR Kantrowitz TM. Job search and employment: a personality–motivational analysis and meta-analytic review. J Appl Psychol. (2001) 86:837–55. doi: 10.1037/0021-9010.86.5.837, PMID: 11596801

[53] Zacher H. Older job seekers' job search intensity: the interplay of proactive personality, age and occupational future time perspective. Ageing Soc. (2013) 33:1139–66. doi: 10.1017/S0144686X12000451

[54] Beard TR Ford GS Saba RP Seals RA Jr. Internet use and job search. Telecommun Policy. (2012) 36:260–73. doi: 10.1016/j.telpol.2011.12.001

[55] Rife JC Belcher JR. Assisting unemployed older workers to become reemployed: an experimental evaluation. Res Soc Work Pract. (1994) 4:3–13. doi: 10.1177/104973159400400101

[56] Farber HS Herbst CM Silverman D Von Wachter T. (2019). Whom do employers want? The role of recent employment and unemployment status and age. J. Labor Econ. 37, 323–349.

[57] Abrams D Swift HJ Drury L. Old and unemployable? How age-based stereotypes affect willingness to hire job candidates. J Soc Issues. (2016) 72:105–21. doi: 10.1111/josi.12158, PMID: 27635102 PMC4999032

[58] Brough P Johnson G Drummond S Pennisi S Timms C. Comparisons of cognitive ability and job attitudes of older and younger workers. Equality, Diversity and Inclusion: Int J. (2011) 30:105–26. doi: 10.1108/02610151111116508

[59] Brüderl J Kratz F Bauer G. Life course research with panel data: an analysis of the reproduction of social inequality. Adv Life Course Res. (2019) 41:100247. doi: 10.1016/j.alcr.2018.09.003, PMID: 36738044

[60] Baranowska-Rataj A Matysiak A. The causal effects of the number of children on female employment-do European institutional and gender conditions matter? J Lab Res. (2016) 37:343–67. doi: 10.1007/s12122-016-9231-6

[61] Wahrendorf M Zaninotto P Hoven H Head J Carr E. Late life employment histories and their association with work and family formation during adulthood: a sequence analysis based on ELSA. J Gerontol: Series B. (2018) 73:1263–77. doi: 10.1093/geronb/gbx066, PMID: 28575487 PMC6146763

[62] Majeed A Abbasi MK Hameed S Imran A Rahim N. Isolation and characterization of plant growth-promoting rhizobacteria from wheat rhizosphere and their effect on plant growth promotion. Front Microbiol. (2015) 6:132438. doi: 10.3389/fmicb.2015.00198 PMC4362341 25852661

[63] Hammarström A Gustafsson PE Strandh M Virtanen P Janlert U. It’s no surprise! Men are not hit more than women by the health consequences of unemployment in the northern Swedish cohort. Scand J Public Health. (2011) 39:187–93. doi: 10.1177/1403494810394906 21382857

[64] Puig-Barrachina V Malmusi D Martínez JM Benach J. Monitoring social determinants of health inequalities: the impact of unemployment among vulnerable groups. Int J Health Serv. (2011) 41:459–82. doi: 10.2190/HS.41.3.d, PMID: 21842573

[65] Reine I Novo M Hammarström A. Unemployment and ill health–a gender analysis: results from a 14-year follow-up of the northern Swedish cohort. Public Health. (2013) 127:214–22. doi: 10.1016/j.puhe.2012.12.005, PMID: 23375366

[66] Brand JE. The far-reaching impact of job loss and unemployment. Annu Rev Sociol. (2015) 41:359–75. doi: 10.1146/annurev-soc-071913-043237, PMID: 26336327 PMC4553243

[67] Daly M Delaney L. The scarring effect of unemployment throughout adulthood on psychological distress at age 50: estimates controlling for early adulthood distress and childhood psychological factors. Soc Sci Med. (2013) 80:19–23. doi: 10.1016/j.socscimed.2012.12.008, PMID: 23415587

[68] Dewilde C. Lifecourse determinants and incomes in retirement: Belgium and the United Kingdom compared. Ageing Soc. (2012) 32:587–615. doi: 10.1017/S0144686X11000407

[69] Sullivan SE Al Ariss A. Making sense of different perspectives on career transitions: a review and agenda for future research. Hum Resour Manag Rev. (2021) 31:100727–17. doi: 10.1016/j.hrmr.2019.100727

[70] Jones DA McIntosh BR. Organizational and occupational commitment in relation to bridge employment and retirement intentions. J Vocat Behav. (2010) 77:290–303. doi: 10.1016/j.jvb.2010.04.004

[71] Saba T Guerin G. Extending employment beyond retirement age: the case of health care managers in Quebec. Public Personnel Manag. (2005) 34:195–214. doi: 10.1177/009102600503400205

[72] Feldman DC. The decision to retire early: a review and conceptualization. Acad Manag Rev. (1994) 19:285–311. doi: 10.2307/258706

[73] Noonan AE. “At this point now”: older workers' reflections on their current employment experiences. Int J Aging Hum Dev. (2005) 61:211–41. doi: 10.2190/38CX-C90V-0K37-VLJA, PMID: 16248291

[74] Cahill KE Giandrea MD Quinn JF. Retirement patterns from career employment. The Gerontologist. (2006) 46:514–23. doi: 10.1093/geront/46.4.514 16921005

[75] Ulrich LB Brott PE. Older workers and bridge employment: redefining retirement. J Employ Couns. (2005) 42:159–70. doi: 10.1002/j.2161-1920.2005.tb01087.x

[76] Chan S Huff Stevens A. Job loss and employment patterns of older workers. J Labor Econ. (2001) 19:484–521. doi: 10.1086/319568

[77] Ranzijn R Carson E Winefield AH Price D. On the scrap-heap at 45: the human impact of mature-aged unemployment. J Occup Organ Psychol. (2006) 79:467–79. doi: 10.1348/096317905X66828

[78] Condon C. Enein-Donovan L. Gilmore M. Jordan M. (2004). When existing jobs don’t fit: a guide to job creation. The institute brief. Issue 17. Institute for Community Inclusion. Available at: https://www.communityinclusion.org/pdf/ib17.pdf (download 27 may 2019).

[79] European Commission. Promising PES practice - job-carving for jobseekers with disabilities. Luxembourg: Publications Office of the European Union (2018).

[80] Wrzesniewski A Dutton JE. Crafting a job: Revisioning employees as active crafters of their work. Acad Manag Rev. (2001) 26:179–201. doi: 10.2307/259118

[81] Wong CM Tetrick LE. Job crafting: older workers’ mechanism for maintaining person-job fit. Front Psychol. (2017) 8:277313. doi: 10.3389/fpsyg.2017.01548 PMC5596060 28943859

