# Supplementary material for: A meta-narrative review of research traditions on hidden workers in aging population for transdisciplinary implementation research
Source: Front Public Health. 2024 Jun 26;12:1415770. doi: 10.3389/fpubh.2024.1415770 (PMC11238214; doi:10.3389/fpubh.2024.1415770)
Supplement: Supplementary file 1 [file Table_1.DOCX]

# Supplement 1. Search terms

## Table 1 Search terms

| **Concept** | **Search terms** |
| --- | --- |
| Older | old OR older OR aged OR elder* OR vulnerable OR "over 45" OR "over 50" OR “mature” |
| Hidden Worker | NEAR/3  (jobseeker* OR unemploy* OR "part time" OR underemploy* OR "under employ*" OR "seek* work*" OR "bridge employ*" OR “discourage*” OR “hidden” OR “invisible”) |
