# Supplementary material for: A meta-narrative review of research traditions on hidden workers in aging population for transdisciplinary implementation research
Source: Front Public Health. 2024 Jun 26;12:1415770. doi: 10.3389/fpubh.2024.1415770 (PMC11238214; doi:10.3389/fpubh.2024.1415770)
Supplement: Supplementary file 2 [file Table_2.DOCX]

Supplement 2. Summary of publications (Total = 197 publications)

Public health approach to Unemployment (N=63)

| Research group | Author, year | Title | *Source* | [Field](http://ulrichsweb.serialssolutions.com/) | Geographical region (First author affiliation) | Type of study | Focus of paper | Main topic |
| --- | --- | --- | --- | --- | --- | --- | --- | --- |
| 1 | Levy, I and Cohen-Louck, K. 2021 | Predicting Individual Function During COVID-19 Lockdown: Depression, Fear of COVID-19, Age, and Employment | *Frontiers in Psychology* | Psychology | Israel | Empirical | victimization paradox | Age and employment were negatively associated with depression and economic fears related to COVID-19. Despite more frequent COVID-19 related health complications among older adults, young & unemployed are more at risk. |
| 2 | Matthay, EC; Duchowny, KA; (...); Galea, S, 2021 | Projected All-Cause Deaths Attributable to COVID-19-Related Unemployment in the United States | *American Journal of Public Health* | Public Health | USA | Empirical | COVID-19 Mortality and unemployment | Based on the unemployment-mortality association, unemployment data aged 25 to 64 years, Deaths attributable to COVID-19-related unemployment will add to those directly associated with the virus and will disproportionately burden groups already experiencing incommensurate COVID-19 mortality. |
| 3 | Castro-Marrero, J; Faro, M; (...); Alegre, J, 2019 | Unemployment and work disability in individuals with chronic fatigue syndrome/myalgic encephalomyelitis: a community-based cross-sectional study from Spain | *BMC Public Health* | Public, Environmental & Occupational Health | Spain | Empirical | determinants of work disability | Unemployment is consistently associated with an increased risk of work disability due to CFS/ME, although further more rigorous research is now needed to help in targeting interventions at the workplace. |
| 4 | Morrisroe, K; Sudararajan, V; (...); Nikpour, M, 2018 | Work productivity in systemic sclerosis, its economic burden and association with health-related quality of life | *Rheumatology* | Rheumatology | Australia | Empirical | Work productivity, unemployment and economic burden of SSc | SSc is associated with considerable unemployment and reduced productivity, which in turn is associated with a substantial economic burden and poor HRQoL. Raising awareness and identifying modifiable factors are possible ways of reducing this burden. |
| 5 | Kordovski, VM; Woods, SP; (...); Beltran, J, 2017 | The Effects of Aging and HIV Disease on Employment Status and Functioning | *Rehabilitation Psychology* | Psychology | USA | Empirical | HIV and older age on employment status | Findings suggest that older age and HIV disease have additive adverse effects on employment status, but not work functioning, and that employment status is associated with both neurocognitive and medical risk factors among older HIV+ adults. Further longitudinal research is needed to elucidate specific disease and demographic characteristics that may operate as protective factors for retaining gainful employment among older HIV+ adults. |
| 6 | Corbiere, M; Lecomte, T; (...); Goldner, EM, 2017 | Predictors of Acquisition of Competitive Employment for People Enrolled in Supported Employment Programs | *Journal of Nervous and Mental Disease* | Neurosciences & NeurologyPsychiatry | Canada | Program evaluation | Mental illness | A total of 489 persons with a severe mental illness and 97 employment specialists working in 24 SE programs across three Canadian provinces were included in the study. Overall, 43% of the sample obtained competitive work. Both client variables and employment specialist competencies, while controlling for the quality of SE programs implementation, predicted job acquisition. Multilevel analyses further indicated that younger client age, shorter duration of unemployment, and client use of job search strategies, as well as the working alliance perceived by the employment specialist, were the strongest predictors of competitive employment for people with severe mental illness, with 51% of variance explained. For people with severe mental illness seeking employment, active job search behaviors, relational abilities, and employment specialist competencies are central contributors to acquisition of competitive employment. |
| 7 | Kerr, WC; Kaplan, MS; (...); McFarland, BH, 2017 | Economic Recession, Alcohol, and Suicide Rates: Comparative Effects of Poverty, Foreclosure, and Job Loss | *American Journal of Preventive Medicine* | Public, Environmental & Occupational HealthGeneral & Internal Medicine | USA | Empirical | suicide | Population risk of suicide was most clearly associated with county-level poverty rates, indicating that programs addressing area poverty should be targeted for reducing suicide risk. Poverty rates were also associated with increased alcohol involvement for men aged 45-64 years, indicating a role for alcohol in suicide for this working-aged group. However, negative associations between economic indicators and alcohol involvement were found for four groups, suggesting that non-economic factors or more general economic effects not captured by these indicators may have played a larger role in alcohol-related suicide increases. |
| 8 | Bertin, P; Fagnani, F; (...); Kobelt, G, 2016 | Impact of rheumatoid arthritis on career progression, productivity, and employability: The PRET Study | *Joint Bone Spine* | Rheumatology | France | retrospective cross-sectional study | Work disability | Of 488 surveyed patients, 364(74.6%) were actively employed, 31(6.4%) were job seekers, and 93 (19.1%) had left the workforce. In the employed group, mean age was 48.9 years; 82.1% of patients were women; mean RA duration was 11.6 years; and the HAQ score correlated strongly with various markers for decreased productivity including sick leaves, temporary or permanent work discontinuation, and having unwillingly downgraded from a full-time to a part-time work schedule or changed to a different job. Among job seekers, 54% had lost their previous job because of their RA.  Conclusion: RA is associated with various forms of work disability, which are directly related to the severity of disease-related functional impairments. |
| 9 | Breuer, C, 2015 | Unemployment and Suicide Mortality: Evidence from Regional Panel Data in Europe | *Health Economics* | Business & Economics Health Care Sciences & Services | Germany | Longitudinal study | Suicide | his paper addresses the influence of economic activity on suicide mortality in Europe. To this end, it employs a new panel data set of 275 regions in 29 countries over the period 1999-2010. The results suggest that unemployment does have a significantly positive influence on suicides. In line with economic theory, this influence varies among gender and age groups. Men of working age are particularly sensitive, while old-age suicide mortality (older than 65years old) hardly responds to unemployment. Moreover, real economic growth negatively affects the suicide rates of working-age men. The results withstand several robustness checks, such as sample variations, and after controlling for serial and spatial autocorrelation. |
| 10 | Colell, E; Sánchez-Niubò, A; (...); Domingo-Salvany, A, 2015 | Economic crisis and changes in drug use in the Spanish economically active population | *Addiction* | Substance Abuse Psychiatry | Spain | Empirical | Alcohol | During a period of economic recession in Spain, heavy drinking decreased and binge drinking increased. Sporadic cannabis use increased among older unemployed men and women. Heavy use of hypnotics/sedatives increased among employed men while older women increased use irrespective of employment status. |
| 11 | Bouwmans, C; de Sonneville, C; (...); Hakkaart-van Roijen, L, 2015 | Employment and the associated impact on quality of life in people diagnosed with schizophrenia | *Neuropsychiatric Disease and Treatment* | Neurosciences & Neurology Psychiatry | Netherlands | Empirical | schizophrenia | The most frequently reported factors associated with employment were negative and cognitive symptoms, age of onset, and duration and course of the disease. Individual characteristics associated with unemployment were older age, lower education, and sex (female). Additionally, environmental factors, eg, the availability of welfare benefits and vocational support programs, seemed to play a role. Generally, being employed was positively associated with HRQoL. However, the causal direction of this association remained unclear, as studies on the bidirectional relationship between employment and HRQoL were lacking. |
| 12 | Tillett, W; Shaddick, G; (...); McHugh, N, 2015 | Factors influencing work disability in psoriatic arthritis: first results from a large UK multicentre study | *Rheumatology* | Rheumatology | England | Empirical | Work disability | Reduced effectiveness at work was associated with measures of disease activity, whereas unemployment, considered the endpoint of WD, was associated with employer factors, age and disease duration. A longitudinal study is under way to determine whether treatment to reduce disease activity ameliorates WD in the real-world setting. |
| 13 | Roelen, CAM; Heymans, MW; (...); van Rhenen, W, 2014 | Work Ability Index as Tool to Identify Workers at Risk of Premature Work Exit | *Journal of Occupational Rehabilitation* | Rehabilitation Social Issues | Netherlands | Empirical | Work ability index (WAI) | To investigate the Work Ability Index (WAI) as tool for identifying workers at risk of premature work exit in terms of disability pension, unemployment, or early retirement. The discriminative ability decreased with age from AUC = 0.78 in workers aged 30-39 years to AUC = 0.69 in workers a parts per thousand yen50 years of age. Discrimination failed for unemployment (AUC = 0.51; 95 % CI 0.47-0.55) and early retirement (AUC = 0.58; 95 % CI 0.53-0.61). Conclusions The WAI can be used to identify construction workers < 50 years of age at increased risk of disability pension and invite them for preventive interventions. |
| 14 | Gabbe, BJ; Sleney, JS; (...); Christie, N, 2014 | Financial and employment impacts of serious injury: A qualitative study | *Injury-International Journal of the Care of the Injured* | General & Internal Medicine Emergency Medicine Orthopedics  Surgery | Australia | Empirical | Injury and return to work | Seriously injured patients commonly experienced substantial financial and work-related impacts of injury. Participants of working age who were unemployed prior to injury, did not have extensive leave accrual at their pre-injury employment, and those not covered by injury compensation schemes or income protection insurance clearly represent participants "at risk'' for substantial financial hardship post-injury. Early identification of these patients, and improved provision of information about financial support services, budgeting and work retraining could assist in alleviating financial stress after injury |
| 15 | Córdoba-Doña, JA; San Sebastián, M; (...); Gustafsson, PE, 2014 | Economic crisis and suicidal behaviour: the role of unemployment, sex and age in Andalusia, Southern Spain | *International Journal for Equity in Health* | Public, Environmental & Occupational Health archive | Spain | Empirical | Suicide | This study enhances our understanding of the potential effects of the economic crisis on the rapidly increasing suicide attempt rates in women and men, and the association of unemployment with growing suicidal behaviour in men. Research on the suicide effects of the economic crisis may need to take into account earlier stages of the suicidal process, and that this effect may differ by age and sex. |
| 16 | Worach-Kardas, H and Kostrzewski, S, 2014 | Quality of Life and Health State of Long - Term Unemployed in Older Production Age | *Applied Research in Quality of Life* | Social Sciences - Other Topics | Poland | Empirical | Mental health | The findings of the analysis indicate that unemployment entails many negative health consequences and the long-term stress connected with being out of work leads to the decline in the quality of life and worsening of mental state. The multidimensional effects of unemployment depend not only on the economic situation of the particular household, but also on perceived health status, personal relationships and the sense of ability to work. |
| 17 | Lundin, A; Falkstedt, D; (...); Hemmingsson, T, 2014 | Unemployment and coronary heart disease among middle-aged men in Sweden: 39 243 men followed for 8 years | *Occupational and Environmental Medicine* | Public, Environmental & Occupational Health | Sweden | Empirical | CHD risk | Unemployment was associated with increased risk of CHD after adjustment for confounders. We interpret the increased risk of CHD associated with unemployment as potentially the somatic result of a process started by stress. |
| 18 | Helvik, AS; Krokstad, S and Tambs, K, 2013 | Hearing loss and risk of early retirement. The HUNT study | *European Journal of Public Health* | Public, Environmental & Occupational Health | Norway | Cross sectional study | Hearing loss & early retirement | The risk of early retirement increased with degree of loss of low-frequency hearing in young and middle-aged men and middle-aged women. The middle-aged men and women experiencing hearing disability had an increased risk of early retirement. Degree of hearing level was not associated with part-time work, but in middle-aged men, awareness of having a hearing loss was associated with part-time employment. Degree of low-frequency hearing loss was associated with early retirement but not with part-time work. Perceived hearing disability increased the risk of early retirement in middle-aged men and women and also the risk of part-time work in middle-aged men. |
| 19 | Evans-Lacko, S; Knapp, M; (...); Mojtabai, R, 2013 | The Mental Health Consequences of the Recession: Economic Hardship and Employment of People with Mental Health Problems in 27 European Countries | *Plos One* | Science & Technology - Other Topics | England | Empirical | Mental health | These findings study suggest that times of economic hardship may intensify social exclusion of people with mental health problems, especially males and individuals with lower education. Interventions to combat economic exclusion and to promote social participation of individuals with mental health problems are even more important during times of economic crisis, and these efforts should target support to the most vulnerable groups. |
| 20 | Mustard, CA; Bielecky, A; (...); Aronson, KJ, 2013 | Mortality following unemployment in Canada, 1991-2001 | *BMC Public Health* | Public, Environmental & Occupational Health | Canada | Cohort study | Mortality risk | Consistent with results reported from other long-duration cohort studies, unemployed men and women in this cohort had an elevated risk of mortality for accidents and violence, as well as for chronic diseases. The persistence of elevated mortality risks over two consecutive multi-year periods suggests that exposure to unemployment in 1991 may have marked persons at risk of cumulative socioeconomic hardship. |
| 21 | Kjær, T; Boje, CR; (...); Dalton, SO, 2013 | Affiliation to the work market after curative treatment of head-and-neck cancer: A population-based study from the DAHANCA database | *Acta Oncologica* | Oncology | Denmark | Cohort study | Survivors | Short education [ odds ratio (OR) 4.8; 95% confidence interval (CI) 2.2-10.4], low income (OR 3.2; 95% CI 1.8-5.8), living alone (OR 3.0; 95% CI 2.1-4.4) and having a Charlson comorbidity index score of 3 or more (OR 5.9; 95% CI 3.1-11) were significantly associated with early retirement overall and in all site groups. For the subgroup of patients who were employed before diagnosis, the risk pattern was similar. Tumour stage was not associated with early retirement or unemployment. Conclusions. Cancer-related factors were less strongly associated with early retirement and unemployment than socioeconomic factors and comorbidity. Clinicians treating HNSCC patients should be aware of the socioeconomic factors related to work market affiliation in order to provide more intensive social support or targeted rehabilitation for this patient group. |
| 22 | Virtanen, P; Janlert, U and Hammarström, A, 2013 | Health status and health behaviour as predictors of the occurrence of unemployment and prolonged unemployment | *Public Health* | Public, Environmental & Occupational Health | Finland | Cohort study | Health selection | There is health-related selection into unemployment in early middle age, irrespective of unemployment earlier in the life course. High risk ratios for prolonged unemployment suggest that selection takes place, in particular, at re-employment. The findings indicate the need for policies to prevent those with a history of health problems being at a disadvantage in terms of future employment. |
| 23 | Dewilde, C, 2012 | Lifecourse determinants and incomes in retirement: Belgium and the United Kingdom compared | *Ageing & Society* | Geriatrics & Gerontology | Netherlands | Longitudinal study | 'scarring effect' of unemployment | In this paper, the impact of lifecourse family and labour market experiences on household incomes of older people in Belgium and the United Kingdom (UK) is analysed. To this end, panel data and life-history information from the Panel Study of Belgian Households and the British Household Panel Survey are combined. The results show that old-age income is indeed influenced by previous lifecourse experiences, and that differences between Belgium and the UK can be explained in terms of (the development over time of) welfare regime arrangements. Family experiences have a larger impact on old-age incomes in 'male-breadwinner' Belgium, while in Britain labour market events are more important. As social transfers in Britain are more aimed at poverty prevention and less at income replacement, a 'scarring effect' of unemployment persists even into old age. Also, the more of one's career is spent in blue-collar work or self-employment/farming, the lower the income in old age. A new finding is that, notwithstanding the high level of 'de-commodification' achieved by the Belgian welfare state, this effect turns out to be significantly stronger in Belgium than in the UK. Compared to the market, the welfare state is hence a more efficient 'mechanism' of stratification for incomes in old age. |
| 24 | Stover, M; Pape, K; (...); Bjorngaard, JH, 2012 | Unemployment and disability pension-an 18-year follow-up study of a 40-year-old population in a Norwegian county | *BMC Public Health* | Public, Environmental & Occupational Health | Norway | Cohort study | Disability pension | This study explored the association of unemployment and an increased risk of receiving disability pension, and the possibility that this risk is attributed to municipality-specific characteristics. Becoming unemployed increased the risk of receiving subsequent disability pension. However, adjusting for baseline health status, health behaviour and education attenuated this impact considerably. The multilevel analysis indicated that a minor, yet statistically significant, proportion of the risk of disability pension can be attributed to the municipality of residence. |
| 25 | Freyer-Adam, J; Gaertner, B; (...); John, U, 2011 | Health risk factors and self-rated health among job-seekers | *BMC Public Health* | Public, Environmental & Occupational Health | Germany | Empirical | Health risk | prevention efforts to reduce health risk factors and to increase health among job-seekers are needed, and job agencies appear a feasible setting for their implementation. |
| 26 | Walid, MS; Robinson, ECM and Robinson, JS, 2011 | Higher comorbidity rates in unemployed patients may significantly impact the cost of spine surgery | *Journal of Clinical Neuroscience* | Neurosciences & Neurology | USA | Empirical | Comorbidities | Multivariate analysis showed that a history of coronary artery bypass/stent procedure, chronic renal disease or preoperative opioid use had a significant impact on length of stay and hospital charges in unemployed spine surgery patients. Thus, unemployment in spinal surgery candidates is associated with higher comorbidity rates with a significant impact on healthcare cost. More research is needed into the relationship between unemployment and consumption of healthcare resources |
| 27 | Kroll, LE and Lampert, T, 2011 | Unemployment, Social Support and Health Problems Results of the GEDA Study in Germany, 2009 | *Deutsches Arzteblatt International* | General & Internal Medicine | Germany | Cross sectional study | Health impact of unemployment | It is well documented that the unemployed have more health problems than the employed, and that social support facilitates coping with unemployment. The association of unemployment and social support with health was examined on the basis of representative data derived from a German study. Unemployed persons aged 30 to 59 years suffer physical, emotional, and functional impairment more commonly than employed persons. Men and women with little social support are more likely to be impaired in these three areas whether they are employed or not. Regression analysis reveals that unemployment and social support have significant, independent effects on both the incidence of such impairments (ORunemployed = 1.2-1.7, ORsupported = 0.4-0.9) and on their duration (IRRunemployed = 1.3-1.8, IRRsupported = 0.6-0.8) after age, income, and education have been controlled for. |
| 28 | Stuckler, D; Basu, S; (...); McKee, M, 2009 | The public health effect of economic crises and alternative policy responses in Europe: an empirical analysis | *Lancet* | General & Internal Medicine | England | Longitudinal study | active labour market programmes | We noted that every 1% increase in unemployment was associated with a 0.79% rise in suicides at ages younger than 65 years (95% CI 0.16-1.42; 60-550 potential excess deaths [mean 310] EU-wide), although the effect size was non-significant at all ages (0.49%, -0.04 to 1.02), and with a 0.79% rise in homicides (95% CI 0.06-1.52; 3-80 potential excess deaths [mean 40] EU-wide). By contrast, road-traffic deaths decreased by 1.39% (0.64-2.14; 290-980 potential fewer deaths [mean 630] EU-wide). A more than 3% increase in unemployment had a greater effect on suicides at ages younger than 65 years (4.45%, 95% CI 0.65-8.24; 250-3220 potential excess deaths [mean 1740] EU-wide) and deaths from alcohol abuse (28.0%,12.30-43.70; 1550-5490 potential excess deaths [mean 3500] EU-wide). We noted no consistent evidence across the EU that all-cause mortality rates increased when unemployment rose, although populations varied substantially in how sensitive mortality was to economic crises, depending partly on differences in social protection. Every US$10 per person increased investment in active labour market programmes reduced the effect of unemployment on suicides by 0.038% (95% CI -0.004 to -0.071).  Interpretation Rises in unemployment are associated with significant short-term increases in premature deaths from intentional violence, while reducing traffic fatalities. Active labour market programmes that keep and reintegrate workers in jobs could mitigate some adverse health effects of economic downturns. |
| 29 | Kirchhoff, AC; Leisenring, W; (...); Wickizer, T, 2010 | Unemployment Among Adult Survivors of Childhood Cancer A Report From the Childhood Cancer Survivor Study | *Medical Care* | Health Care Sciences & Services Public, Environmental & Occupational Health | USA | Cohort study | Cancer survivorship | Adult childhood cancer survivors report high levels of unemployment, although it is unknown whether this is because of health or employability limitations. Unemployed survivors reported higher levels of poor physical functioning than employed survivors, and had lower education and income and were more likely to be publicly insured than unemployed siblings. |
| 30 | Zimmerman, M; Galione, JN; (...); Ruggero, CJ, 2010 | Sustained unemployment in psychiatric outpatients with bipolar disorder: frequency and association with demographic variables and comorbid disorders | *Bipolar Disorders* | Neurosciences & Neurology Psychiatry | USA | Mixed method study | Comorbidity | Most patients presenting for the treatment of bipolar disorder have missed some time from work due to psychiatric reasons, and the persistence of employment problems is considerable. Comorbid psychiatric disorders are a potentially treatable risk factor for sustained unemployment. It is therefore of public health significance to determine if current treatments are effective in bipolar disorder patients with current panic disorder, and if not, to attempt to develop treatments that are effective. |
| 31 | Jang, SN; Cho, SI; (...); Berkman, LF, 2009 | Employment Status and Depressive Symptoms in Koreans: Results From a Baseline Survey of the Korean Longitudinal Study of Aging | *Journals of Gerontology Series B- Psychological Sciences and Social Sciences* | Geriatrics & Gerontology Psychology | USA | Cross sectional study | Health impact of unemployment | Employment was associated with fewer depressive symptoms among middle-aged men but not among older men. Unemployment was associated with more depressive symptoms among middle-aged women but not among older women. We discuss the increased depressive symptoms among older employed men and the differential association of employment status with age and gender in the context of Korean social structure. |
| 32 | Liwowsky, I; Kramer, D; (...); Hegerl, U, 2009 | Screening for depression in the older long-term unemployed | *Social Psychiatry and Psychiatric Epidemiology* | Psychiatry | Germany | Structured Clinical Interview. | Depression | Screening with the WHO-5 within unemployment offices is useful to detect people with depressive disorders. Many of them do not receive optimal treatment and can be motivated to seek professional help. Reducing depression by better treatment will also increase the chances of reemployment. |
| 33 | Kristen, AV; Ammon, K; (...); Dengler, TJ, 2009 | Return to Work After Heart Transplantation: Discrepancy With Subjective Work Ability | *Transplantation* | Immunology Surgery Transplantation | Germany | Cohort study | Survivor | The rate of employment after HTX in Germany is significantly lower than the subjective perception of the individual ability to work; underscoring the importance of sociodemographic and psychologic aspects during rehabilitation of HTX recipients. |
| 34 | Fergusson, DM; Boden, JM and Horwood, LJ, 2007 | Unemployment and suicidal behavior in a New Zealand birth cohort - A fixed effects regression analysis | *CRISIS- The Journal of Crisis Intervention and Suicide Prevention* | Psychiatry Psychology | New Zealand | Longitudinal study | Suicide | associations between unemployment and suicidal ideation reduced to marginal significance (p < . 10), while the association between unemployment and suicide attempts was not statistically significant (p >. 10). After adjustment, those experiencing 6 or more months of unemployment in a given year had odds of suicidal ideation that were 1.43 (95% CI: .96 to 2.16) times higher, and rates of suicide attempts that were 1.72 (95% CI: .89 to 3.32) times higher, than those who were not exposed to unemployment. Although unemployment was associated with moderate increases in risks of suicidal behaviors, much of this association was explained by confounding factors. |
| 35 | Roelfs, DJ; Shor, E; (...); Schwartz, JE, 2011 | Losing life and livelihood: A systematic review and meta-analysis of unemployment and all-cause mortality | *Social Science & Medicine* | Public, Environmental & Occupational Health Biomedical Social Sciences | USA | Meta analysis | Mortality | The study is a random effects meta-analysis and meta-regression designed to assess the association between unemployment and all-cause mortality among working-age persons. We extracted 235 mortality risk estimates from 42 studies, providing data on more than 20 million persons. The mean hazard ratio (HR) for mortality was 1.63 among HRs adjusted for age and additional covariates. The mean effect was higher for men than for women. Unemployment was associated with an increased mortality risk for those in their early and middle careers, but less for those in their late career. The risk of death was highest during the first 10 years of follow-up, but decreased subsequently. The mean HR was 24% lower among the subset of studies controlling for health-related behaviors. Public health initiatives could target unemployed persons for more aggressive cardiovascular screening and interventions aimed at reducing risk-taking behaviors. |
| 36 | Schrag, A and Banks, P, 2006 | Time of loss of employment in Parkinson's disease | *Movement Disorders* | Neurosciences & Neurology | England | survival analysis | Parkinson's disease | Parkinson's disease leads to loss of employment on average within less than 10 years of disease onset. However, the variability of time to loss of employment is large, indicating that other factors than onset age and disease duration influence loss of patients' employment. |
| 37 | Stankunas, M; Kalediene, R; (...); Kapustinskiene, V, 2006 | Duration of unemployment and depression: a cross-sectional survey in Lithuania | *BMC Public Health* | Public, Environmental & Occupational Health | Lithuania | Cross sectional study | mental health | The results indicated that depression is a severe problem in the unemployed population. Depression is more elevated among the long-term unemployed. This leads to arguing for common efforts in providing needed social support and health care to reduce the effects of unemployment on mental health. |
| 38 | de Boer, AGEM; Verbeek, JHAM and van Dijk, FJH, 2006 | Adult survivors of childhood cancer and unemployment - A metaanalysis | *Cancer* | Oncology | Finland | Meta analysis | Cancer survivors | lower education or intelligence quotient, female gender, motor impairment or epilepsy, and radiotherapy. Adult survivors of childhood cancer are at risk of unemployment, especially the subgroup of survivors of CNS and brain tumors. Interventions to enhance participation in work life should be developed and evaluated. |
| 39 | Lindström, M, 2005 | Psychosocial work conditions, unemployment and self-reported psychological health:: a population-based study | *Occupational Medicine-Oxford* | Public, Environmental & Occupational Health | Sweden | Cross sectional study | Psychological health | The study found that certain psychosocial work factors are associated with higher levels of self-reported psychological ill-health and illustrates the great importance of psychosocial conditions in determining psychological health at the population level. As found elsewhere, being unemployed was an even stronger predictor of psychological ill-health. |
| 40 | Feagan, BG; Bala, M; (...); Hanauer, S, 2005 | Unemployment and disability in patients with moderately to severely active Crohn's disease | *Journal of Clinical Gastroenterology* | Gastroenterology & Hepatology | Canada | Cross sectional study | Quality of life | Patients with moderately to severely active Crohn's disease had low employment and high disability rates. Given their economic importance, assessment of these outcomes should be integrated into future evaluations of therapy, including clinical trials. |
| 41 | Artazcoz, L; Benach, J; (...); Cortès, I, 2004 | Unemployment and mental health:: Understanding the interactions among gender, family roles, and social class | *American Journal of Public Health* | Public, Environmental & Occupational Health | Spain | Cross sectional study | Depression, gender | Unemployment had more of an effect on the mental health of men than on that of women. Gender differences in effects were related to family responsibilities and social class. |
| 42 | Blakely, TA; Collings, SCD and Atkinson, J, 2003 | Unemployment and suicide. Evidence for a causal association? | *Journal of Epidemiology and Community Health* | Public, Environmental & Occupational Health | New Zealand | Cohort study | Suicide | Being unemployed was associated with a twofold to threefold increased relative risk of death by suicide, compared with being employed. About half of this association might be attributable to confounding by mental illness. |
| 43 | Price, R. H., Choi, J. N., & Vinokur, A. D., 2002 | Links in the chain of adversity following job loss: How financial strain and loss of personal control lead to depression, impaired functioning, and poor health. | *Journal of Occupational Psychology* | Psychology | USA | Longitudinal study | Depression | The authors tested hypotheses concerning risk mechanisms that follow involuntary job loss resulting in depression and the link between depression and poor health and functioning. A 2-year longitudinal study of 756 people experiencing job loss indicates that the critical mediating mechanisms in the chain of adversity from job loss to poor health and functioning are financial strain (FS) and a reduction in personal control (PC). FS mediates the relationship of job loss with depression and PC, whereas reduced PC mediates the adverse impacts of FS and depression on poor functioning and self-reports of poor health. Results suggest that loss of PC is a pathway through which economic adversity is transformed into chronic problems of poor health and impaired role and emotional functioning. |
| 44 | MacDonald, Z and Pudney, S, 2000 | Illicit drug use, unemployment, and occupational attainment | *Journal of Health Economics* | Business & Economics Health Care Sciences & Services | England | Cross sectional study | Substance use | In this paper, we use data from the British Crime Survey (BCS) to examine the effect of illicit drug use on labour market outcomes. We find very little evidence to support any relationship between drug use, hard or soft, and occupational attainment. However, we find compelling evidence to suggest that drug use, particularly the use of opiates, cocaine and crack cocaine, is associated with an increased risk of unemployment, regardless of age or gender |
| 45 | Liira, J and Leino-Arjas, P, 1999 | Predictors and consequences of unemployment in construction and forest work during a 5-year follow-up | *Scandinavian Journal of Work Environment & Health* | Public, Environmental & Occupational Health | Finland | Follow up survey | Occupational health risk | Unemployment among construction workers is to some extent dependent on life-style, health, and job satisfaction in addition to age, marital status, and unemployment history. For forest workers, unemployment is less determined by individual factors. Changes in distress and musculoskeletal symptoms are dependent on employment, particularly among construction workers. |
| 46 | Sander, AM; Kreutzer, JS and Fernandez, CC, 1997 | Neurobehavioral functioning, substance abuse, and employment after brain injury: Implications for vocational rehabilitation | *Journal of Head Trauma and Rehabilitation* | Neurosciences & Neurology Rehabilitation | USA | A quasi-experimental, fixed effects factorial design | Substance use | The unique neurobehavioral difficulties of unemployed persons should be carefully considered when developing empirically based preplacement training, job matching, and postplacement interventions. The high rate of alcohol use among employed persons indicates the need for follow-along that emphasizes education and prevention. |
| 47 | MORRELL, S; TAYLOR, R; (...); KERR, C, 1993 | SUICIDE AND UNEMPLOYMENT IN AUSTRALIA 1907-1990 | *Social Science and Medicine* | Public, Environmental & Occupational Health Biomedical Social Sciences | Australia | Time series analysis | Suicide | The association between suicide and unemployment for 15–24-year-old males was comparatively high for the recent period, 1966-1990. The increasingly youthful contribution to male suicide was demonstrated by a rise in the loss of life years during 1973-1984. Despite the inability of any investigation based on aggregate data to establish an unequivocable causal relationship, no evidence was detected to suggest that relatively high population levels of unemployment were not related to the occurrence of suicide. |
| 48 | CLAUSSEN, B; BJORNDAL, A and HJORT, PF, 1993 | HEALTH AND REEMPLOYMENT IN A 2 YEAR FOLLOW-UP OF LONG-TERM UNEMPLOYED | *Journal of Epidemiology and Community Health* | Public, Environmental & Occupational Health | Norway | Cross sectional study | Health selection | Health related selection to long term unemployment seems to explain a substantial part of the excess mental morbidity among unemployed people. An increased proportion of the long term unemployed will be vocationally handicapped as years pass, putting a heavy burden on social services. |
| 49 | Bartley, M, 1994 | UNEMPLOYMENT AND ILL HEALTH - UNDERSTANDING THE RELATIONSHIP | *Journal of Epidemiology and Community Health* | Public, Environmental & Occupational Health | England | Review | Mortality | To understand the relationship between unemployment and ill health and mortality, four mechanisms need to be considered: the role of relative poverty; social isolation and loss of self esteem; health related behaviour (including that associated with membership of certain types of ''subculture''); and the effect that a spell of unemployment has on subsequent employment patterns. |
| 50 | Böckerman, P and Ilmakunnas, P, 2009 | UNEMPLOYMENT AND SELF-ASSESSED HEALTH: EVIDENCE FROM PANEL DATA | *Health Economics* | Business & Economics Health Care Sciences & Services | Finland | Longitudinal study | Health selection | We examine the relationship between unemployment and self-assessed health using the European Community Household Panel for Finland over the period 1996-2001. Our results show that the event of becoming unemployed does not matter as such for self-assessed health. The health status of those that end up being unemployed is lower than that of the continually employed. Therefore, persons who have poor health arc being selected for the pool of the unemployed. This explains why. in a cross-section, unemployment is associated with poor self-assessed health. All in all, the cross-sectional negative relationship between unemployment and self-assessed health is not found longitudinally. |
| 51 | Dooley, D; Fielding, J and Levi, L, 1996 | Health and unemployment | *Annual Review of Public Health* | Public, Environmental & Occupational Health | USA | Review | Health selection | This paper reviews the relationship between health and inadequate employment, especially unemployment. Poor physical or mental health can lead, via poor work performance, to job loss; however, studies that control for such selection effects are still scarce except for a few health outcomes. For example, aggregate-level studies typically find a positive association between unemployment and suicide rates over time. At the individual level of analysis, panel surveys of laid-off workers tend to find increased psychiatric problems such as depression and substance abuse. Few studies have evaluated interventions to prevent or reduce the adverse health effects of job loss. There have been even fewer studies of the health effects of other types of inadequate employment such as the increasingly prevalent forms of underemployment. |
| 52 | Henkel, D, 2011 | Unemployment and substance use: a review of the literature (1990-2010) | *Current Drug Abuse Review* | Addiction & substance use | Germany | Review | Substance use | The current article summarizes the results of a comprehensive review of the international research published between 1990 and 2010. The research was focused on the prevalence of substance use/disorders among the unemployed and employed, the impact of substance abuse on unemployment and vice versa, the effect of unemployment on alcohol/ drug addiction treatment and smoking cessation, and the relationship between business cycle, unemployment rate and substance use. Over hundred-thirty relevant studies were identified investigating these issues. The main results are as follows: (1) Risky alcohol consumption (associated with hazardous, binge, and heavy drinking) is more prevalent among the unemployed. They are also more likely to be smokers, to use illicit and prescription drugs, and to have alcohol and drug disorders (abuse, dependence). (2) Problematic substance use increases the likelihood of unemployment and decreases the chance of finding and holding down a job. (3) Unemployment is a significant risk factor for substance use and the subsequent development of substance use disorders. However, the current research provides only limited information about which individuals are more likely to be affected. (4) Unemployment increases the risk of relapse after alcohol and drug addiction treatment. (5) The exact nature of the relationship between unemployment and the probability of smoking cessation remains unclear due to the mixed results observed in the literature review. (6) Drinking and smoking patterns appear to be procyclical. |
| 53 | Lewis, G and Sloggett, A, 1998 | Suicide, deprivation, and unemployment: record linkage study | *BMJ-British Medical Journal* | General & Internal Medicine | Wales | Longitudinal study | Suicide | The association between suicide and unemployment is more important than the association with other socioeconomic measures. Although some potentially important confounders were not adjusted for, the findings support the idea that unemployment or lack of job security increases the risk of suicide and that social and economic policies that reduce unemployment will also reduce the rate of suicide. |
| 54 | JIN, RL; SHAH, CP and SVOBODA, TJ, 1995 | THE IMPACT OF UNEMPLOYMENT ON HEALTH - A REVIEW OF THE EVIDENCE | *Canadian Medical Association Journal* | General & Internal Medicine | Canada | Review | mechanisms of causation | Evaluated on an epidemiologic basis, the evidence suggests a strong, positive association between unemployment and many adverse health outcomes. Whether unemployment causes these adverse outcomes is less straightforward however, because there are likely many mediating and confounding factors, which may be social, economic or clinical. Many authors have suggested mechanisms of causation, but further research is needed to test these hypotheses. |
| 55 | Martikainen, PT and Valkonen, T, 1996 | Excess mortality of unemployed men and women during a period of rapidly increasing unemployment | *Lancet* | General & Internal Medicine | Finland | Prospective study | Mortality | Individuals who experienced unemployment between 1987 and 1992 had greater mortality than those in employment after control for age, education, occupational class, and marital status. The mortality ratios for men and women unemployed for the first time in 1990, at a time of low national unemployment were 2.11 (95% CI 1.76-2.53) and 1.61 (1.09-2.36), respectively. These values were lower for those who were unemployed for the first time in 1992 when the national unemployment rate was very high (men 1.35 [1.16-1.56], women 1.30 [0.97-1.75]). The jobless who were re-employed had higher mortality than those who were continuously employed, but not as high as those who remained unemployed. |
| 56 | LINN, MW; SANDIFER, R and STEIN, S, 1985 | EFFECTS OF UNEMPLOYMENT ON MENTAL AND PHYSICAL HEALTH | *American Journal of Public Health* | Public, Environmental & Occupational Health | USA | prospective study | Mental and physical health impact | From a prospective study of the impact of stress on health in 300 men assessed every six months, men who became unemployed after entering the study were compared with an equal number, matched for age and race, who continued to work. Psychological and health data after unemployment were compared between the two groups by multivariate analysis of variance and covariance. After unemployment, symptoms of somatization, depression, and anxiety were significantly greater in the unemployed than employed. Large standard deviations on self-esteem scores in the unemployed group suggested that some men coped better than others with job-loss stress. Further analysis showed those with higher esteem had more support from family and friends than did those with low self-esteem. Furthermore, unemployed men made significantly more visits to their physicians, took more medications, and spent more days in bed sick than did employed individuals even though the number of diagnoses in the two groups were similar. |
| 57 | Lundin, A; Lundberg, I; (...); Hemmingsson, T, 2010 | Unemployment and mortality-a longitudinal prospective study on selection and causation in 49321 Swedish middle-aged men | *Journal of Epidemiology and Community Health* | Public, Environmental & Occupational Health | Sweden | Cohort study | Mortality | Unemployment is associated with increased risk of mortality. It is, however, not clear to what extent this is causal, or whether other risk factors remain uncontrolled for. The aim of this study was to investigate the association between unemployment and all-cause and cause-specific mortality, adjusting for indicators of mental disorder, behavioural risk factors and social factors over the life course. The results suggest that a substantial part of the increased relative risk of mortality associated with unemployment may be attributable to confounding by individual risk factors. |
| 58 | MORRIS, JK; COOK, DG and SHAPER, AG, 1994 | LOSS OF EMPLOYMENT AND MORTALITY | *BMJ-British Medical Journal* | General & Internal Medicine | England | Cohort study | Mortality | Men who experienced unemployment in the five years after initial screening were twice as likely to die during the following 5.5 years as men who remained continuously employed (relative risk 2.13 (95% confidence interval 1.71 to 2.65). After adjustment for socioeconomic variables (town and social class), health related behaviour (smoking, alcohol consumption, and body weight), and health indicators (recall of doctor diagnoses) that had been assessed at initial screening the relative risk was slightly reduced, to 1.95 (1.57 to 2.43). Even men who retired early for reasons other than illness and who appeared to be relatively advantaged and healthy had a significantly increased risk of mortality compared with men who remained continuously employed (relative risk 1.87 (1.35 to 2.60)). The increased risk of mortality from cancer was similar to that of mortality from cardiovascular disease (adjusted relative risk 2.07 and 2.13 respectively). |
| 59 | Milner, A; Page, A and LaMontagne, AD, 2013 | Long-Term Unemployment and Suicide: A Systematic Review and Meta-Analysis | *Plos One* | Science & Technology - Other Topics | Australia | A systematic review and meta-analysis | long-term unemployment | Findings suggest that long-term unemployment is associated with greater incidence of suicide. Results of the meta-analysis suggest that risk is greatest in the first five years, and persists at a lower but elevated level up to 16 years after unemployment. These findings are limited by the paucity of data on this topic. |
| 60 | Paul, KI and Moser, K, 2009 | Unemployment impairs mental health: Meta-analyses | *Journal of Vocational Behavior* | Psychology | Germany | Meta-analyses | Mental health | A significant difference was found for several indicator variables of mental health (mixed symptoms of distress, depression, anxiety, psychosomatic symptoms, subjective well-being, and self esteem). The average number of persons with psychological problems among the unemployed was 34%, compared to 16% among employed individuals. Moderator analyses demonstrated that men and people with blue-collar-jobs were more distressed by unemployment than women and people with white-collar jobs. Linear and curvilinear moderating effects of the duration of unemployment were also identified. Furthermore, the negative effect of unemployment on mental health was stronger in countries with a weak level of economic development, unequal income distributions, or weak unemployment protection systems compared to other countries. |
| 61 | MOSER, KA; GOLDBLATT, PO; (...); JONES, DR, 1987 | UNEMPLOYMENT AND MORTALITY - COMPARISON OF THE 1971 AND 1981 LONGITUDINAL-STUDY CENSUS SAMPLES | *British Medical Journal* | General & Internal Medicine | England | longitudinal study | Mortality | Mortality in the period 1981-3 among men in the Office of Population Censuses and Surveys longitudinal study who were seeking work in 1981 was examined to investigate whether the finding of a high mortality rate among a comparable group of men who were followed up from the 1971 Census was repeated despite appreciable changes in the size and structure of the labour force over the intervening years. The pattern of mortality shortly after both censuses suggests that sick unemployed men were not categorised as seeking work, and it is concluded that for both samples the mortality of those who were reported to be seeking work was raised for reasons other than initial poor health. |
| 62 | PLATT, S, 1984 | UNEMPLOYMENT AND SUICIDAL-BEHAVIOR - A REVIEW OF THE LITERATURE | *Social Science & Medicine* | Public, Environmental & Occupational Health Biomedical Social Sciences | Scotland | Review | Suicide | the author distinguishes between two categories of deliberately self-harmful act: those  with fatal outcome (suicide) and those with non-fatal outcome (parasuicide); and differentiates four major types pf quantitative research report: individual-cross-sectional; aggregate-cross-sectional; individuallongitudinal; and aggregate-longitudinal. Methodological issues and empirical research findings are  discussed separately for each type of study and each category of deliberate self-harm. |
| 63 | Ostamo, A; Lahelma, E and Lönnqvist, J, 2001 | Transitions of employment status among suicide attempters during a severe economic recession | *Social Science & Medicine* | Public, Environmental & Occupational Health Biomedical Social Sciences | Finland | Logistic regression analysis and survival analysis | Suicide | Unemployment rates among suicide attempters were higher than in the general population, male rates being higher than female rates throughout the recession. There were significant changes in the employment status of the entire attempted suicide population from 1989 to 1994, especially in terms of the transition from employment to unemployment. Gender, age and education level predicted exit from the labour market. Among suicide attempters young middle-aged men with low education had the highest risk of unemployment. |

Welfare state & aging workforce (N=58)

| **No.** | **Author, year** | **Title** | ***Source*** | [**Field**](http://ulrichsweb.serialssolutions.com/) | **Geographical region (First author affiliation)** | **Type of study** | **Focus of paper** | **Main topic** |
| --- | --- | --- | --- | --- | --- | --- | --- | --- |
| 1 | van Berkel, R, 2020 | Making welfare conditional: A street-level perspective | *Social Policy & Administration* | Housing and urban planning | Netherlands | Review | Welfare-to-work | Street-level studies show that the balance between disciplining and enabling aspects of welfare-to-work is most at risk for more vulnerable groups. |
| 2 | Reichlin, P, 2019 | Social Security and Old Age Unemployment Risk with Efficient Wage Bargaining | *Scandinavian Journal of Economics* | Business & Economics | Italy | Theoretical | Social security effects | Allocations with limited union membership are second-best inefficient as they generate too little labour supply in young age, too much consumption before retirement, too little employment of older workers (early retirement), and too little insurance against old age unemployment. Providing public transfers to early retirees (disability benefits or early pensions) might help to increase the degree of risk sharing at the cost of lower old age employment. |
| 3 | Farber, HS; Herbst, CM; (...); von Wachter, T, 2019 | Whom Do Employers Want? The Role of Recent Employment and Unemployment Status and Age | *Journal of Labour Economics* | Business & Economics | USA | résumé audit study | Labour market and job loss | We use a resume audit study to investigate the role of employment and unemployment histories in callbacks to job applications. We also find that both younger and older applicants have a lower callback probability than prime-aged applicants. |
| 4 | Axelrad, H; Malul, M and Luski, I, 2018 | Unemployment among younger and older individuals: does conventional data about unemployment tell us the whole story? | *Journal for Labour Market Research* | Business & Economics | USA | Empirical | Barrier for older workers | In this research we show that workers aged 30-44 were significantly more likely than those aged 45-59 to find a job a year after being unemployed. The main contribution is demonstrating empirically that since older workers' difficulties are related to their age, while for younger individuals the difficulties are more related to the business cycle, policy makers must devise different programs to address unemployment among young and older individuals. |
| 5 | Andersen, NA; Caswell, D and Larsen, F, 2017 | A New approach to Helping the hard-to-place Unemployed: The promise of developing new knowledge in an interactive and collaborative process | *European Journal of Social Security* | Public Administration | Denmark | Theoretical | Evidence-based knowledge generation | The reforms of the social and employment services that have swept across most of the developed world since the 1990s have enormously expanded the groups of citizens receiving active employment measures. Nevertheless, up until now, most countries have only seen limited results from enhancing the labour market participation of the most vulnerable groups. This model investigates the potential for integrated services and for co-production by acknowledging the importance of the experiences of frontline professionals and clients in developing employment services. |
| 6 | Engels, B; Geyer, J and Haan, P, 2017 | Pension incentives and early retirement | *Labour Economics* | Business & Economics | Germany | Empirical | Pension effect on retirement | We present evidence for sizeable labour market effects. In addition to direct effects on women older than 60 we find important anticipation effects before reaching the pension eligibility age. Overall we document that the pension reform leads to a postponement of retirement, an increase in employment and a shifting in unemployment over age rather than a substitution into unemployment. |
| 7 | Beatty, C and Fothergill, S, 2015 | Disability Benefits in an Age of Austerity | *Social Policy & Administration* | Development Studies Public Administration Social Issues Social Work | England | Empirical | Disability benefit effect and unemployment | This article takes a long-view of the huge rise in disability claimant numbers in the UK since the early 1980s and looks ahead to the trends that can now be expected to emerge in an era of fiscal austerity and welfare reform. The article's central thesis is that disability numbers are best understood as part of a triangular relationship between levels of employment, unemployment and sickness. In particular, the big decline of industrial employment in many places has often resulted in large-scale 'hidden unemployment' on disability benefits, especially among low-skilled workers. Looking ahead, the UK's welfare reforms are set to reduce disability claimant numbers but principally by restricting access to Employment and Support Allowance, the new disability benefit. The main effect will be to divert substantial numbers of men and women with ill health or disability onto unemployment benefits instead or, more often, out of the benefits system altogether. |
| 8 | Nikolova, M and Graham, C, 2014 | Employment, late-life work, retirement, and well-being in Europe and the United States | *IZA Journal of European Labor Studies* | Business & Economics | USA | Empirical | Retirement age | We find that voluntary part-time workers are happier, experience less stress and anger, and have higher job satisfaction than other employees. Using statistical matching, we show that late-life workers under voluntary part-time or full-time arrangements have higher well-being than retirees. There is no well-being premium for involuntary late-life work and self-employment compared to retirement, however. Our findings inform ongoing debates about the optimal retirement age and the fiscal burdens of public pension systems. |
| 9 | Baumberg, B. 2014 | Fit-for-Work - or Work Fit for Disabled People? The Role of Changing Job Demands and Control in Incapacity Claims | *Journal of Social Policy* | Public Administration Social Issues Social Work | England | Empirical | Disability Pension | The results show that people in low-control (but not high-demands) jobs are more likely to claim incapacity benefits in the following year, a result that is robust to a number of sensitivity analyses. Deteriorating job control seems to be a part of the explanation for rising incapacity, and strategies to cut the number of incapacity claimants should therefore consider ways to improve job control. Given the challenges in changing job characteristics, however, an equally important implication is that high levels of incapacity should not just be seen as a result of poor policies and a lack of jobs, but also as a result of the changing nature of work. |
| 10 | Chéron, A; Hairault, JO and Langot, F, 2013 | Life-Cycle Equilibrium Unemployment | *Journal of Labour Economics* | Business & Economics | France | Empirical | job creation--job destruction | This paper extends the job creation--job destruction approach to the labor market to take into account a deterministic finite horizon. As hirings and separations depend on the time over which investment costs can be recouped, the life-cycle setting implies age-differentiated labor-market flows. While search by the unemployed falls with age, the separation rate is rather U-shaped over the life cycle. Worker heterogeneity in the context of undirected search implies an intergenerational externality, which is not eliminated by the Hosios condition. We show that age-specific policies are required to attain the first-best allocation. |
| 11 | Koning, P and Raterink, M, 2013 | Re-employment Rates of Older Unemployed Workers: Decomposing the Effect of Birth Cohorts and Policy Changes | *Economist-Netherlands* | Business & Economics | Netherlands | Longitudinal study | potential benefit durations (PBD) | Using a Linear Probability Model, we decompose the effects of birth cohorts, age, calendar time and two policy measures that were targeted at older unemployed workers-i.e. increased job search obligations in 2004 and shorter potential benefit durations (PBD) in 2006. We find that policy effects predominantly explain the increased job return rates of unemployed of 55 years and older from 1999 to 2008. The introduction of search requirements has increased the one-year re-employment probability of eligible older men with about 5 % point, while the reduction in PBD has caused the one-year re-employment probability of eligible men to increase with 3 % point. |
| 12 | Lammers, M; Bloemen, H and Hochguertel, S, 2013 | Job search requirements for older unemployed: Transitions to employment, early retirement and disability benefits | *European Economic Review* | Business & Economics | Netherlands | Longitudinal study | sickness/disability benefits | We use a recent policy change in the Netherlands to study how changes in search requirements for the older unemployed affect their transition rates to employment, early retirement and sickness/disability benefits. The reform, becoming effective on January 1 2004, requires the elderly to formally report their job search efforts to the employment office in order to avoid a (temporary) cut in benefits. Before the new law was passed, unemployed individuals were allowed to stop all search activity at the moment they turned 57.5. Estimating various duration models using difference-in-difference and regression discontinuity approaches, we find that for several groups of individuals who are affected by the policy change, the stricter search requirements significantly increases their entry rate into employment. However, we also find evidence of a higher outflow to sickness/disability insurance schemes, a presumably unwanted side-effect of the policy change. |
| 13 | Béland, D and Myles, J, 2012 | Varieties of federalism, institutional legacies, and social policy: Comparing old-age and unemployment insurance reform in Canada | *International Journal of Social Welfare* | Social Work | Canada | Non-empirical study | unemployment insurance | Comparing old-age and unemployment insurance reform in Canada With reference to Canada, this article explores the politics of reform affecting two social insurance programs: Employment Insurance (EI) and the Canada Pension Plan (CPP). Comparing these two large, yet institutionally distinct, social insurance schemes underscores how institutional differences in policy legacies and governance among social programs create distinct obstacles and opportunities for reform in federal countries. Drawing on historical institutionalism and emphasizing the types of federalism and decision making specific to EI and CPP, the article explains key political differences between these two programs. Focusing on reforms enacted in the 1990s, the article explores the institutional obstacles and opportunities for policy change in EI and CPP, which offers insight into how these programs could change in the future. We show that different forms of federal governance pointing to the varieties of federalism and, more generally, the institutional and territorial logics embedded in these two programs create different obstacles and opportunities for reform. |
| 14 | Boockmann, B; Zwick, T; (...); Maier, M, 2012 | Do Hiring Subsidies Reduce Unemployment Among Older Workers? Evidence from Natural Experiment | *Journal of the European Economic Association* | Business & Economics | Germany | Natural experiment | Hiring subsidies | We estimate the effects of hiring subsidies for older workers on transitions from unemployment to employment in Germany. Using a natural experiment, our first set of estimates is based on a legal change extending the group of eligible unemployed persons. A subsequent legal change in the opposite direction is used to validate these results. Our data cover the population of unemployed jobseekers in Germany and was specifically made available for our purposes from administrative data. Consistent support for an employment effect of hiring subsidies can only be found for women in East Germany. Concerning other population groups, firms hiring behavior is hardly influenced by the program and hiring subsidies mainly lead to deadweight effects. |
| 15 | Dietz, M and Walwei, U, 2011 | Germany-No Country for Old Workers? | *Journal for Labour Market Research* | Business & Economics | Germany | Non-empirical | skill improvement, employability | German policy has focused on less early exits of older workers and far- reaching institutional changes concerning public pension schemes and unemployment benefit systems. A better overall development of the labor market and an increased labor force participation of women contributed to the more favorable employment situation of older workers. Nevertheless, we still observe severe problems for certain groups of older workers. The older workers are and the lower their skill level is the weaker is their labor market integration. If older persons are already unemployed they will experience severe difficulties to (re-)enter the labor market. In order to become actually a country for old workers the issue of employability needs much more attention. Such a strategy relies on continuous skill improvement as well as a healthy environment and does not only address older workers but also younger workers facing a prolonged working life. |
| 16 | Anxo, D; Ericson, T and Jolivet, A, 2012 | Working longer in European countries: underestimated and unexpected effects | *International Journal of Manpowers* | Business & Economics | Sweden | cross-country comparative study | Retirement age | Specific questions will be more acute with the effective postponement of retirement: increasing inequalities between groups of older workers, increasing uncertainty about the age of retirement, the way to keep lower educated workers in their jobs, sustainable working conditions, increasing risks of age discrimination, and impact of care of older relatives. |
| 17 | Lindsay, C and Houston, D, 2011 | Fit for purpose? Welfare reform and challenges for health and labour market policy in the UK | *Environment and Planning A* | Environmental Sciences & Ecology Geography | USA | Review | Sickness and incapacity benefit | This paper provides an extensive review of the most recent evidence to identify factors driving the rise in the number of people claiming IBs and, in light of this analysis, assesses whether current policy is fit for purpose. An important conclusion is that any national 'one-size fits all' supply-side policy response is blind to the distinctive geography of receipt of IBs and the complex combination of factors that leave some people trapped on these benefits. |
| 18 | Tatsiramos, K, 2010 | Job displacement and the transitions to re-employment and early retirement for non-employed older workers | *European Economic Review* | Business & Economics | Germany | Longitudinal study | work-retirement decision | Despite the relatively higher frequency of job displacement among older workers in Europe, little is known about its effect on the work-retirement decision. Employing individual data from the European Community Household Panel for a number of countries with differences in their institutional environments, the effect of job displacement for non-employed workers is identified separately for the transitions into re-employment and retirement. The findings suggest that in countries with relatively more generous unemployment insurance provisions for the older unemployed, which offer a pathway to early retirement, older displaced workers exhibit lower re-employment and higher retirement rates compared to the non-displaced. These results are robust to dynamic selection due to unobserved heterogeneity and to the endogeneity of displacement |
| 19 | Bambra, C and Smith, KE, 2010 | No longer deserving? Sickness benefit reform and the politics of (ill) health | *Critical Public Health* | Public, Environmental & Occupational Health Biomedical Social Sciences | England | Theoretical | Sickness and incapacity benefit | Income maintenance during unemployment, old age or long-term sickness is a key facet of welfare provision and an important mediatory factor in the relationship between socio-economic position and health status. Since October 2008, the main long-term sickness absence benefit in the UK (Incapacity Benefit) has been replaced by Employment Support Allowance. Despite the importance of income maintenance for health and health inequalities, this change has been largely ignored within public health circles. After outlining these reforms and providing a historical policy context, this article utilises welfare reform theory and empirical literature to argue that these changes represent a broader international transformation from welfare to workfare states, the re-emergence of labour discipline, and a political shift in how people suffering from ill health are categorised as disabled or not and perceived as 'deserving' or 'undeserving' of state support. Finally, the case is made for the need to develop a new critical public health research and practice agenda around worklessness. |
| 20 | Hairault, JO; Langot, F and Sopraseuth, T, 2010 | DISTANCE TO RETIREMENT AND OLDER WORKERS' EMPLOYMENT: THE CASE FOR DELAYING THE RETIREMENT AGE | *Journal of European Economic Association* | Business & Economics | France | Longitudinal study | Retirement decision | The time to retirement is then key to understanding older workers' employment. Countries with a retirement age of 60 are indeed characterized by lower employment rates for workers aged 55-59. Based on the French Labor Force Survey, we show that the likelihood of employment is significantly affected by the distance to retirement, in addition to age and other relevant variables. We then extend McCall's job search model by explicitly integrating life-cycle features with the retirement decision. Using simulations, we show that the distance effect in interaction with the generosity of unemployment benefits and the depressed demand for older workers explains the low rate of employment just before the eligibility age for the Social Security pension. Finally, we show that implementing actuarially fair schemes not only extends the retirement age, but also encourages a more intensive job search by older unemployed workers. |
| 21 | Koning, PWC and van Vuuren, DJ, 2010 | Disability insurance and unemployment insurance as substitute pathways | *Applied Economics* | Business & Economics | Netherlands | Longitudinal study | Disability insurance | In this article, we estimate the degree of substitution between enrolment into Disability Insurance (DI) and Unemployment Insurance (UI) in the Netherlands. Starting in the 1990s many policy measures aimed at reducing DI enrolment, and increase labour force participation. We quantify whether these policy measures have led to a reduction in hidden unemployment in DI. A side effect of the reforms may be increased pressure on UI. Therefore, we simultaneously estimate reverse substitution, that is, hidden disability in UI. To this end, we employ a sample of firms in the Dutch AVO database from the period 1993 to 2002. Using instrumental variables in a bivariate Tobit specification, we identify the hidden components in both respective schemes. The estimation results indicate that about 3% of all dismissals took place through DI, which implies that about one quarter of the DI enrolments observed in our sample in fact consists of hidden unemployment. We find no evidence for reverse substitution of disabled persons ending up in UI. |
| 22 | Holmqvist, M, 2009 | Medicalization of unemployment: individualizing social issues as personal problems in the Swedish welfare state | *Work, Employment and Society* | Business & Economics Sociology | Sweden | Qualitative study | Medicalisation of unemployment | This article reports qualitative data on how the Swedish Public Employment Service classifies unemployed individuals as 'occupationally disabled' in order to transfer them to various labour market programmes. The article draws on a framework of medicalization, arguing that the individualization of the social issue of unemployment into a personal trouble of disability is a neglected yet important phenomenon that has interesting implications for theory and policy. By classifying some people as disabled in order to explain their unemployment, medicalization can be seen as an important yet so far neglected mechanism in understanding how this individualizing enterprise comes about. It is concluded that by medicalizing unemployment, the target for society's intervention to fight the spectre of unemployment is primarily individuals' personal troubles rather than any social issues. |
| 23 | Sullivan, D and von Wachter, T, 2009 | JOB DISPLACEMENT AND MORTALITY: AN ANALYSIS USING ADMINISTRATIVE DATA | *Quarterly Journal of Economics* | Business & Economics | USA | Longitudinal study | Mortality | We use administrative data on the quarterly employment and earnings of Pennsylvanian workers in the 1970s and 1980s matched to Social Security Administration death records covering 1980-2006 to estimate the effects of job displacement on mortality. We find that for high-seniority male workers, mortality rates in the year after displacement are 50%-100% higher than would otherwise have been expected. The effect on mortality hazards declines sharply over time, but even twenty years after displacement, we estimate a 10%-15% increase in annual death hazards. If such increases were sustained indefinitely, they would imply a loss in life expectancy of 1.0-1.5 years for a worker displaced at age forty. We show that these results are not due to selective displacement of less healthy workers or to unstable industries or firms offering less healthy work environments. We also show that workers with larger losses in earnings tend to suffer greater increases in mortality. This correlation remains when we examine predicted earnings declines based on losses in industry, firm, or firm-size wage premiums. |
| 24 | McVittie, C; McKinlay, A and Widdicombe, S, 2008 | Passive and active non-employment: Age, employment and the identities of older non-working people | *Journal of Aging Studies* | Geriatrics & Gerontology | Scotland | Discourse analysis | Age discrimination | The declining participation of older people in the workforce has become a matter of concern in many parts of the world, including the European Union. Steps however taken to date to increase the participation of older people have proceeded on the basis of a limited understanding of the identities of these individuals and the consequences for employment. We report findings from a discourse analytic study of the understandings of 15 older non-employed individuals. Participants negotiated two different forms of identity, either drawing upon age discrimination or in minimising the relevance of employment to them. Both forms of identity orient to employment as a moral issue but each has different effects for the importance of finding employment and for the actions that are appropriate to the individual. Future efforts to promote greater participation of older people in the workforce need to acknowledge the various identities available to older nonemployed people. |
| 25 | Drever, AI and Hoffmeister, O, 2008 | Immigrants and social networks in a job-scarce environment: The case of Germany | *International Migration Review* | Demography | USA | Longitudinal study | Migrants | Though information about jobs passed through personal networks has been central to the labor market integration of immigrants in the United States, its role in the economic absorption of immigrants in Germany, where jobs are scarcer and employers more likely to demand formal qualifications, is less clear. Through analysis of German Socio-Economic Panel data, we discovered that nearly half of all immigrant-origin job changers found their positions through networks and that the most vulnerable to unemployment - the young and the less educated - were especially likely to rely on them. Also, jobs found through networks were as likely to lead to improved working conditions as jobs acquired through more formal means. These findings have implications both for debates about assimilation and for social policy. |
| 26 | Kantarci, T and Van Soest, A, 2008 | Gradual retirement: Preferences and limitations | *Economist-Netherlands* | Business & Economics | Netherlands | Review | Phased retirement | In the traditional retirement scenario, individuals work full-time or part-time until a given age, and then stop working abruptly. From the individual's point of view, it seems more attractive to have a smooth transition, with gradual retirement. In Sweden and other European countries, specific gradual retirement programs have been created in the past 20 years, first in combination with early retirement programs and later to increase labour market participation of older workers. This paper surveys the existing literature on gradual retirement in the US and Europe and analyzes the relevance of gradual retirement in the Netherlands as a tool to keep people employed longer. |
| 27 | Golden, L, 2008 | Limited Access: Disparities in Flexible Work Schedules and Work-at-home | *Journal of Family and Economic Issues* | Business & Economics Family Studies | USA | Empirical | Work-life balance | This research tests predictions regarding potential disparities among the employed by personal characteristics in the ability to vary the starting and ending times of their workday and engage in work from home. Women and African-Americans possess less access to flexible work schedules, even when controlling for most job characteristics. Married men have more access, but only if they are parents, and mothers only if they have pre-school-age children. Workers with part-time or long hours gain far greater access. Work-at-home is more common among women, the married and parents-thus, relatively more reflective of family demands. The results suggest where public and organizational policies could be focused to spread flexible work arrangements more toward those who both most value it and lack it. |
| 28 | Larsen, CA, 2008 | The political logic of labour market reforms and popular images of target groups | *Journal of European Social Policy* | Public Administration Social Issues | Denmark | Cross sectional study | Labour market reform | Even though the shift from 'passive' to 'active' labour market policy exhibits large cross-national variations, all examples seem to share two common characteristics: ( a) the first group exposed to the new policies and the group exposed to the harshest policies was young people on social assistance; and ( b) as the target group gradually came to include 'ordinary' unemployed people, most countries made exceptions for the oldest unemployed people. The article argues that this striking policy convergence has to do with the public perception of the target groups. The article substantiates this argument first, by giving a theoretical explanation for the different popular images of target groups, and second, by showing - using a national Australian sample - that these general popular images influence the way the public wants 'active' labour market policy to be conducted. |
| 29 | Coenders, M and Scheepers, P, 2008 | Changes in resistance to the social integration of foreigners in Germany 1980-2000: Individual and contextual determinants | *Journal of Ethnic and Migration Studies* | Demography Ethnic Studies | Netherlands | Longitudinal study | Ethnic group integration | Resistance to the social integration of foreigners was particularly strong among people with lower education, manual workers, the petty bourgeoisie and the unemployed. Furthermore, older respondents as well as those who were confronted with high unemployment during their adolescent years showed stronger resistance. With regard to period characteristics, we found that stronger resistance to the social integration of foreigners was not related to higher levels of unemployment and foreign immigration, but instead to recent increases in unemployment and foreign immigration. This suggests that it is not the actual level of ethnic competition, but the increasing level of ethnic competition that boosts negative attitudes toward foreigners. |
| 30 | Coile, CC and Levine, PB, 2007 | Labor market shocks and retirement: Do government programs matter? | *Journal of Public Economics* | Business & Economics | USA | Cross sectional study | Social security | This paper argues that labor market conditions are an important and often overlooked determinant of retirement transitions. In our analysis, we examine how the unemployment rate affects retirement and whether the Social Security (SS) system and Unemployment Insurance (UI) system influence how older workers respond to labor market shocks. We use pooled cross-sectional data from the March Current Population Survey (CPS) in our analysis. We find that downturns in the labor market increase retirement transitions and that the magnitude of this effect is comparable to that associated with moderate changes in financial incentives to retire and to the threat of a health shock facing older workers. Interestingly, retirements only increase in response to an economic downturn once workers become SS-eligible, suggesting that retirement benefits may help to alleviate the income loss associated with a weak labor market. This suggests that in some ways SS may serve as a more effective form of unemployment insurance for older workers than UI |
| 31 | Norman, P and Bambra, C, 2007 | Incapacity or unemployment? The utility of an administrative data source as an updatable indicator of population health | *Population Space and Place* | Demography Geography | England | Empirical | Sickness absence, hidden unemployment | A lack of annually accessible morbidity information for small geographical areas in England and Wales means that health studies are often restricted to using decennial, self-reported census, measures. Administrative data on health-related benefit claims, in the form of Incapacity Benefit (IB) data, are more regularly available and claimants are professionally diagnosed. This source may have the potential to be an annual indicator of population health. We examine IB as an indicator of population health at local government district and subdistrict levels by investigating distributions and relationships between IB and other health measures from the census and from mortality statistics. We found that relationships in 2001 between IB, census measures and mortality suggest that using IB as an indicator of population health will give very similar results, especially with those reporting themselves in the 2001 Census as 'permanently sick or disabled' and in the more urban areas. Although IB should be an objective measure as it is professionally diagnosed, we recognise that IB may hide unemployment and have inferred an estimate using census data on economic activity by exploring the relationship between IB and those reporting themselves permanently sick or disabled. This estimate suggests that previous assertions about the relationship between IB and unemployment may have been overzealous. On balance, IB is currently a useful indicator of relative health for small areas |
| 32 | Kyyrä, T and Wilke, RA, 2007 | Reduction in the long-term unemployment of the elderly:: A success story from Finland | *Journal of the European Economic Association* | Business & Economics | Finland | Empirical | Social security | In several European countries the elderly unemployed are allowed to collect unemployment benefits up to a certain age limit, after which they can retire via some early retirement scheme. In Finland the eligibility age of persons benefiting from this kind of scheme was raised from 53 to 55 in 1997. We consider layoff risks, unemployment durations, and the exit states before and after the reform. Since the reform the group aged 53-54 has had a lower risk of unemployment, shorter unemployment durations, and higher exit rates to employment, and it is almost indistinguishable from the group aged 50-52. We estimate that the amount of unemployment benefits saved due to the reform is close to EURO100 million for each age cohort turning 53. |
| 33 | Osterkamp, R and Röhn, O | Being on sick leave:: Possible explanations for differences of sick-leave days across countries | *CESIFO Economic Studies* | Business & Economics | Germany | Longitudinal study | Sick leave | On the basis of a panel for 20 countries and for the years 1996-2002, it is econometrically shown that the main explanatory factors are the generosity of granting sick leave, the strictness of employment protection and the employment of older persons. The unemployment rate and the employment of women-contrary to the result of some single-country studies-do not contribute to the explanation of sick-leave differences between countries. |
| 34 | Bambra, C and Norman, P, 2006 | What is the association between sickness absence, mortality and morbidity? | *Health & Place* | Public, Environmental & Occupational Health | England | Empirical | Incapacity benefit, sickness | This paper examines the area-level relationships in England and Wales between sickness absence ('incapacity benefit'), mortality and morbidity. It uses a random sample of incapacity benefit claims, and population counts of mortality and Census morbidity for local government districts. Although there is little correspondence between sickness absence claims by specific cause and mortality, all cause sickness absence has a strong relationship with all cause mortality (male r 0.74, p = 0.00; female r 0.64, p = 0.00) and it also has a very strong relationship with the Census measures of morbidity: LLTI (male r 0.98, p = 0.00; female r 0.97, p = 0.00) and 'not good health' (male r 0.99, p = 0.00; female r 0.96, p = 0.00). Incapacity benefit claims by all causes has the potential to provide an ongoing measure of area-level health in England and Wales. |
| 35 | van Orschot, W, 2006 | Making the difference in social Europe: deservingness perceptions among citizens of European welfare states | *Journal of European Social Policy* | Public Administration Social Issues | Netherlands | Cross sectional study | Value survey | Welfare stares treat different groups of needy people differently. Such differential rationing may reflect various considerations of policymakers, who act in economic, political and cultural contexts. This article alms at contributing to a theoretical and empirical understanding of the popular cultural context of welfare rationing. It examines European public perceptions of the relative deservingriess of four needy groups (elderly people, sick and disabled people, unemployed people, and immigrants). Hypotheses, deduced from a literature review, are tested against data from the 1999/2000 European Values Study survey. It is found that Europeans share a common and fundamental deservingness culture: across countries and social categories there is a consistent pattern that elderly people are seen as most deserving, closely followed by sick and disabled people; unemployed people are seen as less deserving still, and immigrants as least deserving of all. Conditionality is greater in poorer countries, in countries with lower unemployment, and in countries where people have less trust in fellow citizens and in state institutions. At the national level there is no relation with welfare regime type or welfare spending. Individual differences in conditionality are determined by several socio-demographic and attitudinal characteristics, as well as by certain features of the country people live in. |
| 36 | Gangl, M, 2006 | Scar effects of unemployment: An assessment of institutional complementarities | *American Sociological Review* | Sociology | USA | Longitudinal Study | Unemployment benefit system | This article uses panel data from the Survey of Income and Program Participation (SIPP) and the European Community Household Panel (ECHP) for a comparative analysis of workers'post-unemployment earnings trajectories in the United States and 12 Western European countries. Across the study sample of industrialized countries, results of difference-in-difference propensity score matching show post-unemployment earnings losses to be largely permanent and particularly significant for high-wage and older workers as well as for women. The analyses also show that negative effects of unemployment on workers 'subsequent earnings are mitigated through either generous unemployment benefit systems or strict labor market regulation. These effects stem partly from favorable behavioral responses that prevent downward occupational and industrial mobility and partly from changes in the overall structure of labor markets favoring the transferability of worker skills between jobs. These positive effects materialize despite the fact that labor market policies tend to successfully protect the core workforce from experiencing a job loss in the first place. |
| 37 | Hakola, T and Uusitalo, R, 2005 | Not so voluntary retirement decisions? Evidence from a pension reform | *Journal of Public Economics* | Business & Economics | Finland | Natural experiment | Pension reform | Firms may encourage their workers to retire early. Experience-rating of early retirement benefits creates incentives for firms to avoid this. We use a pension reform as a natural experiment in order to evaluate the effect of this experience-rating. The key result is that experience-rating of early retirement benefits reduces early exits of older workers. |
| 38 | Beatty, C and Fothergill, S , 2005 | The diversion from 'unemployment' to 'sickness' across British regions and districts | *Regional Studies* | Business & Economics Environmental Sciences & Ecology Geography Public Administration | England |  | Sickness | he diversion from 'unemployment' to 'sickness' across British regions and districts, Regional Studies 39, 837-854. Around 2.7 million non-employed adults of working age in the UK claim sickness-related benefits, and the numbers have risen steeply over time. The very large variation in the numbers across districts and regions points strongly to extensive hidden unemployment, especially in older industrial areas affected by job losses. This paper builds on two previous papers by the same authors - one dealing with the theoretical framework and the other with a local case study - to present wholly new estimates of the scale of the diversion across all parts of the country. It also questions contemporary perceptions of the UK labour market and the validity of current approaches to re-engaging sickness claimants with employment. |
| 39 | van Leuvensteijn, M and Koning, P, 2004 | The effect of home-ownership on labor mobility in the Netherlands | *Journal of Urban Economics* | Business & Economics Urban Studies | Netherlands | Longitudinal study | Labour mobility | Using a panel of individual labor and housing market histories for the period 1989-1998, we estimate a nonparametric model of both job durations and home-ownership. We do not find homeowners to change less from jobs than tenants. Instead, our results suggest that the housing decision is driven by job commitment, and not the reverse. We do however find homeowners to be less vulnerable to unemployment. |
| 40 | Beatty, C and Fothergill, S, 2002 | Hidden unemployment among men: A case study | *Regional Studies* | Business & Economics Environmental Sciences & Ecology Geography Public Administration | England | Empirical | Sickness, job loss | Using aggregate statistics and new survey evidence, it argues that these trends mask extensive hidden unemployment, especially among men on sickness-related benefits. Comparisons are also made with areas covered by similar surveys. The paper concludes that the claimant and International Labour Organization measures of unemployment both appear deeply flawed in the UK context because of the diversion from unemployment to sickness benefits. |
| 41 | Saunders, P, 2002 | Mutual obligation, participation and popularity: Social security reform in Australia | *Journal of Social Policy* | Public Administration Social Issues Social Work | Australia | national survey of public opinion | Welfare Reform, mutual obligation | The analysis indicates that there is support for mutual obligation for the young and long-term unemployed, but not for others, such as the older unemployed, those caring for young children and those with a disability. Most people also see mutual obligation as implying action on the part of government to reduce unemployment and ease the plight of the unemployed. |
| 42 | Clark, AE; Georgellis, Y and Sanfey, P, 2001 | Scarring: The psychological impact of past unemployment | *Economica* | Business & Economics | France | Longitudinal | Persistent unemployment | This paper considers the psychological impact of past unemployment. Using 11 waves of German panel data, we show that life satisfaction is lower not only for the current unemployed (relative to the employed), but also for those with higher levels of past unemployment. However, the negative wellbeing effect of current unemployment is weaker for those who have been unemployed more often in the past. The panel data also reveal some evidence that those suffering greater falls in wellbeing on entering unemployment are less likely to remain unemployed one year later. Together, these findings offer a psychological explanation of persistent unemployment. |
| 43 | DiPrete, TA; Goux, D; (...); Tåhlin, M, 2001 | Institutional determinants of employment chances.: The structure of unemployment in France and Sweden | *European Sociological Review* | Sociology | USA |  | Labour market structure & welfare state | Contrary to standard predictions derived from welfare-state theory, the unemployment structure of France does not conform to the classic insider-outsider labour-market model that scholars generally attribute to conservative welfare-state regimes. Instead, France has a flexible two-tier labour marker that produces relatively high entry rates into employment along with the strong age and educational gradients in exit rates that would be expected for a country with high firing costs. Even during the deep recession of the early 1990s, Sweden was also characterized by a strong age gradient in the rate of exit from an employer. However, Swedish rates do not show a strong education gradient, which is the expected consequence of Sweden's loosely linked school and work institutions, and extensive active labour-market policies. Active labour-market policies during the Swedish recession of the early 1990s appear to have further changed the shape of the age-unemployment curve in that country by raising the exit rate of older workers more than would have resulted from the dynamics of labour demand alone. |
| 44 | Blanchard, O and Portugal, P, 2001 | What hides behind an unemployment rate: Comparing Portuguese and US labor markets | *American Economic Review* | Business & Economics | USA | Empirical | Job creation | Unemployment duration is three times longer in Portugal than in the United States. Symmetrically, flows of workers into unemployment are three times lower in Portugal. These lower flows come in roughly equal proportions from lower job creation and destruction, and from lower worker flows given job creation and destruction. A plausible explanation is high employment protection in Portugal. High employment protection makes economies more sclerotic: but because it affects unemployment duration and worker flows in opposite directions, the effect on unemployment is ambiguous. |
| 45 | Marx, I, 2001 | Job subsidies and cuts in employers' social security contributions: The verdict of empirical evaluation studies | *International Labour Review* | Business & Economics | Belgium | Review | Employment subsidy | This article assembles findings from empirical evaluation studies of the effects of employment subsidies or reductions in employers' social security contributions aimed at stimulating recruitment of long-term unemployed persons and other vulnerable groups. Most suggest the net employment effects are modest to slight. The measured effects are consistently much lower than what most theoretical models and simulations predict, even under relatively pessimistic assumptions, mainly because of deadweight losses and, to a lesser extent, substitution effects. The available studies indicate that the impact of subsidies on beneficiaries' careers is limited, and possibly negative, except if coupled with training and job counselling. |
| 46 | Ruhm, CJ, 2000 | Are recessions good for your health? | *Quarterly Journal of Economics* | Business & Economics | USA |  | Suicide | This study investigates the relationship between economic conditions and health. Total mortality and eight of the ten sources of fatalities examined are shown to exhibit a procyclical fluctuation, with suicides representing an important exception. The variations are largest for those causes and age groups where behavioral responses are most plausible, and there is some evidence that the unfavorable health effects of temporary upturns are partially or fully offset if the economic growth is long-lasting. An accompanying analysis of microdata indicates that smoking and obesity increase when the economy strengthens, whereas physical activity is reduced and diet becomes less healthy. |
| 47 | Beatty, C; Fothergill, S and Macmillan, R, 2000 | A theory of employment, unemployment and sickness | *Regional Studies* | Business & Economics Environmental Sciences & Ecology Geography Public Administration | England | Empirical | Sickness, job loss | This paper explains how the measurement of unemployment is distorted by the way that 'sickness' is defined and counted by social security systems. Drawing on the concepts of 'hidden sickness', 'the queue for jobs' and 'hidden unemployment', and on empirical observations from the UK, it shows how job loss can result in increased recorded sickness rather than recorded unemployment, it also shows how this process may vary between localities and countries. The argument has profound implications for perceptions of the true extent of unemployment and of labour market disparities between regions. |
| 48 | Feld, LP and Kirchgässner, G, 2000 | Official and hidden unemployment and the popularity of the government:: an econometric analysis for the Kohl government | *Electoral Studies* | Government & Law | Switzerland | Empirical | Hidden unemployment | In this paper the relation between economic development and the popularity of the government and opposition in Germany is investigated for the period of the conservative-liberal coalition of chancellor Kohl, We estimate systems of popularity functions for the Old States from 1984 to 1996 and the New States from 1992 to 1996. It is shown that inflation and official as well as hidden unemployment have a negative impact on the government's popularity. Moreover, the de facto coalition between the SPD and the PDS in Sachsen-Anhalt in July 1994 strongly helped the Kohl government to become re-elected in October 1994. |
| 49 | MacKay, RR, 1999 | Work and nonwork: a more difficult labour market | *Environment and Planning A* | Environmental Sciences & Ecology Geography | Wales | Empirical | Sickness | Substantial labour reserves indicate that the labour market fails to discover a balance that reflects the needs and preferences of the population of working age. Different data sets-unemployment, vacancies, full-time equivalent jobs, and census data on forms of nonwork-are used to build a picture of the shift from tight to slack labour markets. The different sources confirm that unemployment becomes increasingly unreliable as a measure of labour reserve. The more difficult the labour market, the more likely it is that lack of opportunity takes the form of 'sickness' or government training rather than unemployment. |
| 50 | Fieldhouse, E and Hollywood, E, 1999 | Life after mining: Hidden unemployment and changing patterns of economic activity amongst miners in England and Wales, 1981-1991 | *Work, Employment, and Society* | Business & Economics Sociology | England | Empirical | Regional differences | Official counts of unemployment in the coalfields have not reflected the large-scale losses of thousands of jobs from the mining industry in the 1980s and 1990s. Recent studies have suggested that there are indeed high incidences of unemployment among ex-miners and that much of the unemployment in the coalfields is 'hidden', masked by the removal of miners from the official unemployment register through early retirement or being classed permanently sick. This paper examines how miners have been absorbed into the labour market over a ten-year period, between 1981 and 1991. Using data from the ONS Longitudinal Study a sample of miners are identified in 1981 and their labour market position in 1991 examined. The data are used to highlight changes in occupation, employment status and social class. In addition, regional differences in unemployment and joblessness are assessed. |
| 51 | Nickell, S, 1997 | Unemployment and labor market rigidities: Europe versus North America | *Journal of Economic Perspectives* | Business & Economics | England | Empirical | Rigidities in labour market | The received wisdom tells us that the rigidity and inflexibility of European job markets relative to that in the United States is the reason why Europe has high unemployment. This paper argues that this broad brush analysis is simply too vague to be useful. Indeed it is probably positively misleading. Many labour market institutions that conventionally come under the heading of rigidities have no observable impact on unemployment and may otherwise serve a useful purpose. |
| 52 | Henkens, K; Sprengers, M and Tazelaar, F, 1996 | Unemployment and the older worker in The Netherlands: Re-entry into the labour force or resignation | *Ageing and Society* | Geriatrics & Gerontology | Netherlands | Longitudinal study | Re-entry, discouraged workers | Re-entry via a 'normally' functioning labour market (formal application procedures and official job intermediaries) takes place almost exclusively during the first year after dismissal. If a new job is not found, a resigned feeling occurs among almost all of this older unemployed group. Most of the older people adjust to the new circumstances sooner or later. If one wishes to protect older persons from the trap of long-term unemployment (and in many cases total exclusion), more rapid interventions must be made, and reorientation and retraining efforts must be started sooner, before it is 'too late' From the employer's point of view, there is not much interest in those who are still unemployed after one year. |
| 53 | HUNT, J, 1995 | THE EFFECT OF UNEMPLOYMENT COMPENSATION ON UNEMPLOYMENT DURATION IN GERMANY | *Journal of Labour Economics* | Business & Economics | USA | Longitudinal study | Insurance, benefit | In West Germany during the 1980s, law changes cut the level of unemployment compensation for the unemployed without children and extended the duration of unemployment insurance for unemployed aged over 41. Analysis of these changes using the German Socioeconomic Panel shows that transitions from unemployment for those under 49 were particularly responsive to extensions of unemployment insurance. The implied elasticity for escapes of men to employment is similar to estimates for men in the United States, suggesting that differences in the potential duration of unemployment insurance only partially explain the difference in spell lengths between Germany and the United States. |
| 54 | MEYER, BD, 1990 | UNEMPLOYMENT-INSURANCE AND UNEMPLOYMENT SPELLS | *Econometrica* | Business & Economics Mathematics Mathematical Methods In Social Sciences | USA | Empirical | Unemployment benefit | This paper tests the effects of the level and length of unemployment insurance (UI)  benefits on unemployment durations. The paper particularly studies individual behavior  during the weeks just prior to when benefits lapse. Higher UI benefits are found to have a  strong negative effect on the probability of leaving unemployment. However, the probabil-  ity of leaving unemployment rises dramatically just prior to when benefits lapse. When the  length of benefits is extended, the probability of a spell ending is high in the week benefits  were previously expected to lapse. Individual data are used with accurate information on  spell durations, and the level and length of benefits. Semiparametric estimation tech-  niques are used and compared to alternative approaches. The semiparametric approach  yields more plausible estimates and provides useful diagnostics |
| 55 | Ruhm, CJ, 1990 | BRIDGE JOBS AND PARTIAL RETIREMENT | *Journal of Labr Economics* | Business & Economics | USA | Empirical | BRIDGE JOBS AND PARTIAL RETIREMENT | The "job-stopping" process of older workers often includes some combination of postcareer "bridge" employment, partial retirement, and reverse retirement. Fewer than two-fifths of household heads retire directly from career jobs, over half partially retire at some point in their working lives, and a quarter reenter the labor force after initially retiring. In addition, postcareer employment is frequently located outside the industry and occupation of the career job, and there are important differences in postcareer labor force experiences by gender, permanent income, and career-job pension status. |
| 56 | FRESE, M and MOHR, G, 1987 | PROLONGED UNEMPLOYMENT AND DEPRESSION IN OLDER WORKERS - A LONGITUDINAL-STUDY OF INTERVENING VARIABLES | *Social Science and Medicine* | Public, Environmental & Occupational Health Biomedical Social Sciences | Germany | Longitudinal study | Depression | In this study, unemployed blue-collar workers over the age of 45 (N = 51) filled out a questionnaire in 1975 and in 1977. It was shown that prolonged unemployment or re-unemployment leads to depression, reduced hope, and financial problems, although none of these factors leads to prolonged unemployment. Being employed or retired leads to a reduction of depression and financial problems. Problems associated with the daily hassles of unemployment, such as financial problems and disappointed hope play a role in the development of depression with prolonged unemployment. |
| 57 | PIACHAUD, D, 1986 | DISABILITY, RETIREMENT AND UNEMPLOYMENT OF OLDER MEN | *Journal of Social Policy* | Public Administration Social Issues Social Work | England | Census | Disability, retirement | Among older men in England from 1971 to 1981 the increase in the extent of those defined as disabled was of the same order as the increase in unemployment. Using Census data for the forty-six counties of England, changes in disability, retirement and economic activity are related to changes in unemployment. A substantial proportion of the overall increase in disability and the decline in economic activity is attributable to the general rise in unemployment. |
| 58 | TAYLOR, J | HIDDEN UNEMPLOYMENT, HOARDED LABOR, AND PHILLIPS CURVE | *Southern Economic Journal* | Business & Economics | England | Theoretical | Hidden unemployment | The rate of recorded unemployment  is not an efficient indicator of excess supply  in the labor market. To be an efficient indi-  cator of excess labor supply the unemploy-  ment variable would have to measure the  rate of underutilization of the labor supply.  This can be done once the unemployment  rate has been adjusted to include both hid-  den unemployment and hoarded labor. When  these two sources of excess labor supply are  included in the measurement of the rate of  unemployment, the unemployment series  should more accurately reflect movements in  excess labor suppl |

Older jobseekers (N=36)

| Research group | Author, year | Title | *Source* | [Field](http://ulrichsweb.serialssolutions.com/) | Geographical region (First author affiliation) | Type of study | Focus of paper | Main topic |
| --- | --- | --- | --- | --- | --- | --- | --- | --- |
| 1 | Svicher, A and Di Fabio, A | Job Crafting: A Challenge to Promote Decent Work for Vulnerable Workers | *Frontiers in Psychology* | Psychology | Italy | Non-empirical | Support for marginalised workers | the article deals with decent work and job crafting, starting with the definition of decent work according to the psychology of working theory (PWT) and examining the evolution of the construct of job crafting. |
| 2 | Friis, K; Ekholm, O; (...); Gronbiek, M, 2007 | Influence of health, lifestyle, working conditions, and socio-demography on early retirement among nurses: The Danish Nurse Cohort Study | *Scandinavian Journal of Public Health* | Public, Environmental & Occupational Health | Denmark | Longitudinal study | Working environment, nursing shortage | Having a spouse increased the probability of joining PEW, especially having a spouse who had retired or was unemployed. Conclusions: The retirement age among nurses is influenced by a number of sociodemographic, work-related, and health-related factors. Poor health, low income, living outside the Copenhagen area, being married, having a spouse who is outside the labor force, and working in the daytime are all predictors of early retirement among nurses. Poor working environment only marginally increased the probability of retiring early. |
| 3 | Farber, HS; Herbst, CM; (...); von Wachter, T, 2019 | Whom Do Employers Want? The Role of Recent Employment and Unemployment Status and Age | *Journal of Labour Economics* | Business & Economics | USA | Empirical | Job market discrimination | We use a resume audit study to investigate the role of employment and unemployment histories in callbacks to job applications. We also find that both younger and older applicants have a lower callback probability than prime-aged applicants. |
| 4 | Vlahov, 2007 | Factors Determining Callbacks to Job Applications by the Unemployed: An Audit Study | *RSF-The Russell Sage Journal of the Social Science* | Medical sciences | USA | Empirical | Job market discrimination | We use an audit study approach to investigate how unemployment duration, age, and holding a low-level interim job while applying for a better job affect the likelihood that experienced college-educated females applying for an administrative support job receive a callback from potential employers. First, the results show no relationship between callback rates and unemployment duration. Second, workers age fifty and older are significantly less likely to receive a callback. Third, taking an interim job significantly reduces the likelihood of receiving a callback. Finally, employers who have higher callback rates respond less to observable differences across workers in determining whom to call back. |
| 5 | Abrams, D; Swift, HJ and Drury, L, 2016 | Old and Unemployable? How Age-Based Stereotypes Affect Willingness to Hire Job Candidates | *Journal of Social Issues* | Medical sciences | UK | Empirical | Job market discrimination | This article draws on social role theory, age stereotypes and research on hiring biases, and reports three studies using age-diverse North American participants. These studies reveal that: (1) positive older age stereotype characteristics are viewed less favorably as criteria for job hire, (2) even when the job role is low-status, a younger stereotype profile tends to be preferred, and (3) an older stereotype profile is only considered hirable when the role is explicitly cast as subordinate to that of a candidate with a younger age profile. Implications for age-positive selection procedures and ways to reduce the impact of implicit age biases are discussed. |
| 6 | Oesch, D and Baumann, I, 2015 | Smooth transition or permanent exit? Evidence on job prospects of displaced industrial workers | *Socio-Economic Review* | Business & Economics Government & Law Sociology | Switzerland | Empirical | Displaced industrial workers | This article examines the job prospects of displaced industrial workers in Switzerland. Based on a survey of 1,203 workers who were dismissed after their manufacturing plants closed down, we analyse the determinants of re-employment, the sector of re-employment and the change in wages. Two years after displacement, a majority of workers were back in employment: 69% were re-employed, 17% unemployed and 11% retired. Amongst re-employed workers, two thirds found a job in manufacturing and one third in services. Contrary to a common belief, low-end services are not the collecting vessel of redundant industrial workers. Displaced workers aged 55 and older seem particularly vulnerable after a plant closes down: over 30% were long-term unemployed, and those older workers who found a new job suffered disproportionate wage losses. Advanced age-and not low education-appears as the primary handicap after mass redundancy. |
| 7 | Giesen, JM and Cavenaugh, BS, 2013 | Disability Insurance Beneficiaries with Visual Impairments in Vocational Rehabilitation: Socio-demographic Influences on Employment | *Journal of Visual Impairment & Blindness* | Rehabilitation | USA | Empirical | Disability benefit & Vocational rehabilitation services | This research provided new knowledge of state or agency influences, risk factors, advantages, and compensatory effects of services in blind agencies, and underscored the importance of prior work experience in achieving competitive employment for SSDI-beneficiary consumers. Implications for practitioners: Recommendations related to vocational rehabilitation services to SSDI beneficiaries, policy regarding agency structure, and future research are made. |
| 8 | Zacher, H, 2013 | Older job seekers' job search intensity: the interplay of proactive personality, age and occupational future time perspective | *Ageing & Society* | Geriatrics & Gerontology | Australia | Empirical | Job search | Long-term unemployment of older people can have severe consequences for individuals, communities and ultimately economies, and is therefore a serious concern in countries with an ageing population. However, the interplay of chronological age and other individual difference characteristics in predicting older job seekers' job search is so far not well understood. This study investigated relationships among age, proactive personality, occupational future time perspective (FTP) and job search intensity of 182 job seekers between 43 and 77 years in Australia. Results were mostly consistent with expectations based on a combination of socio-emotional selectivity theory and the notion of compensatory psychological resources. Proactive personality was positively related to job search intensity and age was negatively related to job search intensity. Age moderated the relationship between proactive personality and job search intensity, such that the relationship was stronger at higher compared to lower ages. One dimension of occupational FTP (perceived remaining time left in the occupational context) mediated this moderating effect, but not the overall relationship between age and job search intensity. Implications for future research, including the interplay of occupational FTP and proactive personality, and some tentative practical implications are discussed. |
| 9 | Beard, TR; Ford, GS; (...); Seals, RA, 2012 | Internet use and job search | *Telecommunications Policy* | Communication Information Science & Library Science Telecommunications | USA | Empirical | Job search | Regression analysis augmented with propensity score methods is used to estimate the effect of Internet use on job search. The formal distinction between the unemployed and the discouraged is exploited, where both desire employment but the latter has ceased active job search. Results indicate broadband use at home or at public locations reduces the probability that the unemployed cease job search by over 50% relative to unemployed persons who do not use the Internet at all. The results suggest that even public connections (e.g., at libraries) in unserved and underserved areas may produce substantial social benefits. (c) 2011 Elsevier Ltd. All rights reserved. |
| 10 | Orkin, FK; McGinnis, SL; (...); Garfield, JM, 2012 | United States Anesthesiologists over 50 Retirement Decision Making and Workforce Implications | *Anaesthesiology* | Anaesthesiology | USA | Cross sectional study | Burnout | Anesthesiologists (N = 3,222; response rate = 37%) reported a mean work week of 49.4 h and a mean retirement age of 62.7 yr, both values similar to those of other older physicians. Work week decreased with age, and part-time work increased. Women worked a shorter work week (mean, 47.9 vs. 49.7 h, P = 0.024), partly due to greater part-time work (20.2 vs. 13.1%, P value less than 0.001). Relative importance of factors reported among those leaving patient care differed by age cohort, subspecialty, and work status. Poor health was cited by 64% of anesthesiologists retiring in their 50s as compared with 43% of those retiring later (P = 0.039). This survey lends support for greater attention to potentially modifiable factors, such as workplace wellness and professional satisfaction, to prevent premature retirement. |
| 11 | Wanberg, CR, 2012 | The Individual Experience of Unemployment | *Annual Review of Psychology* | Psychology | USA | Review | reemployment | This review describes advances over the past decade in what is known about the individual experience of unemployment, predictors of reemployment; and interventions to speed employment. Research on the impact of unemployment has increased in sophistication, strengthening the causal conclusion that unemployment leads to declines in psychological and physical health and an increased incidence of suicide. This work has elucidated the risk factors and mechanisms associated with experiencing poor psychological health during unemployment; less so for physical health and suicide. Psychologists have begun to contribute to the study of factors associated with reemployment speed and quality. The past decade has especially illuminated the role of social networks and job search intensity in facilitating reemployment. Evidence suggests some individuals, especially members of minority groups, may face discrimination during their job search. Although more work in this arena is needed, several intervention-based programs have been shown to help individual get back to work sooner. |
| 12 | Bonoli, G and Hinrichs, K. 2012 | STATISTICAL DISCRIMINATION AND EMPLOYERS' RECRUITMENT Practices for low-skilled workers | *European Societies* | Sociology | Switzerland | Qualitative study | recruitment | This paper deals with the recruitment strategies of employers in the low-skilled segment of the labour market. We focus on low-skilled workers because they are overrepresented among jobless people and constitute the bulk of the clientele included in various activation and labour market programmes. A better understanding of the constraints and opportunities of interventions in this labour market segment may help improve their quality and effectiveness. On the basis of qualitative interviews with 41 employers in six European countries, we find that the traditional signals known to be used as statistical discrimination devices (old age, immigrant status and unemployment) play a somewhat reduced role, since these profiles are overrepresented among applicants for low skill positions. However, we find that other signals, mostly considered to be indicators of motivation, have a bigger impact in the selection process. These tend to concern the channel through which the contact with a prospective candidate is made. Unsolicited applications and recommendations from already employed workers emit a positive signal, whereas the fact of being referred by the public employment office is associated with the likelihood of lower motivation. |
| 13 | Orkin, FK; McGinnis, SL; (...); Garfield, JM, 2012 | United States Anesthesiologists over 50 Retirement Decision Making and Workforce Implications | *Anaesthesiology* | Anaesthesiology | USA | Cross sectional study | Burnout | Anesthesiologists (N = 3,222; response rate = 37%) reported a mean work week of 49.4 h and a mean retirement age of 62.7 yr, both values similar to those of other older physicians. Work week decreased with age, and part-time work increased. Women worked a shorter work week (mean, 47.9 vs. 49.7 h, P = 0.024), partly due to greater part-time work (20.2 vs. 13.1%, P value less than 0.001). Relative importance of factors reported among those leaving patient care differed by age cohort, subspecialty, and work status. Poor health was cited by 64% of anesthesiologists retiring in their 50s as compared with 43% of those retiring later (P = 0.039). This survey lends support for greater attention to potentially modifiable factors, such as workplace wellness and professional satisfaction, to prevent premature retirement. |
| 14 | Brough, P; Johnson, G; (...); Timms, C, 2011 | Comparisons of cognitive ability and job attitudes of older and younger workers | *Equality Diversity and Inclusion* | Business & Economics | Australia | Cross sectional study | Ageist stereotype | The purpose of this paper is to test social perceptions that older workers' cognitive performance and job attitudes compare adversely to their younger colleagues. The paper demonstrates that stereotypical assumptions concerning inadequate performance and low job commitment commonly attributed to older workers are not in fact indicative of all ageing employees. |
| 15 | Brough, P; Johnson, G; (...); Timms, C, 2011 | Comparisons of cognitive ability and job attitudes of older and younger workers | *Equality Diversity and Inclusion* | Business & Economics | Australia | Cross sectional study | Ageist stereotype | The purpose of this paper is to test social perceptions that older workers' cognitive performance and job attitudes compare adversely to their younger colleagues. The paper demonstrates that stereotypical assumptions concerning inadequate performance and low job commitment commonly attributed to older workers are not in fact indicative of all ageing employees. |
| 16 | Wang, M; Zhan, YJ; (...); Shultz, KS, 2008 | Antecedents of bridge employment: A longitudinal investigation | *Journal of Applied Psychology* | Psychology Business & Economics | USA | Longitudinal study | Bridge employment, life course perspective | Bridge employment is the labor force participation pattern increasingly observed in older workers between their career jobs and their complete labor force withdrawal. It serves as a transition process from career employment to full retirement. Typical bridge employment decisions include full retirement, career bridge employment, and bridge employment in a different field. In the current study, 3 dominant theories (i.e., role theory, continuity theory, and life course perspective) on retirement processes were reviewed. On the basis of these theories, the authors proposed 4 categories of antecedents (i.e., individual attributes, job-related psychological variables, family-related variables, and a retirement-planning-related variable) of different types of bridge employment decisions. The authors used longitudinal data of a large, nationally representative sample from the Health and Retirement Study (F. Juster & R. Suzman, 1995) to test the current hypotheses. These data were analyzed with multinomial logistic regression, and most of the hypotheses were supported by the results. The implications of this study are discussed at both theoretical and practical levels. |
| 17 | McQuaid, RW, 2006 | Job search success and employability in local labor markets | *Annals of Regional Science* | Business & Economics Environmental Sciences & Ecology Geography Public Administration | Scotland |  | Spatial mismatch | The geographic accessibility to local jobs was significantly and positively associated with job search success. The results suggest that a range of employability factors and both skills mismatch and spatial mismatch are important in explaining job search success. The degree of "skills" or "spatial" mismatch in a local labor market will be contingent upon the characteristics of the local economy, employers, job seekers and the jobs being considered. |
| 18 | McQuaid, RW; Green, A and Danson, M, 2005 | Introducing employability | *Urban Studies* | Environmental Sciences & Ecology Urban Studies | Scotland | Conceptual | employability |  |
| 19 | Andrews, J; Manthorpe, J and Watson, R, 2005 | Employment transitions for older nurses: a qualitative study | *Journal of Advanced Nursing* | Nursing | England | Qualitative study | Flexible work, retention | Employers, policy makers and advisers and older nurses all identified a range of influences on nurses' employment decisions including a lack of flexible hours, the stress of work, pension-related expectations and the pace of change. Some of these related to negative aspects of work that led nurses to leave ('push' factors), and others to the presence of positive factors in nursing or in pension options ('pull' factors). |
| 20 | Creed, PA and Klisch, J, 2005 | Future outlook and financial strain: Testing the personal agency and latent deprivation models of unemployment and well-being | *Journal of Occupational Health Psychology* | Public, Environmental & Occupational Health Psychology | Australia | Empirical | Hopelessness scale, mental health | sample (N = 239) of unemployed adults completed scales measuring well-being, financial strain, future outlook, and the latent benefits of employment. The study tested (a) the relative contributions of the latent deprivation and agency restriction models in predicting well-being and (b) whether financial strain interacted with future outlook to predict well-being or whether financial strain was mediated by future outlook. The authors found support for agency restriction over the latent deprivation model and concluded that examining internal personal agency processes is required to explain the decline in well-being. No interaction effects were identified for financial strain and future outlook, but future outlook did mediate the influence of financial strain. Age and length of unemployment were also associated with well-being. |
| 21 | Adams, G and Rau, B, 2004 | Job seeking among retirees seeking bridge employment | *Personnel Psychology* | Psychology Business & Economics | USA |  | Job search behaviour | Generally, the results support the efficacy of the Wanberg et al. model to predict job seeking among this group. Biographical variables such as older worker job search constraints, self-evaluations (e.g., job seeking self-efficacy), and motive/social variables (e.g., social support) were related to job seeking. Some of these, however, were not in the expected direction. Similarities and differences between "regular" job seeking and bridge employment job seeking are discussed. |
| 22 | Creed, PA and Watson, T, 2003 | Age, gender, psychological wellbeing and the impact of losing the latent and manifest benefits of employment in unemployed people | *Australian Journal of Psychology* | Psychology | Australia | Empirical | Mental health | The young unemployed reported higher wellbeing, more social support, and higher status than the mature group, and less time structure and higher status than the middle-aged group. |
| 23 | Kulik, L, 2001 | Impact of length of unemployment and age on jobless men and women: a comparative analysis | *Journal of Employment Counseling* | Psychology | Israel |  | Gender | The findings revealed that job search intensity, psychological stress, and work centrality were highest among participants who had been unemployed for 2 to 3 months, and gradually declined for longer periods of unemployment. Moreover, middle-aged participants spent more hours per week searching for jobs and mentioned fewer advantages of unemployment than did the younger groups. Furthermore, women reported a sharper decline in health as a result of unemployment, as well as lower levels of work centrality. |
| 24 | Kanfer, R; Wanberg, CR and Kantrowitz, TM, 2001 | Job search and employment: A personality - Motivational analysis and meta-analytic review | *Journal of Applied Psychology* | Psychology Business & Economics | USA |  | Coping | A motivational, self-regulatory conceptualization of job search was used to organize and investigate the relationships between personality, expectancies, self, social, motive, and biographical variables and individual differences in job search behavior and employment outcomes. Meta-analytic results indicated that all antecedent variables, except optimism, were significantly related to job search behavior, with estimated population correlations ranging from -.15 to .46. As expected, job search behavior was significantly and positively related to finding employment. Several antecedents of job search were also significantly related to employment success, although the size of these relationships was consistently smaller than those obtained for job search. Moderator analyses showed significant differences in the size of variable relationships for type of job search measure (effort vs. intensity) and sample type (job loser vs. employed job seeker vs. new entrant). |
| 25 | Weckerle, JR and Shultz, KS, 1999 | Influences on the bridge employment decision among older USA workers | *Journal of Occupational and Organizational Psychology* | Psychology Business & Economics | USA |  | Bridge retirement | Results of a direct discriminant function analysis showed that three of the four variables-voluntariness of retirement, anticipated financial reward and flexibility-significantly distinguished older workers in the four retirement/employment decision categories. The implications of the results for both individuals and organizations are discussed. |
| 26 | Russell, H, 1999 | Friends in low places: Gender, unemployment and sociability | *Work, Employment and Society* | Business & Economics Sociology | Ireland | Survey | Social exclusion, social network | It is found that women's previous pattern of labour market participation is critical in building up a social network which is resistant to unemployment. However a social network that is external to the labour market may also have some negative implications. An absence of friends in employment could lead to a detachment from the world of work. The networks of unemployed men and women are found to feature a much higher than normal concentration of unemployed members and are deficient in employment contacts. Contrary to underclass predictions this does not lead to a reduction in employment commitment but it does have repercussions for the availability of support and access to job information. |
| 27 | Murphy, GC and Athanasou, JA,1999 | The effect of unemployment on mental health | *Journal of Occupational and Organizational Psychology* |  |  | Longitudinal study | Reemployment | Sixteen recent longitudinal studies are examined for evidence relevant to the claim that a change to one's employment status affects one's mental health. Although there were Limitations to the set of studies examined, most of the studies supported this claimed relationship. Examination was then made of the size of this effect. In carrying out this examination, the set of study results were divided into two categories: (a) those addressing the question of the extent to which gaining employment impacts on mental well-being; (b) those addressing the question of the extent to which employment loss impacts on mental health. The meta-analyses indicated that there was a weighted effect size of .54 for the first question, and a smaller weighted effect size (.36) for the latter question. |
| 28 | Lay, CH and Brokenshire, R, 1997 | Conscientiousness, procrastination, and person-task characteristics in job searching by unemployed adults | *Current Psychology* | Psychology | Canada |  | Job search, procrastination | The study examined the job-search intentions and subsequent behavior of 32 unemployed males and 32 unemployed females, median age of 43 years, in relation to conscientiousness and the lower-order trait procrastination and to rated person-task characteristics of importance, pleasantness, and competence regarding 14 job-search activities. |
| 29 | RIFE, JC and BELCHER, JR, 1994 | ASSISTING UNEMPLOYED OLDER WORKERS TO BECOME REEMPLOYED - AN EXPERIMENTAL EVALUATION | *Research on Social Work Practice* | Social Work | USA | Experimental evaluation | Job search support intervention | This article presents the findings from an evaluation and replication of a Job Club intervention strategy designed to assist older workers in regaining employment. Using an experimental pre-post control group design, the results indicate that Job Club programs are more effective than traditional job search assistance strategies in helping participants obtain employment. The benefits of using behavioral strategies such as the Job Club in social work practice with unemployed older workers are discussed. |
| 30 | RIFE, JC and BELCHER, JR, 1993 | SOCIAL SUPPORT AND JOB SEARCH INTENSITY AMONG OLDER UNEMPLOYED WORKERS - IMPLICATIONS FOR EMPLOYMENT COUNSELORS | *Journal of Employment Counseling* | Psychology | USA | Qualitative study | Job search support intervention | A total of 54 unemployed workers over the age of 50 were interviewed in order to examine the relationship between the quality of social support provided by family and friends for their job search efforts and their job search intensity. The findings indicate that receiving positive social support for job search activities was significantly related to job search intensity. In addition, older workers perceived the supportive messages provided by unemployed friends as more positive than the support messages provided by employed and retired family or friends. Implications of these findings for assessing the presence of positive social supports and for developing successful job search assistance programs for older unemployed workers are discussed. |
| 31 | FEATHER, NT, 1989 | REPORTED CHANGES IN BEHAVIOR AFTER JOB LOSS IN A SAMPLE OF OLDER UNEMPLOYED MEN | *Australian Journal of Psychology* | Psychology | Australia | Empirical | Job loss | Reported financial stress and financial strain were both positively related to psychological distress and negatively related to life satisfaction and quality of life. The results were taken to indicate the importance of the availability of money and goal-directed activities for unemployed people. |
| 32 | ROWLEY, KM and FEATHER, NT, 1987 | THE IMPACT OF UNEMPLOYMENT IN RELATION TO AGE AND LENGTH OF UNEMPLOYMENT | *Journal of Occupational Psychology* | Psychology | Australia | Empirical | Job seeking activity | The study investigated the impact of length of unemployment in two groups of  unemployed men in Adelaide, South Australia. The two groups were selected so as to  sample two different age ranges, 15 to 24 years and 30 to 49 years. Both groups  completed scales concerned with job-seeking activity, employment commitment, selfesteem, psychological distress, use of time, and financial strain. The results indicated  differences between the two age groups on some of these variables with more psychological distress, higher self-esteem, and greater employment commitment reported by  subjects in the older group. |
| 33 | WARR, P and JACKSON, P, 1985 | FACTORS INFLUENCING THE PSYCHOLOGICAL IMPACT OF PROLONGED UNEMPLOYMENT AND OF RE-EMPLOYMENT | *Psychological Medicine* | Psychology Psychiatry | England | Longitudinal study | Re-employment | Unemployed men were re-interviewed 9 months after initial measurement of their psychological health and commitment to the labour market. Multiple regression analyses were used to examine factors associated with magnitude of changes during continuous unemployment, yielding a systematic pattern of significant relationships. For example, higher employment commitment at initial interview was significantly associated with a greater subsequent decline in psychological health, but not in physical health; reporting a chronic health impairment at initial interview was significantly associated with a greater subsequent decrement in physical health, but not in psychological health. |
| 34 | WARR, P and JACKSON, P, 1984 | MEN WITHOUT JOBS - SOME CORRELATES OF AGE AND LENGTH OF UNEMPLOYMENT | *Journal of Occupational Psychology* | Psychology | England | Empirical | Financial strain | Measures of reported health change, financial stress, financial strain, and commitment to the labour market were used with a sample of 954 unemployed working class men, selected to cover equally all levels of age and several levels of length  of unemployment. It was found that psychological deterioration, financial stress  and strain, and labour market commitment were greatest among middle-aged  unemployed men. Deterioration in health and degree of financial strain were  found to be greater among men unemployed for longer periods in contrast with  those who had more recently become unemployed, but no association was  observed between length of unemployment and the measures of financial stress  and commitment to the labour market. The probability of having an employed  wife was found to be negatively associated with length of unemployment, consistent with the operation of a household strategy for income maintenance. |
| 35 | Hepworth, S, 1980 | Moderating factors of the psychological impact of unemployment | *Journal of Occupational Psychology* | Psychology | England | Cohort study | Mental health | This study is an exploration of some of the factors important in determining a man's subjective reaction to the experience of unemployment. Goldberg's General Health Questionnaire was used as a normative measure of mental health, and a Present Life Satisfaction Scale was used as a measure of subjective well-being. Length of unemployment was inversely correlated with mental health and well-being; and semi-skilled and unskilled men had poorer psychological well-being during unemployment than those of higher occupational status. The best single predictor of mental health during unemployment was whether or not a man felt his time was occupied. |
| 36 | Jahoda, M, 1979 | IMPACT OF UNEMPLOYMENT IN THE 1930S AND THE 1970S | *Bulletin of the British Psychological Society* | Psychology | England | Review | Impact of welfare state | She raised some crucial questions about the relationship between tl1e 1930s  findings ,and the unemployed today. Tl1ree n1a·or changes in social conditions between then  and now could. affect tl1e validity of any generalizations from 1  early studies to the current  situation: 1 With the advent of the Welfare State, the unemployed in Western countries  while still disadvantaged financially compared to tl1ose in work do not as a rule suffer tl1e  severe econon1ic hardship that the unemployed of the 1930s l1ad to face How mucl1 did  factors such ·as poor nutrition, for example, contribute to the well-documented psychological ill-effects? (2) Tl1e unemployed 110w are considerably better educated than they  were during the l 930s. This could result in greater disappointment because of increased  expectations, or it might enable tl1e unemployed 1  to make more constructive use of their  leisure time· 3) Changes in the social value attacl1ed to work may have an1eliorated the  deleterious effects of unemployment. J al1oda also reminded the readers tl1at we now have  an extensive literature on the psychology of work wlticl1 l1ould enable us to better  comprehend the meaning ,of lack  of work .. |

Life course of inequalities/ SDH (N=27)

| Research group | Author, year | Title | *Source* | [Field](http://ulrichsweb.serialssolutions.com/) | Geographical region (First author affiliation) | Type of study | Focus of paper | Main topic |
| --- | --- | --- | --- | --- | --- | --- | --- | --- |
| 1 | Soto-Simeone, A and Kautonen, T (2020) | Retirement Sequences of Older Americans: Moderately Destandardized and Highly Stratified Across Gender, Class, and Race | *Gerontologist* | Geriatrics & Gerontology | USA | Empirical | Life course inequalities on retirement patterns | A sequence-analytic approach that models group differences uncovers misjudgments about the degree of destandardization of retirement sequences. When a continuous process is represented as individual transitions, the overall pattern of retirement sequences gets lost and appears destandardized. These patterns get further complicated by differences in social structures by gender, class, and race in ways that seem to reproduce advantages that men, more highly educated individuals, and Whites enjoy in numerous areas over the life course. |
| 2 | Brüderl, J; Kratz, F and Bauer, G, 2019 | Life course research with panel data: An analysis of the reproduction of social inequality | *Advances in Life Course Research* | Social science | Germany | Empirical | reproduction of social inequality | Results show that higher social origin indeed relates to higher well-being, and that the well-being differential increases with age. Further, unemployment plays no significant role in mediating origin-specific effects of age on well-being. |
| 3 | Wahrendorf, M; Zaninotto, P; (...); Carr, E, 2018 | Late Life Employment Histories and Their Association With Work and Family Formation During Adulthood: A Sequence Analysis Based on ELSA | *Journals of Gerontology Series B- Psychological Sciences and Social Sciences* | Geriatrics & GerontologyPsychology | Germany | Sequence analysis | employment histories in later life and testing their links to prior life course conditions | Three clusters include histories dominated by full-time employees but with varying age of retirement (before, at, and after age 60). One cluster is dominated by self-employment with comparatively later retirement. Remaining clusters include part-time work (retirement around age 60 or no retirement), continuous domestic work (mostly women), or other forms of nonemployment. Those who had strong attachments to the labor market during adulthood are more likely to have histories of full-time work up until and beyond age 60, especially men. Parenthood in early adulthood is related to later retirement (for men only). Continued domestic work was not linked to parenthood. Partnered women tend to work part-time or do domestic work. The findings remain consistent after adjusting for birth cohort, childhood adversity, life course health, and occupational position. |
| 4 | Zhang, CS; Brook, JS; (...); Brook, DW, 2016 | Trajectories of marijuana use from adolescence to adulthood as predictors of unemployment status in the early forties | *American Journal on Addictions* | Substance Abuse | USA | Cohort study | Earlier life determinants for unemployment | the associations between the distinct trajectories of marijuana use and unemployment in early midlife indicate that it is important to develop intervention programs targeting chronic marijuana use as well as unemployment in individuals at this stage of development. Results from this study should encourage clinicians, teachers, and parents to assess and treat chronic marijuana use in adolescents. |
| 5 | Sallis, 2016 | Travel mobility and social participation among older people in a transit metropolis: A socio-spatial-temporal perspective | *Transportation Research Part A- Policy and Practice* | Business & Economics Transportation | China | Empirical | Barriers to unemployment | Separate models were estimated for different departure times over the course of a day. We found that the travel mobility of older people in Hong Kong was not significantly lower compared with younger people. The steep decline in mobility with ageing as shown in the Western experience was not found in our study. On the other hand, some older people associated with certain socio-economic and geographic characteristics were found to face potential spatial barriers in fulfilling their mobility needs during certain periods of the day. |
| 6 | Danielsson, AK; Falkstedt, D; (...); Agardh, E, 2015 | Cannabis use among Swedish men in adolescence and the risk of adverse life course outcomes: results from a 20 year-follow-up study | *Addiction* | Substance AbusePsychiatry | Sweden | Cohort study | Earlier life determinants for unemployment | Heavy cannabis use among Swedish men in late adolescence appears to be associated with unemployment and being in need of social welfare assistance in adulthood. These associations are not explained fully by other health-related, social or behavioural problems. |
| 7 | Brand, JE, 2015 | The Far-Reaching Impact of Job Loss and Unemployment | *Annual Review of Sociology* | Sociology | USA | Meta analysis | Unemployment spillover | Research suggests that displacement is associated with subsequent unemployment, long-term earnings losses, and lower job quality; declines in psychological and physical well-being; loss of psychosocial assets; social withdrawal; family disruption; and lower levels of children's attainment and well-being. Although reemployment mitigates some of the negative effects of job loss, it does not eliminate them. Contexts of widespread unemployment, although associated with larger economic losses, lessen the social-psychological impact of job loss. Future research should attend more fully to how the economic and social-psychological effects of displacement intersect and extend beyond displaced workers themselves. |
| 8 | Wiemers, EE, 2014 | The Effect of Unemployment on Household Composition and Doubling Up | *Demography* | Demography | USA | Longitudinal study design | SDH | find that individuals who become unemployed are three times more likely to move in with other people. Moving into shared living arrangements in response to unemployment is not evenly spread across the distribution of educational attainment: it is most prevalent among individuals with less than a high school diploma and those with at least some college. |
| 9 | Strandh, M; Winefield, A; (...); Hammarström, A, 2014 | Unemployment and mental health scarring during the life course | *European Journal of Public Health* | Public, Environmental & Occupational Health | Sweden | Empirical | Mental health | There has been little research on the long-term relationship between unemployment experiences and mental health over the life course. This article investigates the relationship between youth unemployment as well as that of unemployment experiences during later periods and mental health at ages 16, 21, 30 and 42 years. Youth unemployment was shown to be significantly connected with poorer mental health at all three target ages, 21, 30 and 42 years. Later singular unemployment experiences did not appear to have the same long-term negative effects. There was however an accumulation in poorer mental health among respondents with unemployment experiences during two, and even more so three, of the periods. Conclusion: There are long-term mental health scarring effects of exposure to youth unemployment and multiple exposure to unemployment during the life course. |
| 10 | Singh, GK; Siahpush, M and Altekruse, SF. 2013 | Time Trends in Liver Cancer Mortality, Incidence, and Risk Factors by Unemployment Level and Race/Ethnicity, United States, 1969-2011 | *Journal of Community Health* | Health Care Sciences & Services Public, Environmental & Occupational Health | USA | Empirical | unemployment and racial/ethnic disparities in liver cancer mortality | Although liver-cancer mortality rose markedly for all groups during 1969-2011, higher unemployment levels were associated with increased mortality and incidence rates in each time period. Both absolute and relative inequalities in liver cancer mortality according to unemployment level increased over time for both males and females and for those aged 25-64 years. Compared to the lowest-unemployment group, those aged 25-64 in the highest-unemployment group had 56 and 115 % higher liver-cancer mortality in 1969-1971 and 2005-2009, respectively. Regardless of unemployment levels, Asian/Pacific Islanders and Hispanics had the highest mortality and incidence rates. The adjusted odds of hepatitis infection and heavy drinking were 38-39 % higher among the unemployed than employed. Liver-cancer mortality and incidence have risen steadily among all racial/ethnic, sex, and socioeconomic groups. Faster increases in mortality among the highest-unemployment group have led to a widening gap in mortality over time. Disparities in mortality and incidence are consistent with similar inequalities in hepatitis infection and alcohol consumption. |
| 11 | Zavras, D; Tsiantou, V; (...); Kyriopoulos, J | Impact of economic crisis and other demographic and socio-economic factors on self-rated health in Greece | *European Journal of Public Health* | Public, Environmental & Occupational Health | Greece | Empirical | SDH | Poor self-rated health was most common in older people, unemployed, pensioners, housewives and those suffering from chronic disease. Men, individuals with higher education and those with higher income have higher probability to report better self-rated health. Furthermore, the probability of reporting poor self-rated health is higher at times of ecoSnomic crisis. Conclusion: Our findings confirm the association of self-rated health with economic crisis and certain demographic and socio-economic factors. Given that the economic recession in Greece deepens, immediate and effective actions targeting health inequalities and improvements in health status are deemed necessary. |
| 12 | Daly, M and Delaney, L, 2013 | The scarring effect of unemployment throughout adulthood on psychological distress at age 50: Estimates controlling for early adulthood distress and childhood psychological factors | *Social Science & Medicine* | Public, Environmental & Occupational Health Biomedical Social Sciences | Scotland | Cohort study | Earlier life determinants for unemployment | Unemployment is an established predictor of psychological distress. Despite this robust relationship, the long-term impact of unemployment on human welfare has been examined in relatively few studies. In this investigation we test the association between the life-time duration of unemployment over a 34 year period from 1974 to 2008 and psychological distress at age 50 years in a sample of 6253 British adults who took part in the National Child Development Study (NCDS). In addition to adjusting for demographic characteristics, we account for the role of childhood psychological factors, which have been shown to predict adult occupational and mental health outcomes and may determine the connection between unemployment and distress. We find that intelligence and behavioral/emotional problems at age 11 predict both unemployment and psychological distress later in life. Furthermore, as predicted, the duration of unemployment throughout adulthood was associated with elevated levels of psychological distress at age 50, after adjusting for demographic characteristics including labor force status at age 50. |
| 13 | Reine, I; Novo, M and Hammarström, A, 2013 | Unemployment and ill health - A gender analysis: Results from a 14-year follow-up of the Northern Swedish Cohort | *Public Health* | Public, Environmental & Occupational Health | Sweden | Cohort study | SDH | Long-term unemployment at a young age could have various health effects in men and women. At present, the mechanisms behind the health consequences are better understood among women. Research would benefit from developing theories in order to explain how youth unemployment leads to gendered health consequences. |
| 14 | Astell-Burt, T and Feng, XQ, 2013 | Health and the 2008 Economic Recession: Evidence from the United Kingdom | *Plos One* | Science & Technology - Other Topics | Australia | Longitudinal | Health inequalities | Although our study found no exacerbation of pre-recession health inequalities, the rise in poor health status not only for the unemployed, but also among people who remained employed, regardless of their occupational class, justifies concern voiced among many public health commentators. |
| 15 | Frech, A and Damaske, S, 2012 | The Relationships between Mothers' Work Pathways and Physical and Mental Health | *Journal of Health and Social Behaviour* | Public, Environmental & Occupational Health Psychology Biomedical Social Sciences Sociology | USA | Longitudinal | Health inequalities | We contribute to research on the relationships between gender, work, and health by using longitudinal, theoretically driven models of mothers' diverse work pathways and adjusting for unequal selection into these pathways. Using the National Longitudinal Study of Youth-1979 (N = 2,540), we find full-time, continuous employment following a first birth is associated with significantly better health at age 40 than part-time work, paid work interrupted by unemployment, and unpaid work in the home. Part-time workers with little unemployment report significantly better health at age 40 than mothers experiencing persistent unemployment. These relationships remain after accounting for the unequal selection of more advantaged mothers into full-time, continuous employment, suggesting full-time workers benefit from cumulating advantages across the life course and reiterating the need to disentangle health benefits associated with work from those associated with pre-pregnancy characteristics. |
| 16 | Sigurdsson, SO; Ring, BM; (...); Silverman, K, 2012 | Barriers to Employment among Unemployed Drug Users: Age Predicts Severity | *American Journal of Drug and Alcohol Abuse* | Psychology Substance Abuse | USA | Empirical | Barriers to employment | Drug users in treatment or exiting treatment face many barriers to employment when entering the job market, such as low levels of education and technical skills, and low levels of interpersonal skills. As a result of these and other barriers, employment rates in these groups are generally low. These results suggest that providers of workforce development services for drug users in treatment or exiting treatment should attend to these specific barriers to employment, which may also be more pronounced among older clients. |
| 17 | Hammarström, A; Gustafsson, PE; (...); Janlert, U, 2011 | It's no surprise! Men are not hit more than women by the health consequences of unemployment in the Northern Swedish Cohort | *Scandinavian Journal of Public Health* | Public, Environmental & Occupational Health | Sweden | Cohort study | Health inequalities | In multivariate logistic regression analyses significant relations between unemployment and mental health/smoking were found among both women and men, even after control for unemployment at the time of the investigation and indicators of health-related selection. Significant relations between unemployment and alcohol consumption were found among women, while few visits to a dentist was significant among men. Conclusions: Men are not hit more by the health consequences of unemployment in a Swedish context, with a high participation rate of women in the labour market. The public health relevance is that the study indicates the need to take gendered contexts into account in public health research. |
| 18 | Puig-Barrachina, V; Malmusi, D; (...); Benach, J, 2011 | MONITORING SOCIAL DETERMINANTS OF HEALTH INEQUALITIES: THE IMPACT OF UNEMPLOYMENT AMONG VULNERABLE GROUPS | *International Journal of Health Services* | Health Care Sciences & Services | Spain | Cross sectional study | SDH | the authors identified seven vulnerable groups to monitor. Primary findings indicate that unemployment has a greater adverse effect on the mental health of male manual workers, single mothers, main-earner women, and manual workers without unemployment benefits for both sexes. Findings support the need to devote more research to the surveillance of unemployment as a social determinant of health inequalities, to identify additional unemployment indicators, and to consider how various social mechanisms of inequality interact with each other to produce health inequalities among vulnerable groups. |
| 19 | Giatti, L; Barreto, SM and César, CC, 2008 | Household context and self-rated health: the effect of unemployment and informal work | *Journal of Epidemiology and Community Health* | Public, Environmental & Occupational Health | Brazil | Longitudinal study | SDH | This article investigates whether the presence of residents in precarious work situations influences the self-rated health of people living in the same household. These results suggest that unemployment and/or informal work have a contextual impact on the self-rated health of household dwellers. They add to the hypothesis that adverse conditions in the labour market and their impact on the health of individuals and groups are relevant in understanding inequalities in health. |
| 20 | Fagan, P; Shavers, V; (...); Ponder, P, 2007 | Cigarette smoking and quitting behaviors among unemployed adults in the United States | *Nicotine & Tobacco Research* | Substance Abuse Public, Environmental & Occupational Health | USA | Cross sectional study | SDH | Among the unemployed, 35% were current smokers and 13% were former smokers. Of the former smokers, 81% quit successfully for at least 12 months. Participants with family incomes of less than US$25,000 were more likely than those with incomes of $50,000 or more to currently smoke (OR=2.13, 95% CI=1.85-2.46). Service workers and blue-collar workers were less likely than white-collar workers to report former smoking. Participants unemployed for 6 months or more were twice as likely as those unemployed for less than 6 months to quit successfully (OR=2.05, 95% CI=1.07-3.95). Unemployed blue-collar workers had a greater odds ratio of successfully quitting than white-collar workers (OR=1.83, 95% CI=1.17-2.87). Smoking rates were high among the unemployed, and quitting behaviors varied by sociodemographic factors and length of unemployment. Studies are needed to examine the feasibility of cessation interventions for the unemployed. |
| 21 | Bullock, K; Crawford, SL and Tennstedt, SL, 2003 | Employment and caregiving: Exploration of African American caregivers | *Social Work* | Social Work | USA | Empirical | INFORMAL CARE | To more completely understand the challenges African American families face, when combining employment commitments and informal caregiving responsibilities, the authors used data from a community sample of 119 African American elder-caregiver dyads. This article examines the nature of caregiving relationships and extent to which caregivers' employment statuses affect the hours of care provided. The authors concluded that employed caregivers do not provide significantly less care than do unemployed caregivers, elderly people with employed caregivers are no more likely than those with unemployed caregivers to use formal services, and unemployed caregivers may remain unemployed partly because of caregiving responsibilities. |
| 22 | Yu, WH, 2002 | Jobs for mothers: Married women's labor force reentry and part-time, temporary employment in Japan | *Sociological Forum* | Sociology | Taiwan | Empirical | Gender, labour force re-entry | In Japan, both the labor supply of middle-aged women and the demand for part-time workers have increased, but these conditions channel middle-aged women into part-time or temporary employment only when systematic barriers obstruct their access to full-time jobs. Because it plays an important role in women's employment decisions, the rigidity of standard, full-time employment needs greater attention in studies of nonstandard, atypical types of work. |
| 23 | Fokkema, T, 2002 | Combining a job and children: contrasting the health of married and divorced women in the Netherlands? | *Social Science & Medicine* | Public, Environmental & Occupational Health Biomedical Social Sciences | Netherlands | survey | Divorce, employment | The findings show that combining a job outside the home and childcare does not harm women's health, irrespective of the length of the working week and the age of the children. In fact, some work-childcare combinations are associated with better health. This is true foe both married and divorced women and especially holds true in the case of a part-time job and having older children. Two effects are responsible for the findings: enjoying good health enables mothers to work outside the home (selection effect) and working outside the home promotes mothers' health (health effect). |
| 24 | Green, RK and Hendershott, PH, 2001 | Home-ownership and unemployment in the US | *Urban Studies* | Environmental Sciences & Ecology Urban Studies | USA | Cross sectional study | Mortgage | Young households have accumulated little wealth and have had less time to become attached to the geographical area than middle-aged households and thus are more likely to respond to unemployment by relocating. Older households' employment cannot be greatly affected by home-ownership because their members are largely not in the labour force. Unemployment rates of household heads are affected less by tenure than those of the population as a whole. |
| 25 | von Bonsdorff, ME; Shultz, KS; (...); Tansky, J, 2009 | THE CHOICE BETWEEN RETIREMENT AND BRIDGE EMPLOYMENT: A CONTINUITY THEORY AND LIFE COURSE PERSPECTIVE | *International Journal of Aging & Human Development* | Geriatrics & Gerontology Psychology | Finland | Empirical | Bridge employment | The rapid aging of the workforce in most developed countries, and the strengthening presence of bridge employment among older employees, has brought about a need for a deeper theoretical and practical understanding of this employment phenomenon. This study examined the concept of bridge employment from a continuity theory and life course perspective. Several personal and job-related antecedents of 539 middle-aged and older U.S. Federal Government employees' intentions of full retirement or engagement in bridge employment were investigated. A multinomial logistic regression analysis provided support for most of the hypotheses on the antecedents of full retirement and overall bridge employment intentions and hence offered several practical implications for the U.S. Federal Government, as well as other governmental employers. Implications of these results are discussed from a continuity theory and life course perspective. |
| 26 | Nordenmark, M, 1999 | Employment commitment and psychological well-being among unemployed men and women | *Acta Sociologica* | Sociology | Sweden | Empirical | Gender | As regards employment commitment among both men and women, the results emphasize the psychosocial value of the former job, the activity level while unemployed and age. However, the results also indicate that age and family situation affect unemployed women's and men's levels of commitment to paid employment in different ways. Further, both unemployed men and women who are strongly motivated to find employment for non-financial reasons have significantly higher risks of poor mental well-being than those with lower commitments to employment. Finally, The results speak against the hypothesis that the high unemployment rates among certain categories of people are mainly a result of low motivation to be employed. |
| 27 | MORRELL, S; TAYLOR, R; (...); KERR, C, 1993 | SUICIDE AND UNEMPLOYMENT IN AUSTRALIA 1907-1990 | *Social Science & Medicine* | Public, Environmental & Occupational Health Biomedical Social Sciences | Australia | Ecological study | SDH | Female suicide rates were generally stable throughout the period, whereas those for males demonstrated sharp fluctuations with the peaks coinciding with times of high unemployment. The association between suicide and unemployment for 15-24 year old males was comparatively high for the recent period, 1966-1990. The increasingly youthful contribution to male suicide was demonstrated by a rise in the loss of life years during 1973-1984. Despite the inability of any investigation based on aggregate data to establish an unequivocable causal relationship, no evidence was detected to suggest that relatively high population levels of unemployment were not related to the occurrence of suicide. |

Retirement transitions (N=13)

| Research group | Author, year | Title | *Source* | [Field](http://ulrichsweb.serialssolutions.com/) | Geographical region (First author affiliation) | Type of study | Focus of paper | Main topic |
| --- | --- | --- | --- | --- | --- | --- | --- | --- |
| 1 | Zhang, T and Acs, Z, 2018 | Age and entrepreneurship: nuances from entrepreneur types and generation effects | *Small Business Economics* | Business & Economics | USA | Empirical | Occupational choice | Multilevel mixed-effect logistic regression models are estimated to examine the age effects in entrepreneur type propensities. Generational modification effects are compared for the same ages across neighboring generations by hierarchical age-period-cohort (HAPC) models. We find that entrepreneurial propensity rises with age until around 80. The propensity of novice (versus non-novice) and unincorporated (versus incorporated) entrepreneurs has a U-shaped age trend dipping around age 60, while the propensity of full-time (versus part-time) declines since age 30s. The propensity of incorporated (versus unincorporated) entrepreneurs declines from ages 44 to 51 for Gen-Xers, but not for Boomers; this propensity also declines faster for Boomers than for Traditionalists from ages 63 to 70. |
| 2 | Kenny, B and Rossiter, I, 2018 | Transitioning from unemployment to self-employment for over 50s | *International Journal of Entrepreneurial Behaviour & Research* | Business & Economics | Ireland | Intervention evaluation | Entrepreneurship | A framework specific to older unemployed individuals turning towards self-employment or entrepreneurship is proposed and tested in this paper. The framework proposes that individual and contextual antecedents influence the decision to become self-employed in later life and that the training, support and entrepreneurial experience helps to overcome barriers and shapes individual and societal outcomes. |
| 3 | Jones, DA and McIntosh, BR, 2010 | Organizational and occupational commitment in relation to bridge employment and retirement intentions | *Journal of Vocational Behavior* | Psychology | USA | Empirical | Bridge employment | Understanding the antecedents to retirement and bridge employment is important to older-aged adults who seek ways to smoothly transition to full retirement, and to organizations that benefit from retaining their highly skilled and most experienced workers, especially in occupations for Which labor shortages are projected. We tested the effects of affective, continuance, and normative commitment to organizations and to occupations on older-aged pharmacists' (N = 294) intentions to fully retire and to pursue three types of bridge employment. As hypothesized, criteria that were more organizationally focused (e.g., bridge employment in the same organization) were predicted more strongly by organizational, rather than occupational, commitment. For one type of bridge employment that was more occupationally focused bridge employment in a different field the hypothesized differential effects were supported, such that it was predicted more strongly by occupational, rather than organizational, commitment. We discuss the implications of our findings for theory, research, and practice |
| 4 | Soto-Simeone, A and Kautonen, T (2020) | Senior entrepreneurship following unemployment: a social identity theory perspective | *Review of Managerial Science* | Business & Economics | Chile | Empirical | 'active ageing' | The data highlight the relevance of non-monetary self-rewards-such as pursuit of autonomy, self-realisation, and wanting to feel active, useful and valuable-for senior entrepreneurs who start businesses under adverse conditions. |
| 5 | Ranzijn, R; Carson, E; (...); Price, D, 2006 | On the scrap-heap at 45: The human impact of mature-aged unemployment | *Journal of Occupational and Organizational Psychology* | Psychology Business & Economics | Australia | Qualitative study | Retirement transition, discouraged workers | The results suggest that there is a substantial 'lost generation' of mature-aged unemployed people who are characterized by shrinking horizons and impaired quality of life. The participants expressed frustration at being unable to contribute to society and support their own adult children. Inability to use their skills and talents, with consequent skill depreciation, can lead to what we label the 'peg-down phenomenon', an intermediate step between becoming unemployed and entering the ranks of the discouraged job-seekers that ultimately leads to a premature exit from the workforce. This cohort is different from other age groups of unemployed people because of the unique developmental characteristics of middle-aged people, compounded by financial and caring demands from both the younger and older generations. The paper concludes with policy recommendations, including expanding social inclusion policies to address the needs of this cohort and early intervention with more focused job-specific training. |
| 6 | Ulrich, LB and Brott, PE, 2005 | older workers and bridge employment: redefining retirement | *Journal of Employment Counseling* | Psychology | USA | Qualitative study | Bridge employment | The authors present a qualitative study that explored the transition experiences of older workers who retired from long-term careers and who were working in bridge jobs (i.e., transitional work between career employment and retirement). Using grounded theory methodology, the authors interviewed 24 older workers to learn why they decided to pursue a bridge job, how they made the transition, and what challenges they faced and benefits they received. The core theme from the study is that bridge employment redefines retirement. |
| 7 | Saba, T and Guerin, G, 2005 | Extending employment beyond retirement age: The case of health care managers in Quebec | *Public Personnel Management* | Business & Economics Public Administration | Canada | Cross sectional survey | Retirement decision | Based on a survey conducted on 402 older managers working in health care institutions in Quebec, this study sheds light on the new alternatives to traditional early retirement that organizations will have to take into account while considering the preferences and intentions of employees who are approaching retirement. |
| 8 | Noonan, AE, 2005 | "At this point now": Older workers' reflections on their current employment experiences | *International Journal of Aging & Human Development* | Geriatrics & Gerontology Psychology | USA | Thematic content analysis | Retirement | Thematic content analysis revealed a vast diversity in the concerns and experiences of contemporary older workers, with participants being actively involved in all of the traditional stages of work-life development. Findings suggest a deepened or more nuanced view of the principal meanings of work-in particular, identity and social interaction-and highlight several negative aspects of later-life employment such as pension insecurity, unemployment, age discrimination, not having found one's "niche," and chronic interpersonal difficulties with co-workers. Findings are discussed against the backdrop of a rapidly changing workplace and dramatically shifting work-retirement trajectories in which job stability and predictable retirement are less common. |
| 9 | Kim, S and Feldman, DC, 2000 | Working in retirement: The antecedents of bridge employment and its consequences for quality of life in retirement | *Academy of Management Journal* | Business & Economics | South Korea | Empirical | Aging, bridge employment | In this study, we used a continuity theory of aging to examine bridge employment. Excellent health, organizational tenure, and having working spouses and dependent children were positively associated with accepting bridge employment, and age and salary were inversely related to accepting such employment. Bridge employment was strongly related to both retirement satisfaction and overall life satisfaction. Volunteer work and leisure activity complemented bridge employment in facilitating adjustment to retirement. |
| 10 | Cahill, KE; Giandrea, MD and Quinn, JF, 2006 | Retirement patterns from career employment | *Gerontologist* | Geriatrics & Gerontology | USA | Cohort study | Bridge employment, gradual retirement | We found that a majority of older Americans with career jobs retire gradually, in stages, rather than all at once. We also found that the utilization of bridge jobs was more common among younger respondents, respondents without defined-benefit pension plans, and respondents at both the lower and upper ends of the wage distribution. Implications: Older Americans are now working longer than pre-1980s trends would have predicted. Given concerns about the traditional sources of retirement income (Social Security, employer pensions, and prior savings), older Americans may have to rely more on earnings. This article suggests that many are already doing so by moving to bridge jobs after leaving their career employment. |
| 11 | Chan, SW and Stevens, AH, 2001 | Job loss and employment patterns of older workers | *Journal of Labour Economics* | Business & Economics | USA | Longitudinal study | Displaced workers | This article uses data from the Health and Retirement Study to examine the employment patterns of workers aged 50 and above who have experienced an involuntary job loss. Hazard models for returning to work and for exiting post displacement employment are estimated and used to examine work patterns for 10 years following a job loss. Our findings show that a job loss results in large and lasting effects on future employment probabilities. Four years after job losses at age 55, the employment rate of displaced workers remains 20 percentage points below the employment rate of similar nondisplaced workers. |
| 12 | FELDMAN, DC, 1994 | THE DECISION TO RETIRE EARLY - A REVIEW AND CONCEPTUALIZATION | *Academy of Management Review* | Business & Economics | USA | Conceptual | early retirement | This article explores three interconnected decisions related to early retirement-the decision whether to leave a long-term job prior to age 65, the decision whether to accept bridge employment, and the decision whether to obtain bridge employment in the same industry or occupation as the last job-and the relationships among these three decisions and adjustment to retirement. In addition, this article examines the key variables that influence these three decisions, integrating previous research on individual-level, family-level, job- and career-related, organization-level, and environmental-level factors. The article concludes with an examination of methodological issues in the study of early retirement decisions and provides directions for future theory development. |
| 13 | Ruhm, CJ, 1990 | BRIDGE JOBS AND PARTIAL RETIREMENT | *Journal of Labr Economics* | Business & Economics | USA |  | BRIDGE JOBS AND PARTIAL RETIREMENT | The "job-stopping" process of older workers often includes some combination of postcareer "bridge" employment, partial retirement, and reverse retirement. Fewer than two-fifths of household heads retire directly from career jobs, over half partially retire at some point in their working lives, and a quarter reenter the labor force after initially retiring. In addition, postcareer employment is frequently located outside the industry and occupation of the career job, and there are important differences in postcareer labor force experiences by gender, permanent income, and career-job pension status. |
